# Single-Cell RNA Sequencing of the Rat Carotid Arteries Uncovers Potential Cellular Targets of Neointimal Hyperplasia

**DOI:** 10.3389/fcvm.2021.751525

**Published:** 2021-12-09

**Authors:** Xiao-Fei Gao, Ai-Qun Chen, Zhi-Mei Wang, Feng Wang, Shuai Luo, Si-Yu Chen, Yue Gu, Xiang-Quan Kong, Guang-Feng Zuo, Yan Chen, Zhen Ge, Jun-Jie Zhang, Shao-Liang Chen

**Affiliations:** ^1^Department of Cardiology, Nanjing First Hospital, Nanjing Medical University, Nanjing, China; ^2^Department of Cardiology, Nanjing Heart Centre, Nanjing, China; ^3^Department of Neurology, Medical School, Affiliated Drum Tower Hospital of Nanjing University, Nanjing, China

**Keywords:** in-stent restenosis, single-cell sequencing, vascular smooth muscle cell, transitional-cell, neointimal hyperplasia

## Abstract

**Aims:** In-stent restenosis (ISR) remains an Achilles heel of drug-eluting stents despite technical advances in devices and procedural techniques. Neointimal hyperplasia (NIH) is the most important pathophysiological process of ISR. The present study mapped normal arteries and stenotic arteries to uncover potential cellular targets of neointimal hyperplasia.

**Methods and Results:** By comparing the left (control) and right (balloon injury) carotid arteries of rats, we mapped 11 clusters in normal arteries and 11 mutual clusters in both the control and experimental groups. Different clusters were categorized into 6 cell types, including vascular smooth muscle cells (VSMCs), fibroblasts, endothelial cells (ECs), macrophages, unknown cells and others. An abnormal cell type expressing both VSMC and fibroblast markers at the same time was termed a transitional cell *via* pseudotime analysis. Due to the high proportion of VSMCs, we divided them into 6 clusters and analyzed their relationship with VSMC phenotype switching. Moreover, N-myristoyltransferase 1 (NMT1) was verified as a credible VSMC synthetic phenotype marker. Finally, we proposed several novel target genes by disease susceptibility gene analysis, such as Cyp7a1 and Cdk4, which should be validated in future studies.

**Conclusion:** Maps of the heterogeneous cellular landscape in the carotid artery were defined by single-cell RNA sequencing and revealed several cell types with their internal relations in the ISR model. This study highlights the crucial role of VSMC phenotype switching in the progression of neointimal hyperplasia and provides clues regarding the underlying mechanism of NIH.

## Introduction

It has been more than 30 years since the first stent implantation, and currently, percutaneous coronary intervention (PCI) has been widely adopted for most ischemic heart diseases. Despite technical advances in devices and procedural techniques, along with more intensive drug treatment, in-stent restenosis (ISR) following repeat coronary revascularization remains an Achilles heel of drug-eluting stents (DESs) (1–3). ISR is classically defined as luminal stenosis with more than 50% diameter narrowing of a stented coronary segment or within 5 mm of a stent edge. Traditionally, several biological factors, such as local inflammation, vascular smooth muscle cell (VSMC) phenotype switching, and delayed healing, are considered the main causes of ISR (4). The general view is that a normal artery consists of endothelial cells (ECs), VSMCs, fibroblasts, immune cells, and neurocytes (5, 6). The proportion of cell types inside the coronary artery changes after the development of atherosclerotic plaque and stent implantation due to the adaptive defense of blood vessels in response to internal and external stimuli. It is important to determine the changes in the proportion and status of these cells in the coronary artery after ISR, which could help us understand the underlying mechanisms of ISR. According to previous studies, neointimal hyperplasia (NIH) with VSMC migration and proliferation remains the most essential pathophysiological mechanism of ISR (7–9). However, there is no optimal method for in-depth analysis of cell types, composition, and properties in restenotic tissues. Thus, use of a novel methodology to investigate the mechanism of NIH is warranted. Therefore, the present study was designed to map the full view of normal arteries and stenotic arteries with an established rat carotid artery balloon injury model to further explore the underlying mechanism of NIH by using advanced single-cell technologies.

## Methods

Expanded methods are provided in the Supplementary Materials online.

### Animal

Eight-week-old mature male Sprague-Dawley (SD, Hsd, Harlan) rats from the Animal Core Facility of Nanjing Medical University (Nanjing, China) were used in our experiment. Rats were housed in a temperature-controlled room with a 12 h light/dark cycle and free access to fresh water and food. All animal procedures were approved by the Experimental Animal Care and Use Committee of Nanjing Medical University.

### Rat Carotid Artery Balloon Injury Model

The rat carotid artery balloon injury model is the most common *in vivo* model widely used to study ISR. This approach consists of isolating a segment from the right common carotid artery in SD rats under general anesthesia (pentobarbital sodium, 60 mg/kg, i.p.), creating an arteriotomy incision in the external carotid branch followed by inserting a balloon catheter (Fogarty, 12A0602F, 0.67 mm, Edwards Lifesciences) into the common carotid artery, repeated balloon inflation and pulling back five times to imitate percutaneous transluminal coronary angioplasty (PTCA), and finally removal of the catheter with external carotid ligation (10). The rats were sacrificed with an overdose of pentobarbital sodium (200 mg/kg, iv) at 28 days after the procedure, and their left (control group) and right (case group) carotid arteries (Supplementary Figure 1A) were subsequently collected for further study. We used HE staining to identify the success of the model (Supplementary Figure 1B).

### Collecting Cells and Single-Cell RNA Sequencing

**Figure 1:**
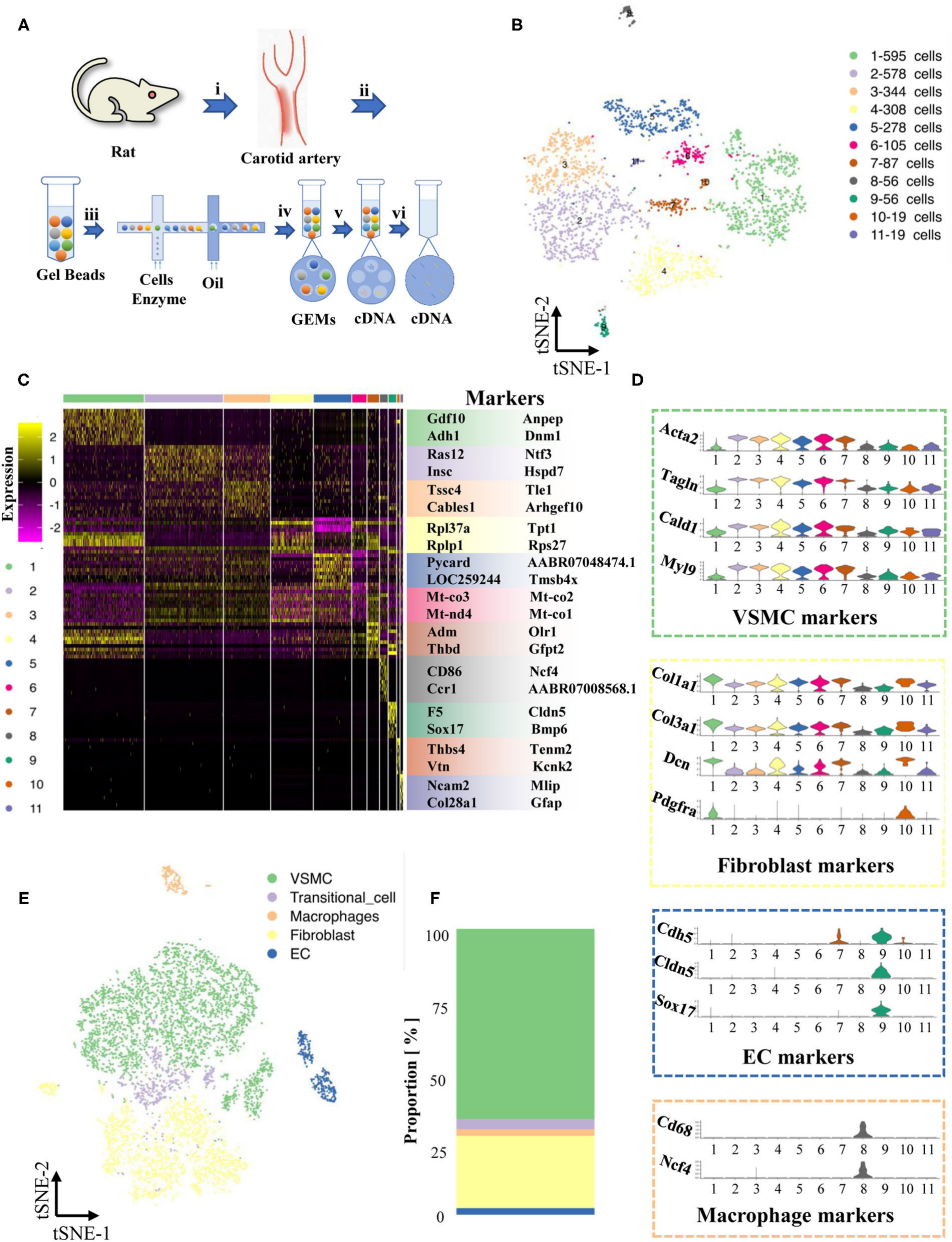
Maps of single-cells in normal cartoid arteries. **(A)** (i). A surgery performed on rats and separated cartoid arteries. (ii). Prepared 10x barcoded gel beads. (iii). Wrapped the beads and cells with cell barcode in droplets, collected the droplets with cells, and then lysed the cells in the droplets. (iv). Collected single cell GEMs. (v). RT-PCR. (vi). Pool removed oil. **(B)** TSNE of single cells in normal cartoid arteries. **(C)** Differentially expressed genes (Top 10) of 11 clusters. **(D)** Expression of classical markers (VSMC, fibroblast, EC and macrophage) in 11 clusters. **(E)** Classification of 11 clusters into 5 cell types (VSMC, fibroblast, EC, transitional-cell, macrophage). **(F)** Proportion of each cell type in tSNE.

To avoid data variation incurred by sex differences, only two male SD rats were selected for the study. To capture single cells, the common carotid arteries were washed with PBS twice and stored in MACS tissue storage solution (Cat#: 130-100-008). Tissues were digested by 0.25% Trypsin-EDTA (1^*^, Gibco) and 0.1% collagenase (1^*^, Gibco). Single-cell RNA sequencing with the 10x Genomics platform was performed by a commercial service (Shanghai OE Biotech Co., Ltd., China; Figure 1A). Briefly, it uses microfluidic technology to wrap the beads and single cells with Cell Barcodes in droplets, lyses the cells in the droplets to connect the mRNA in the cells to the Cell Barcodes on beads, and finally forms single-cell GEMs. Reverse transcription was performed with the droplets to construct a cDNA library. The sample source of the target sequence is distinguished by the sample index on the library sequence.

## Results

### Single-Cell RNA-Seq of Normal Rat Carotid Artery Cells

We first concentrated the normal carotid artery (control group), which has never been mapped previously. The two whole left carotid arteries were enzymatically digested, and then scRNA-seq libraries were built with the 10x Genomics platform.

A total of 2,445 cells were captured by quality control and visualized in t-SNE dimensionality reduction plots within 11 clusters (Supplementary Table 1, Supplementary Figure 2, Figure 1B). To classify these 11 clusters into known cell types, we filtered out their highly expressed genes and labeled them based on known marker genes (Figure 1C). We also examined recognized cell-type markers, such as VSMCs (Acta2, Tagln, Cald1, Myl9), fibroblasts (Col1a1, Col3a1, Dcn, Pdfra), ECs (Cdh5, Cldn5, Sox17), and macrophages (Cd68, Ncf4) (Figure 1D). The differentially expressed genes of cluster 11 (only 19 cells) were not specific and could not match the existing recognized cell types; thus, we performed our analysis after excluding cluster 11. A new t-SNE dimension reduction plot of 5 cell types showed the different proportions of cell types, including ECs, VSMCs, fibroblasts, macrophages and unknown cells (Figure 1E). Notably, VSMCs and fibroblasts were the major cell types in the normal carotid artery (Figure 1F).

### Transitional Cells Between VSMCs and Fibroblasts

**Figure 2:**
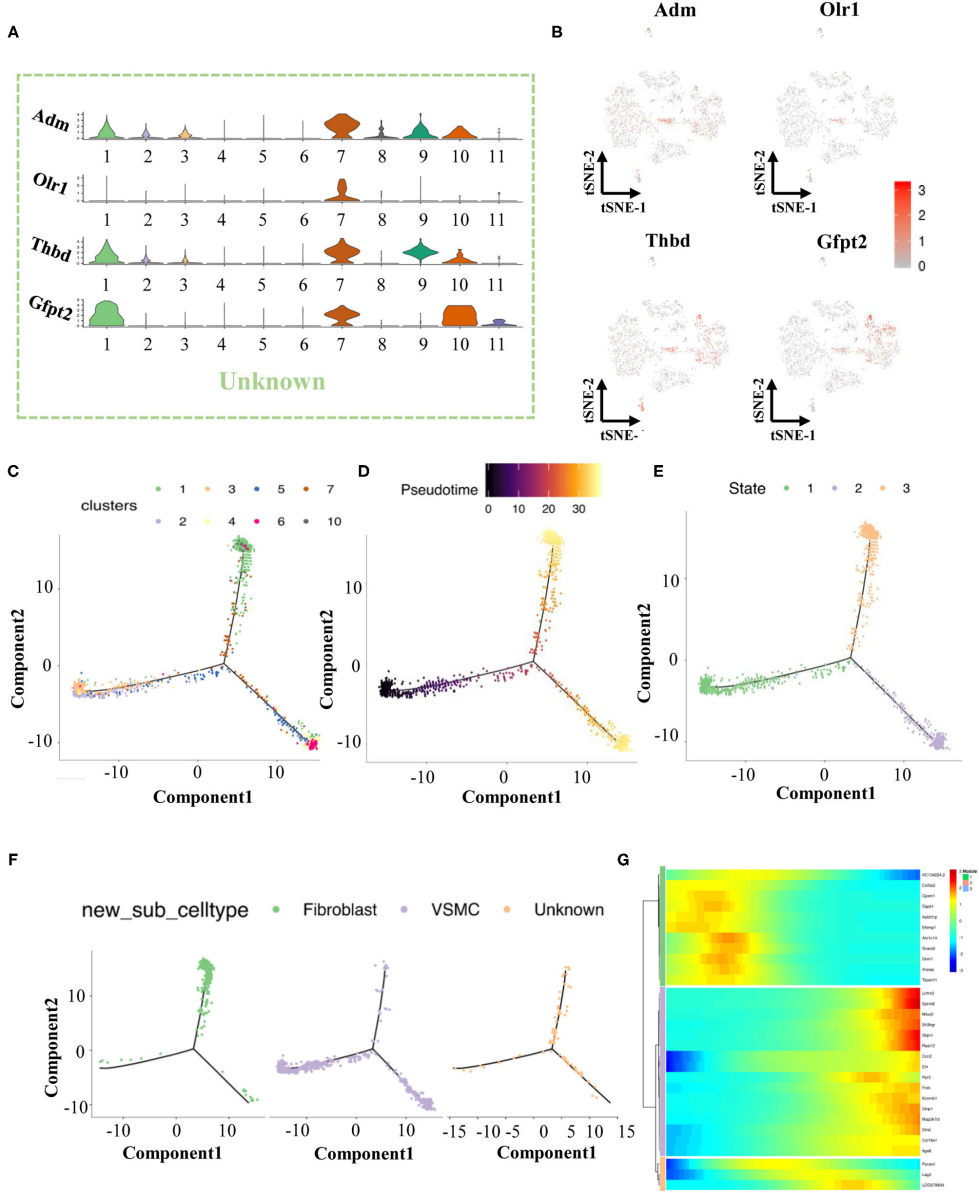
The finding of transitional-cells. **(A)** Violin illustrations showed differentially expressed genes (Top 4) in transitional-cells. **(B)** Scatter plot showed enrichment of differentially expressed genes in transitional-cells. **(C)** Pseudotime analysis of VSMCs, fibroblasts and transitional-cells. **(D)** Coloring for differentiation time. **(E)** Coloring for the state of differentiation. **(F)** Split the branches. **(G)** Heat map of gene expression in cells of different differentiation states.

We found that cells in cluster 7 highly expressed marker genes from both VSMCs and fibroblasts at the same time (Figure 1D), but these cells in cluster 7 also had their own specific markers (Adm, Olr1, Thbd, Gfp2, Figures 2A,B). Previous studies have reported that the VSMC phenotype might switch to a fibroblast phenotype in some situations (11, 12). Therefore, we tried to investigate the roles of cluster 7 during the process of VSMC phenotype switching *via* the current model.

We performed pseudotime analysis on VSMCs, fibroblasts, and unknown cells consisting of clusters 1-7 and 10. According to the degree of differentiation, cells were classified into three trajectories (states 1, 2, and 3) in pseudotime analysis (Figures 2C–F). Obviously, VSMCs were mainly distributed in state 1 and state 2, while fibroblasts were distributed in state 2. Previous evidence demonstrated that VSMCs had two important phenotypes in the vessel wall, including a contractile phenotype and a synthetic phenotype (13, 14). Fully differentiated/contractile VSMCs, responsible for vascular tone regulation, could be transformed into dedifferentiated/synthetic VSMCs to acquire proliferation, migration and synthesis abilities after vascular injury (13). According to the marker gene analysis, VSMCs in state 2 were considered contractile VSMCs, and VSMCs in state 1 could be regarded as synthetic VSMCs. The unknown cells in cluster 7 were scattered in states 2 and 3 by pseudotime analysis, indicating that these cells could play crucial roles in phenotype switching between contractile VSMCs and fibroblasts. Finally, cells in cluster 7 were termed transitional cells due to the co-expression of cell-type markers of VSMCs and fibroblasts. Moreover, we selected and analyzed the top 10 genes of VSMCs, fibroblasts, and transitional cells through pseudotime analysis, as summarized in Figure 2G.

### Maps of Cells in Stenotic Arteries

**Figure 3:**
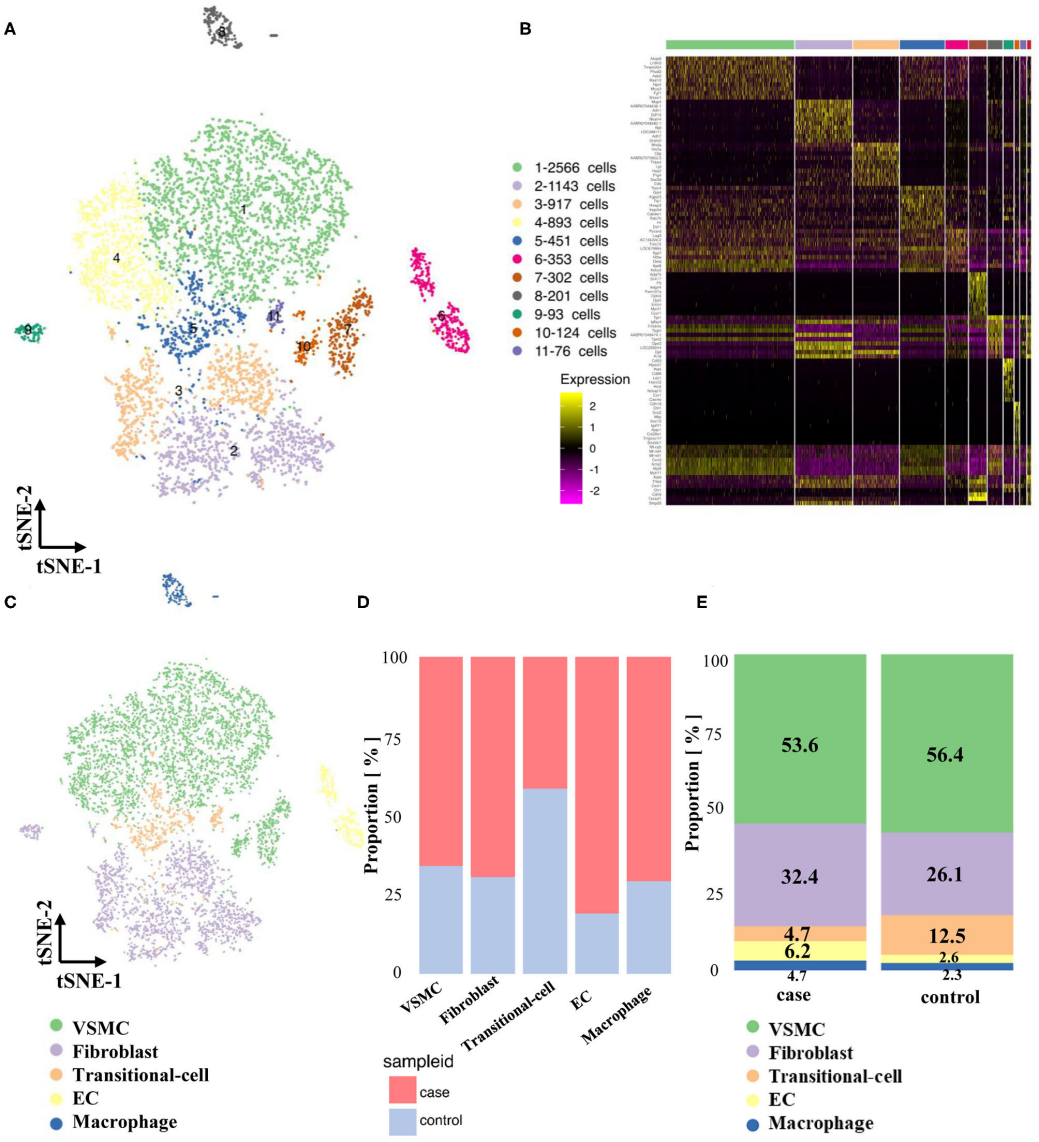
Maps of single-cells in both normal and experimental carotid arteries. **(A)** TSNE of single cells in normal and experimental cartoid arteries. **(B)** Differentially expressed genes (Top 10) of 11 clusters. **(C)** Classification of 11 clusters into 5 cell types (VSMC, fibroblast, EC, transitional-cell, macrophage). **(D)** Comparison of proportion in different cell types. **(E)** Comparison of proportion in case and control groups.

A total of 4,674 cells in the stenotic carotid artery (case group) were acquired after removing unqualified cells from 5,656 cells by quality control. Specific genes from a total of 11 clusters were identified and used to construct a heatmap (Supplementary Figure 2, Figures 3A,B). We classified all of these cells into five cell types according to recognized markers of VSMCs, fibroblasts, ECs and macrophages (Figure 3C). We used the abovementioned strategy to verify the transitional cells and found a similar result. Obviously, the proportion of different cell types changed considerably between the normal artery and stenotic artery. The proportion of fibroblasts, ECs, and macrophages increased in the stenotic artery compared to the normal artery, while the proportion of VSMCs and transitional cells decreased in the stenotic artery (Figures 3D,E).

Pseudotime analysis of VSMCs, fibroblasts and transitional cells (Supplementary Figures 3A,B) showed that these cells could be classified into 5 states: state 1, 2, and 3 fibroblasts; state 4 synthetic VSMCs; and state 5 contractile VSMCs (Supplementary Figures 3C,D, 4). Interestingly, transitional cells in the stenotic artery were distributed in state 4 (synthetic VSMCs), which was different from their co-expression with markers of VSMCs and fibroblasts in the normal artery. This novel finding indicated that contractile VSMCs could switch to both a fibroblast-like phenotype and a synthetic VSMC phenotype in the stenotic artery after balloon injury. Pseudotime analysis and heatmaps of differentially expressed genes in VSMCs, fibroblasts and transitional cells are shown in Supplementary Figures 5–11.

### Differences in VSMCs Between Normal and Stenotic Arteries

**Figure 4:**
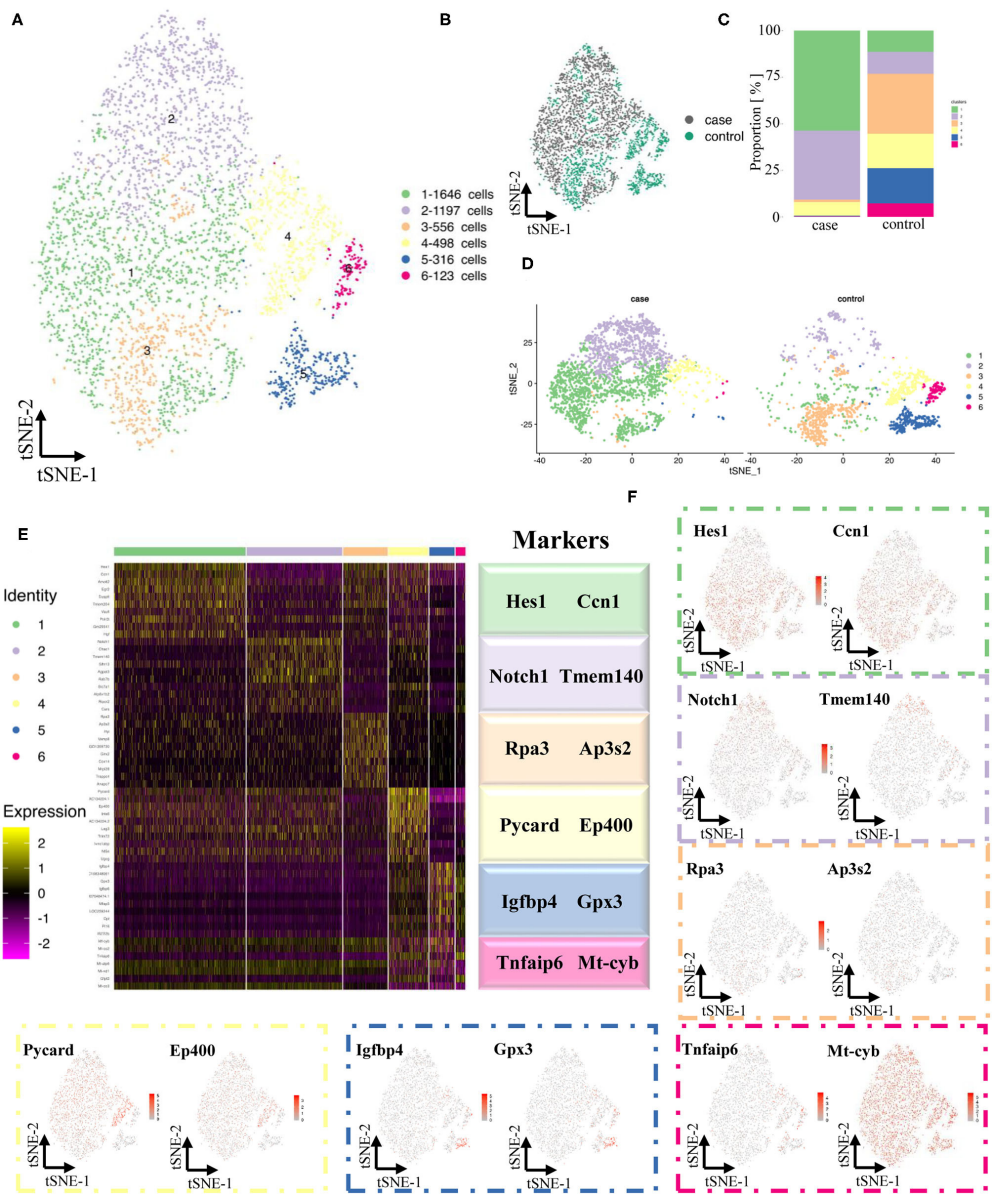
Maps of VSMCs in both normal and experimental carotid arteries. **(A)** TSNE of sing cells of VSMCs. **(B)** Distribution of case and control groups in tSNE. **(C)** Comparison of proportion in case and control groups. **(D)** Comparison of sing cells of case and control groups in tSNE. **(E)** Differentially expressed genes (Top 10) of 6 clusters. **(F)** Expression of classical markers (VSMC, fibroblast, EC and macrophage) in 11 clusters.

Contractile VSMCs, the major cell type in the artery (Figure 3E), are important for vessel contraction/dilation and regulating hemodynamics (14, 15). Thus, the differences in VSMCs between normal and stenotic arteries were analyzed in depth to explore the mechanism of ISR. VSMCs were isolated and then divided into 6 distinct clusters (Figures 4A,B) based on gene enrichment analysis. From Figures 4C,D, we found that the proportion of VSMCs in these 6 clusters varied greatly, and then several differentially expressed genes were selected to identify these 6 different clusters. Markers of cluster 1 (Hes1, Ccn1), cluster 2 (Notch1, Tmem140), cluster 3 (Rpa3, Ap3s2), cluster 4 (Pycard, Ep400), cluster 5 (Igfbp4, Gpx3), and cluster 6 (Tnfaip6, Mt-cyb) are summarized as dot plots (Figures 4E,F). GO and KEGG analyses were also performed to dig deeper into the functions and interactions of VSMCs (Supplementary Figures 12–19).

### Phenotype Switching Among 6 Clusters of VSMCs

**Figure 5:**
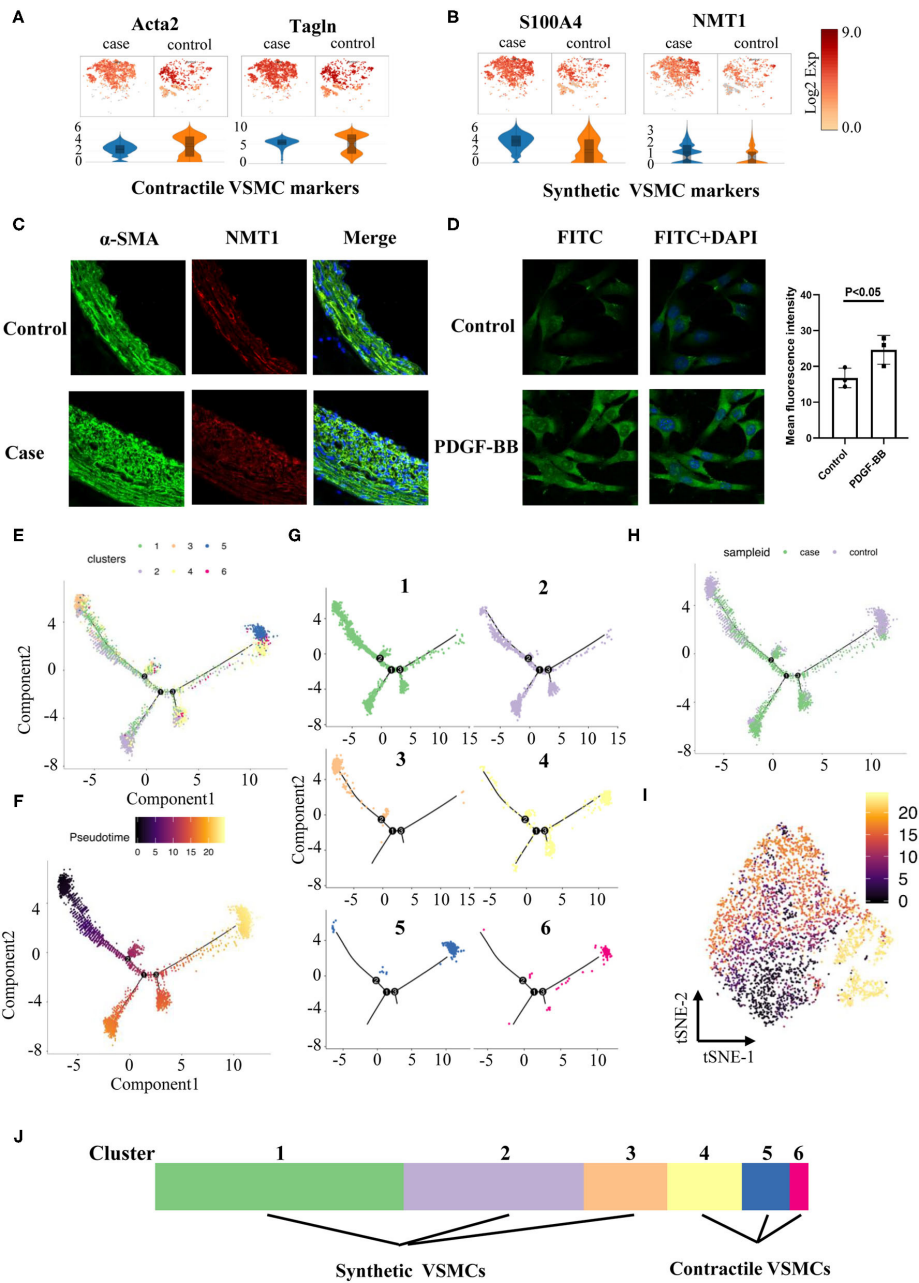
Phenotype switching in VSMCs. **(A)** Dot plots and violin illustrations of contractile makers of VSMC. **(B)** Dot plots and violin illustrations of synthetic makers of VSMC. **(C)** Immunofluorescence images of slices of rats' cartoid arteries in case and control groups. **(D)** Immunofluorescence images of moves under pretreatment (PDGF-BB) and control groups. **(E)** Pseudotime analysis of VSMCs. **(F)** Coloring for differentiation time. **(G)** Split the branches. **(H)** Coloring for case and control groups. **I**. Coloring for differentiation time in tSNE. **(J)** Classified 6 clusters into contractile VSMCs and synthetic VSMCs.

Contractile VSMCs could switch into a synthetic phenotype through dedifferentiation in response to vascular injury, and they could also regain the contractile property *via* differentiation under certain conditions (13, 14). We assigned each cluster to two phenotypes based on recognized contractile (Acta2, Tagln, Figure 5A) or synthetic VSMC markers (S100A4, Figure 5B). We also discovered an extra novel gene, N-myristoyltransferase 1 (Nmt1), after comparing our data with classical markers, which was previously reported to participate in the development of cancers (16). Higher expression of Nmt1 was found in the stenotic artery, indicating that it might be a new marker in our ISR model (Figure 5C) but also in general synthetic VSMCs. To verify the findings above, we used platelet-derived growth factor BB [PDGF-BB, a classical cytokine promoting VSMCs dedifferentiated into the synthetic phenotype (17)] to treat mouse aortic vascular smooth muscle cells (MOVAS) at 20 ng/ml and found that MOVAS expressed a higher level of NMT1 after PDGF-BB treatment (Figure 5D).

Pseudotime analysis found that VSMCs in different clusters were separated and distributed in various routes, which were linked *via* three nodes (Figures 5E,F). Six clusters of VSMCs (Figure 5G) indicated that they were distributed at different stages: clusters 4, 5, and 6 might pause at advanced differentiation stages; clusters 1, 2, and 3 might pause at early stages. Meanwhile, we found that VSMCs in the case group showed a more extreme distribution in pseudotime routes but a disseminated distribution in the control group (Figure 5H). Finally, we identified cluster 4, 5, and 6 cells as contractile VSMCs and cluster 1, 2, and 3 cells as synthetic VSMCs (Figure 5J).

It has been reported that the Notch-Hes1 pathway, a pro-differentiation pathway, plays an essential role in the differentiation of many cell types (18). Notch1 increases after vascular injury, and inactivation of Notch1 might reduce neointimal hyperplasia (19). In our study, Notch1 and Hes1 were highly expressed in synthetic clusters 1 and 2 (Figure 4E), indicating that these two genes might be synthetic VSMC-related genes.

### Differences in Fibroblasts and ECs Between Normal and Stenotic Arteries

Our data demonstrated that fibroblasts, mainly distributed in the adventitia of blood vessels, could proliferate greatly during the progression of neointimal hyperplasia (Supplementary Figure 20). A heatmap could help us identify specific markers of these proliferous fibroblasts and provide a new strategy to distinguish fibroblasts in the normal position or the neointima (Supplementary Figure 21). Pseudotime analysis demonstrated that cells in cluster 3 might be proliferous fibroblasts in the case group (Supplementary Figures 22–25). In addition, GO terms and KEGG terms reminded us of the relationship between differentially expressed and specific functions (Supplementary Figures 26–33).

Originally, our model indicated damaged endothelial cells and VSMCs cause intimal hyperplasia. The increase in ECs might be caused by the appearance of EC-like VSMCs or re-endothelialization (20). Total ECs were clustered, indicating that cells in cluster 2 acted as EC-like VSMCs and that cells in cluster 1 could be related to re-endothelialization (Supplementary Figures 34–37). GO terms and KEGG terms also reminded us of the functional changes between the case and control groups (Supplementary Figures 38–45).

### Disease Susceptibility Gene

**Figure 6:**
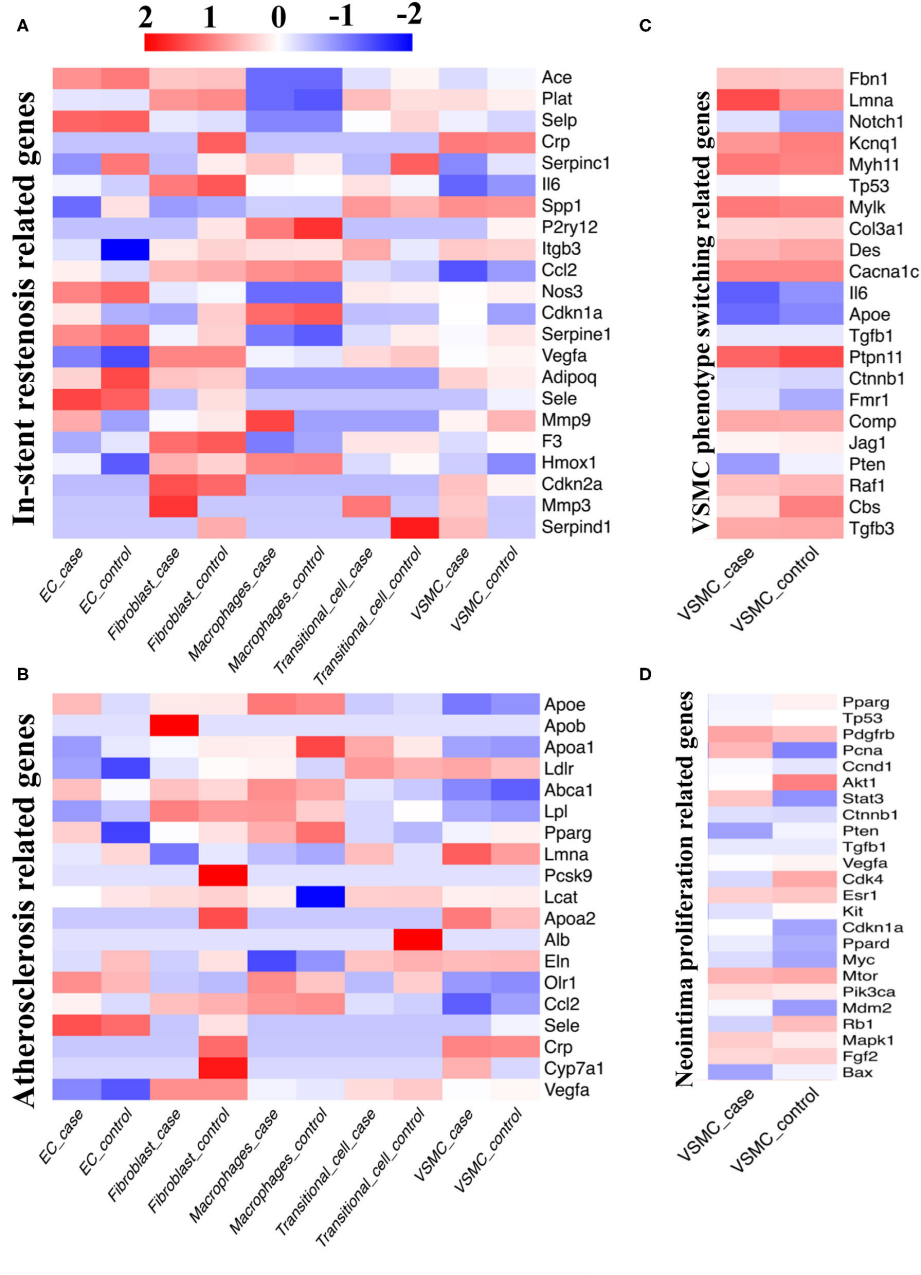
Comparison between single-cell database and disease susceptibility gene databases. **(A)** Our single-cell sequencing compared with database of in-stent restenosis related genes. **(B)** Our single-cell sequencing compared with database of atherosclerosis related genes. **(C)** Our single-cell sequencing compared with database of VSMC phenotype switching related genes. **(D)** Our single-cell sequencing compared with database of neointima proliferation related genes.

To verify the similarity between our model and real-world disease, we compared four sets of independent human disease susceptibility genes from GeneCards (https://www.genecards.org/) (ISR-related genes, VSMC phenotype switching-related genes, atherosclerosis-related genes, neointima proliferation-related genes) with our data to determine the expression abundance of previously recognized disease susceptibility genes (Figures 6A–D).

When analyzing ISR-related genes, the Ace, Selp, Nos3, Serpine1, and Sele genes were highly expressed in ECs; the Plat, Il6, Vegfa, F3, and Cdkn2a genes were highly expressed in fibroblasts; the P2ry12, Ccl2, Cdkn1a, and Hmox1 genes were highly expressed in macrophages; the Spp1 gene was highly expressed in transitional cells; and the Crp and Spp1 genes were highly expressed in VSMCs (Figure 6A). When analyzing atherosclerosis-related genes, we screened out high expression of the Olr1 and Sele genes in ECs; the Lpl, Ccl2, and Vegfa genes in fibroblasts; the Apoe, Abca1, Pparg, and Ccl2 genes in macrophages; the Ldlr and Eln genes in transitional cells; and the Ldlr, Lmna, Apoa2 and Crp genes in VSMCs (Figure 6B). Afterwards, we focused on VSMCs and found that the Lmna, Myh11, Mylk, Cacna1c, and Ptpn11 genes were intensely related to VSMC phenotype switching; the Pdgfb and Mtor genes were closely related to neointimal proliferation.

The matrix metallopeptidase (Mmp) gene family related to ISR was highly expressed in the stenotic artery but expressed at low levels in the control group (Mmp3 in fibroblasts; Mmp9 in macrophages; Mmp3 in transitional cells; Figure 6A). Meanwhile, several genes related to ISR exhibited high expression in the case group and low expression in the control group, such as Serpinc1 in ECs, Crp in fibroblasts, and Serpinc1 and Serpind1 in transitional cells (Figure 6A). When compared with the control group, several genes related to atherosclerosis were differentially expressed in the case group (Up: Apob in fibroblasts; Cyp7a1 in VSMCs; Down: Pcsk9, Apoa2, Crp, Cyp7a1 in fibroblasts; Apoa1 in macrophages; Alb in transitional cells, Figure 6C). We also found that the expression of several VSMC genes related to neointimal proliferation changed considerably with the progression of disease (Up: Pcna; Down: Akt1, Cdk4, and Rb1).

By comparing the susceptibility gene databases with our sequencing data, we obtained some results similar to previous studies and verified them in an *in vivo* study. Moreover, we discovered several novel genes based on the current model, such as Cyp7a1 and Cdk4, which should be further validated.

### Cell-to-Cell Communication: Receptor Ligand Analysis

**Figure 7:**
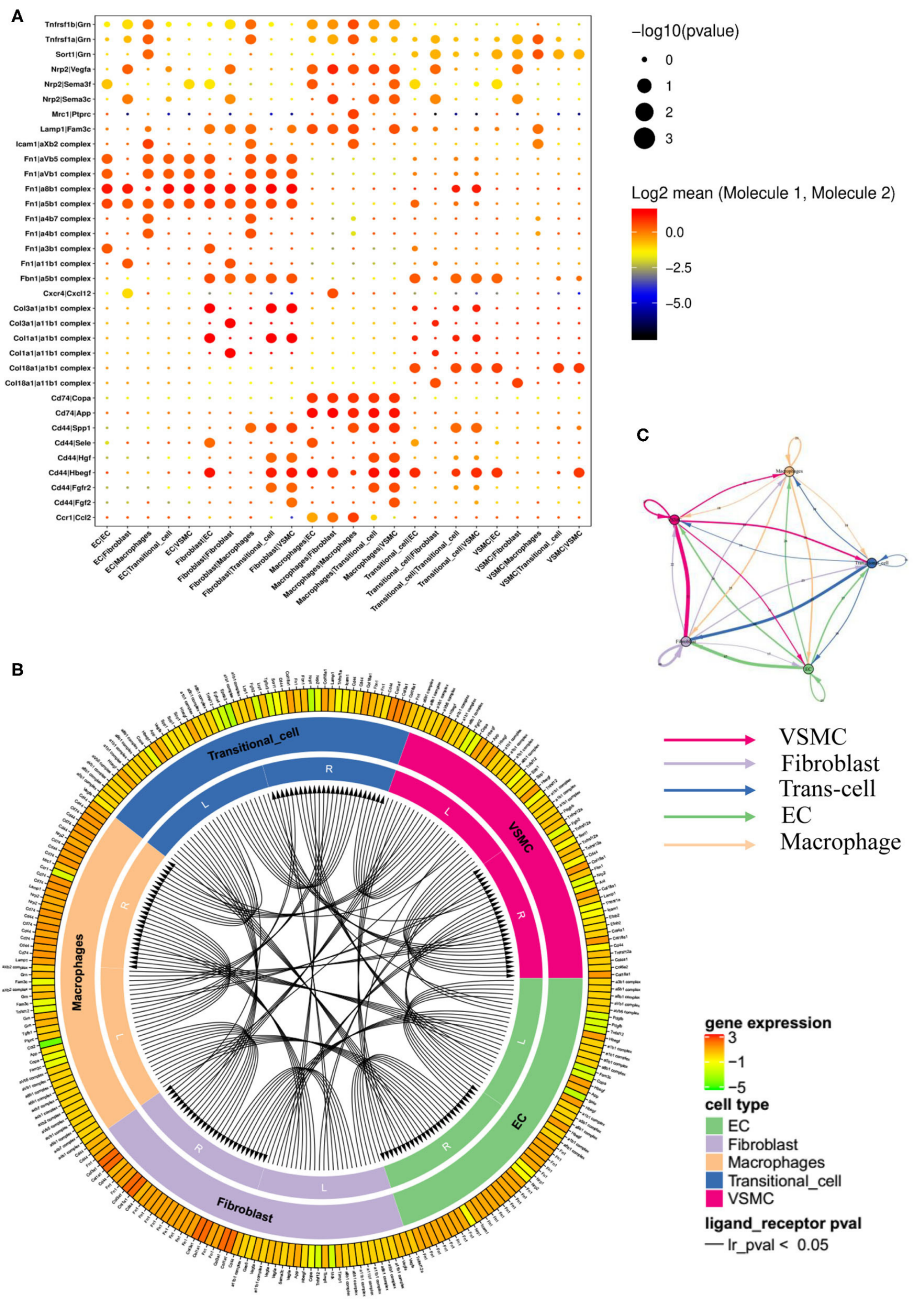
Networks of ligands and receptors in single-cells of case group. **(A)** Dot plot of receptors and ligands analysis. **(B)** Intercellular communication of different cell types. **(C)** Quantitative figure of intercellular communication.

We performed receptor ligand analysis in the normal artery and stenotic artery (Figure 7A, Supplementary Figure 46). In the normal artery, Fn1, especially Fn1/α8β1 integrin, was enriched in ECs. Col1a1 and Col3a1, whose ligands are α1β1 integrin and α11β1 integrin, were enriched in fibroblasts. Moreover, macrophages were associated with the high expression of Cd74, Cd44/Copa, App, and Hbegf. Col1a1/α1β1 integrin and Col1a1/α11β1 integrin were also enriched in transitional cells. However, the gene expression of ligands in VSMCs was low.

Of note, the expression levels of receptors and ligands in the stenotic artery changed considerably compared with those in the normal artery (Supplementary Figure 15). Fn1/α8β1 integrin, Fn1/α5β1 integrin, Fn1/αVβ1 integrin, and Fn1/αVβ5 integrin were all enriched in ECs. Fn1, Col3a1, and Cd44 (ligands) and αVβ1, αVβ5, α8β1, α5β1, α1β1 integrin, SPP1, and Hbegf (receptors) were enriched in fibroblasts. Nrp2/Vegfa, Cd74/Copa, Cd74/App, and Cd74/Hbegf were enriched in macrophages. Moreover, transitional cells seemed to have high enrichment levels of Col18a1/α1β1 integrin and Cd44/Hbegf. The ligand and receptor circuit diagram suggested decreased communication between VSMCs and ECs (Figures 7B,C). In addition, ligands from fibroblasts decreased relative to the control group.

## Discussion

To the best of our knowledge, this is the first study to investigate the mechanisms of ISR by single-cell RNA sequencing. There are several main findings in our study. First, we found traditional cells between VSMCs and fibroblasts in both normal and stenotic arteries. The specific key genes of these novel cells were also selected by heat map analysis and in a dynamic change trend graph of pseudotime analysis. Second, the phenotype of VSMCs, the major component of blood vessels, switched greatly in the stenotic artery, and we further classified the VSMCs into 6 clusters based on gene enrichment analysis. Finally, disease susceptibility gene analysis confirmed the association of those classical genes and ISR by comparing our data with four previous databases, and we also proposed several novel related target genes, such as Cyp7a1 and Cdk4, which should be validated in future studies.

DES has completely replaced bare-metal stents to be used in ischemic heart disease in daily clinical practice due to the proliferation inhibition effects of coating agents (21), but the relatively high occurrence of restenosis (5–10%) after implantation of DES still cannot be prevented, with a direct financial burden on patients and medical insurance (1). It is warranted to use a novel methodology to investigate the underlying mechanisms of ISR. Single-cell RNA sequencing is a novel bioanalysis technology in recent years that has been extensively used in the field of cancer but less in the cardiovascular field (22). Thus, we conducted the present study and hope to find new clues to explore the mechanisms of ISR in depth.

VSMCs have more plasticity than any other cell type in blood vessels. In atherosclerosis models, VSMC-derived foam cells and macrophage-like VSMCs play major roles within the arterial wall (15). However, our data indicated that specific markers of macrophages showed low expression in VSMCs and ensured the purity of VSMCs. In addition, a previous study revealed that VSMCs could switch into osteoblast-like cells, fibroblast-like cells and senescent VSMCs (23). Wirka et al. reported fibroblast-like cells in atherosclerotic lesions that broke inherent cognition (12). Our data also proved that phenotype switching exists between VSMCs and fibroblasts and found a new cell type: transitional cells. During the transition from VSMCs to fibroblasts, inflammatory factors dropped sharply, and cyclins indicated cell cycle arrest, suggesting that this transition has a protective effect on VSMCs. From another perspective, the transition from fibroblasts to VSMCs means that the elasticity and compliance of hyperplastic tissues are reduced, which may have a certain impact on the long-term prognosis of diseased blood vessels. Therefore, the in-depth investigation of transitional cells is particularly important. By regulating the number and homeostasis of transitional cells, we might seek benefits and avoid disadvantages at the same time.

VSMCs, a major component of blood vessels, were clustered into 6 groups. Obviously, we observed changes in proportions between 6 clusters. We tried to uncover the mechanism of ISR by explaining these changes between 6 clusters during neointima proliferation. In recent years, phenotype switching/modulation of VSMCs has always been a research hotspot and is used to analyze a variety of VSMC-related disease models. Pseudotime analysis helped us distinguish contractile and synthetic VSMCs by the degree of differentiation. Unlike pseudotime analysis of single-cell sequencing, we usually used specific markers to identify different cell types of VSMCs *in vitro*. Nonetheless, classical markers of contractile or synthetic VSMCs are not applicable to all models. For example, secreted phosphoprotein 1 (SPP1)-encoded protein osteopontin (OPN) (24) of synthetic VSMCs, an extracellular matrix-related component that has been frequently reported in VSMC phenotype switching, showed hardly any differences between the case and control groups. Therefore, it is necessary to select several genes suitable for ISR models. Nmt1 might be a new marker in both ISR models *in vivo* and VSMCs *in vitro*. To some extent, Nmt1 is specific not only to the ISR model but also to models related to VSMC phenotype switching, which need more verification.

Moreover, by comparing the database of disease-related susceptibility genes, we revealed target cells of these disease-related susceptibility genes *in vivo*. The expression of disease-related susceptibility genes changed obviously between the case and control groups, which indicated that VSMCs played major roles in neointimal proliferation. Jason LJ et al. reported that Mmp3 mediated the activation of Mmp9, which was required for neointimal proliferation and VSMC migration (25). The serpin gene family, with antithrombin and antiproliferation functions, has been reported to play a protective role in blood vessels (26). As mentioned above, fibronectin1 (Fn1) was found to be a representative ligand and regulate integrins, as suggested by previous studies (27, 28). However, the communication between Fn1 and integrins was enhanced with the progression of ISR and was mainly located in ECs and fibroblasts. In addition, we benefited from the analysis of receptors and ligands. Signals of receptors and ligands aggregated in ECs and macrophages, which was opposite to the results obtained for disease-related susceptibility genes. The results highlighted Fn1/integrins in ECs and fibroblasts and Cd44 in macrophages and showed obvious changes in Fn1/integrins and Cd44 between the case and control groups. However, the number of receptors and ligands of fibroblasts rather than ECs or macrophages was obviously reduced in total.

There are several limitations in our study. First, the rat carotid artery balloon injury model is the most common *in vivo* model widely used to study ISR, but a gap still exists. Second, to obtain live cells for single-cell sequencing, we chose balloon-injured rat carotid arteries instead of frozen human restenotic coronary artery specimens in cold storage. Third, two cases and two controls were selected due to the limited research funding and time, and a relatively small sample size might produce bias. However, the first applied single-cell sequencing in the ISR model could provide novel clues for further study.

In conclusion, maps of heterogeneous cellular landscapes, especially transitional cells, in the carotid artery were defined by single-cell RNA sequencing and revealed several cell types with their internal relations in the ISR model. This study highlights the crucial role of VSMC phenotype switching in the progression of ISR, and we also proposed several novel related target genes, such as Cyp7a1 and Cdk4, providing clues regarding the underlying mechanism of ISR.

## Data Availability Statement

The datasets presented in this study can be found in online repositories. The names of the repository/repositories and accession number(s) can be found at: https://www.ncbi.nlm.nih.gov/geo/, GSE174098.

## Ethics Statement

The animal study was reviewed and approved by Experimental Animal Care and Use Committee of Nanjing Medical University.

## Author Contributions

X-FG and A-QC performed experiments and wrote the manuscript. Z-MW and FW prepared the figures. SL, S-YC, and YG prepared the Supplementary Materials. YC and ZG prepared the table. J-JZ and S-LC provided the idea and revised the manuscript. All authors have agreed to the published version of the manuscript.

## Funding

This study was funded by the National Natural Science Foundation of China (NSFC 81970307 and 81801147), and was jointly supported by Six Talent Peaks Project of Jiangsu Province (2019-WSN-156), Social Development Project of Jiangsu Province (BE2019616), Jiangsu Commission of Health (H2019077), Nanjing Commission of Health (ZKX19027), and Nanjing Health Youth Talent Training project (QRX17017).

## Conflict of Interest

The authors declare that the research was conducted in the absence of any commercial or financial relationships that could be construed as a potential conflict of interest.

## Publisher's Note

All claims expressed in this article are solely those of the authors and do not necessarily represent those of their affiliated organizations, or those of the publisher, the editors and the reviewers. Any product that may be evaluated in this article, or claim that may be made by its manufacturer, is not guaranteed or endorsed by the publisher.
