# Supplementary material for: Single-Cell RNA Sequencing of the Rat Carotid Arteries Uncovers Potential Cellular Targets of Neointimal Hyperplasia
Source: Front Cardiovasc Med. 2021 Dec 9;8:751525. doi: 10.3389/fcvm.2021.751525 (PMC8697976; doi:10.3389/fcvm.2021.751525)
Supplement: Supplementary file 1 [file Data_Sheet_1.pdf]

Supplemental materials

Catalogue:

- **Methods:**
  - **Analysis and quality control**
  - **Data dimension reduction and differentially expressed genes**
  - **Gene ontology enrichment analysis**
  - **Kyoto Encyclopedia of Genes and Genomes**
  - **Pseudotime analysis**
  - **Ligand-receptor networks**
  - **Cell culture**
  - **Immunofluorescence**
- **Supplemental figure 1: Cartoid arteries of an experimental model.**
- **Supplemental figure 2: Quality control of single-cell database.**
- **Supplemental figure 3-4: Pseudotime analysis of VSMCs, fibroblasts and transitional-cell in total group.**
- **Supplemental figure 5-10：Pseudotime analysis of top10 differentially expressed genes of fibroblasts, transitional-cells and VSMCs in case and control groups.**
- **Supplemental figure 11：Hotmap of top10 differentially expressed genes of fibroblasts, transitional-cells and VSMCs.**
- **Supplemental figure 12-16: GO analysis of total-VSMCs.**
- **Supplemental figure 17-19: KEGG analysis of total-VSMCs.**
- **Supplemental figure 20: TSNE of total-fibroblasts.**
- **Supplemental figure 21: Top10 markers of different clusters in fibroblasts.**
- **Supplemental figure 22-25: Pseudotime analysis of total-fibroblasts.**
- **Supplemental figure 26-30: GO analysis of total-fibroblasts.**
- **Supplemental figure 31-33: KEGG analysis of total-fibroblasts.**
- **Supplemental figure 34: TSNE of total-ECts.**
- **Supplemental figure 35: Top10 markers of different clusters in ECs.**
- **Supplemental figure 36-37 Pseudotime analysis of total-ECs.**
- **Supplemental figure 38-42: GO analysis of total-ECs.**
- **Supplemental figure 43-45: KEGG analysis of total-ECs.**
- **Supplemental figure 46：A. Dot plot of receptors and ligands analysis. B. Intercellular communication of different celltypes. C. Quantitative figure of intercellular communication.**

Methods:

Analysis and quality control

We analyzed the raw sequencing data by Seurat R package (3.1.1) for single-cell genomics (https://satijalab.org/seurat/). Quality control was performed under these following metrics: (1) gene quantitative quality control (Supplemental table1), using Cell Ranger (3.1.0) which integrated STAR internally and getting the offline data according to the reads comparison to the reference genome (Supplemental figure 2A-B). (2) filter low-quality cells (Supplemental figure 2C), according to their distribution characteristics, fit a distribution model and eliminated outlier value. The linear model fitting curve (Supplemental figure 2C) and the violin chart of the number of genes (nGene, Supplemental figure 2D), the number of the unique molecular identifier (nUMI, Supplemental figure 2E) and the percentage of mitochondrial genes (percent-mito, Supplemental figure 2F) of each cell before and after quality control are shown. (Before-total: 8977, case: 5656, control: 3321, after-total: 7119, case: 4674, control: 2445).

Data dimension reduction and differentially expressed genes

We identified the top variable genes across single cells by using the method described in Macosko et al.[1] Briefly, the average expression and dispersion were calculated for each gene, genes were subsequently placed into several bins based on expression. Principal component analysis (PCA) was

performed to reduce the dimensionality on the log transformed gene-barcode matrices of top variable genes. Cells were clustered based on a graph-based clustering approach, and were visualized in 2-dimension using tSNE. Likelihood ratio test that simultaneously test for changes in mean expression and in the percentage of expressed cells was used to identify significantly differentially expressed genes between clusters. Here, we use the R package SingleR, a novel computational method for unbiased cell type recognition of scRNA-seq, with the reference transcriptomic datasets (https://ftp.ncbi.nlm.nih.gov/blast/db/FASTA/) to infer the cell of origin of each of the single cells independently and identify cell types. Moreover, we screen for differentially expressed genes (DEGs) between case and control groups based on the FindMarkers function of Seurat.[2] In need of statistically significant data, inclusion criteria were meet as fold change (>1.5) and p value (>0.05).

Gene ontology enrichment analysis

After obtaining the differentially expressed genes, we performed Gene ontology (GO) enrichment analysis on these genes, and described their functions (combined with the GO annotation results). Methods of GO function enrichment analysis: using all protein coding genes as the background list, and the differential protein coding gene list as the candidate list filtered from the background list, and using the

hypergeometric distribution test to assess whether the p value is significantly enriched by calculating the representative GO function set in the differential protein coding gene list, and then the p value is corrected by Benjamini & Hochberg multiple tests to obtain qValue.

$$p = 1 - \sum_{i=0}^{m-1} \frac{\binom{M}{i}\binom{N-M}{n-i}}{\binom{N}{n}} \qquad Enrichment\ score = \frac{m}{n} \Big/ \frac{M}{N}$$

N: the number of genes with GO annoyations in all genes; n: the number of genes with GO annotations in the differentially expressed genes in N; M: the number of genes annotated as a specific GO term in all genes; m: the number of differentially expressed genes annotated specifically in GO term.

Kyoto Encyclopedia of Genes and Genomes

Kyoto Encyclopedia of Genes and Genomes (KEGG, https//www.kegg.jp/), a public database connected with pathway, was linked to our differentially expressed genes. We used the KEGG database to perform pathway analysis on the differential protein coding genes (combined with the KEGG annotation results), and used the hypergeometric distribution test to calculate the significance of the enrichment of differential genes in each pathway entry.

Pseudotime analysis

Pseudotime analysis, as known as cell trajectory analysis, help us know the sequence of regulatory changes that occur when cells transition from one state to another. We used the Monocle 2 software package[3] to perform machine learning based on the expression patterns of key genes, and then simulate the dynamic changes of the time development process. First, we selected genes with a large degree of gene expression variation between cells, and performed spatial dimensionality reduction based on their expression profiles, and then constructed a minimum spanning tree (MST), and then used the MST to find the longest path that represents differentiation trajectory of cells with similar transcription characteristics.

Ligand-receptor networks

According to the ligand-receptor relationship between different cell membrane surfaces and free proteins, possible interactions between different cells can be identified. This effect includes autocrine and paracrine. Utilizing single-cell RNA sequencing data, taking the gene expression data of cell subpopulations as the research object, with the aid of the ligand-receptor database, using cellphoneDB software can obtain the ligand and receptor information in the cell, and obtain the signal communication relationship between cells help us clarify the complexity, diversity and dynamics of cell-to-cell communication in a wide range of

biological processes.

Cell culture

Mouse aortic vascular smooth muscle cells (MOVAS) were obtained from American Type Culture Collection (ATCC, CRL-2797). MOVAS were cultured in Smooth Muscle Cell Growth Basal Medium (ScienCell, 1101) with 2% fetal bovine serum (FBS) at 37 °C with 95% humid air and 5% CO_2. After growing to 70%-80% confluence, cells were washed with PBS and then maintained in Smooth Muscle Cell Growth Basal Medium.

Immunofluorescence

Sections of rat carotid arteries or MOVAS were fixed in 4% paraformaldehyde in PBS for 10 minutes and permeabilized with 0.1% Triton X-100 (in PBS) for 20 minutes. After blocking with 3% BSA for 1 hour, samples were incubated with antibodies specific for NMT1 (ab226857, 1:100; Abcam), α-SMA (ab5694, 1:100; Abcam) overnight at 4°C. These samples were washed by PBS and labeled by FITC (ab6785, 1:100; Abcam) for 1 hour at room temperature. Finally, mounted them by DAPI (ab104139; Abcam). Finally, images were obtained by a laser scanning confocal microscope (ZEISS, German).

References

1. Macosko EZ, Basu A, Satija R, Nemesh J, Shekhar K, Goldman M, Tirosh I, Bialas AR, Kamitaki

N, Martersteck EM, Trombetta JJ, Weitz DA, Sanes JR, Shalek AK, Regev A and McCarroll SA. Highly Parallel Genome-wide Expression Profiling of Individual Cells Using Nanoliter Droplets. *Cell*. 2015;161:1202-1214.

2. Butler A, Hoffman P, Smibert P, Papalexi E and Satija R. Integrating single-cell transcriptomic data across different conditions, technologies, and species. *Nature biotechnology*. 2018;36:411-420.

3. Trapnell C, Cacchiarelli D, Grimsby J, Pokharel P, Li S, Morse M, Lennon NJ, Livak KJ, Mikkelsen TS and Rinn JL. The dynamics and regulators of cell fate decisions are revealed by pseudotemporal ordering of single cells. *Nature biotechnology*. 2014;32:381-386.

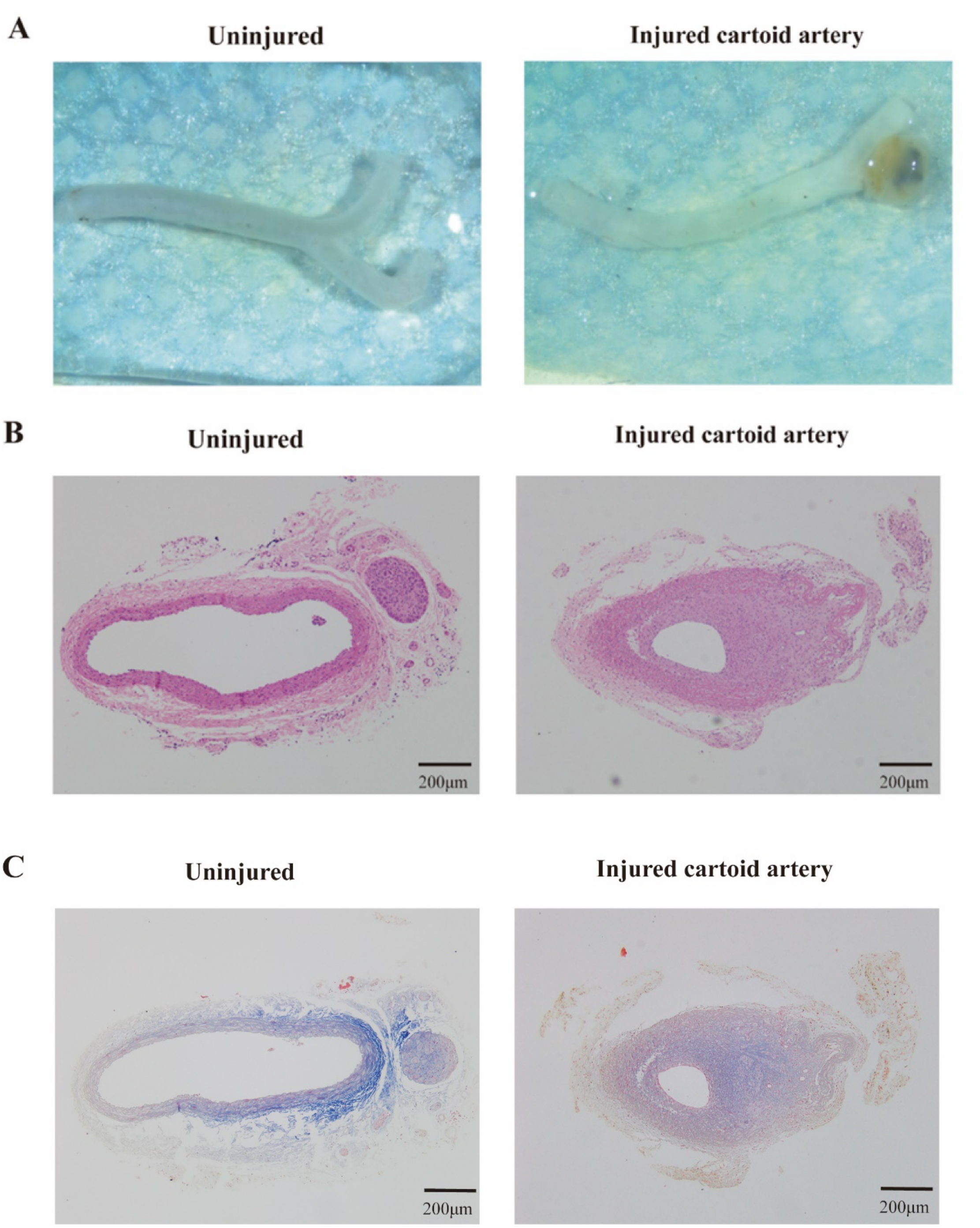

Supplemental figure 1. Cartoid arteries of an experimrntal model. The brightfield, HE and Masson's trichrome staining indicate successful model building.

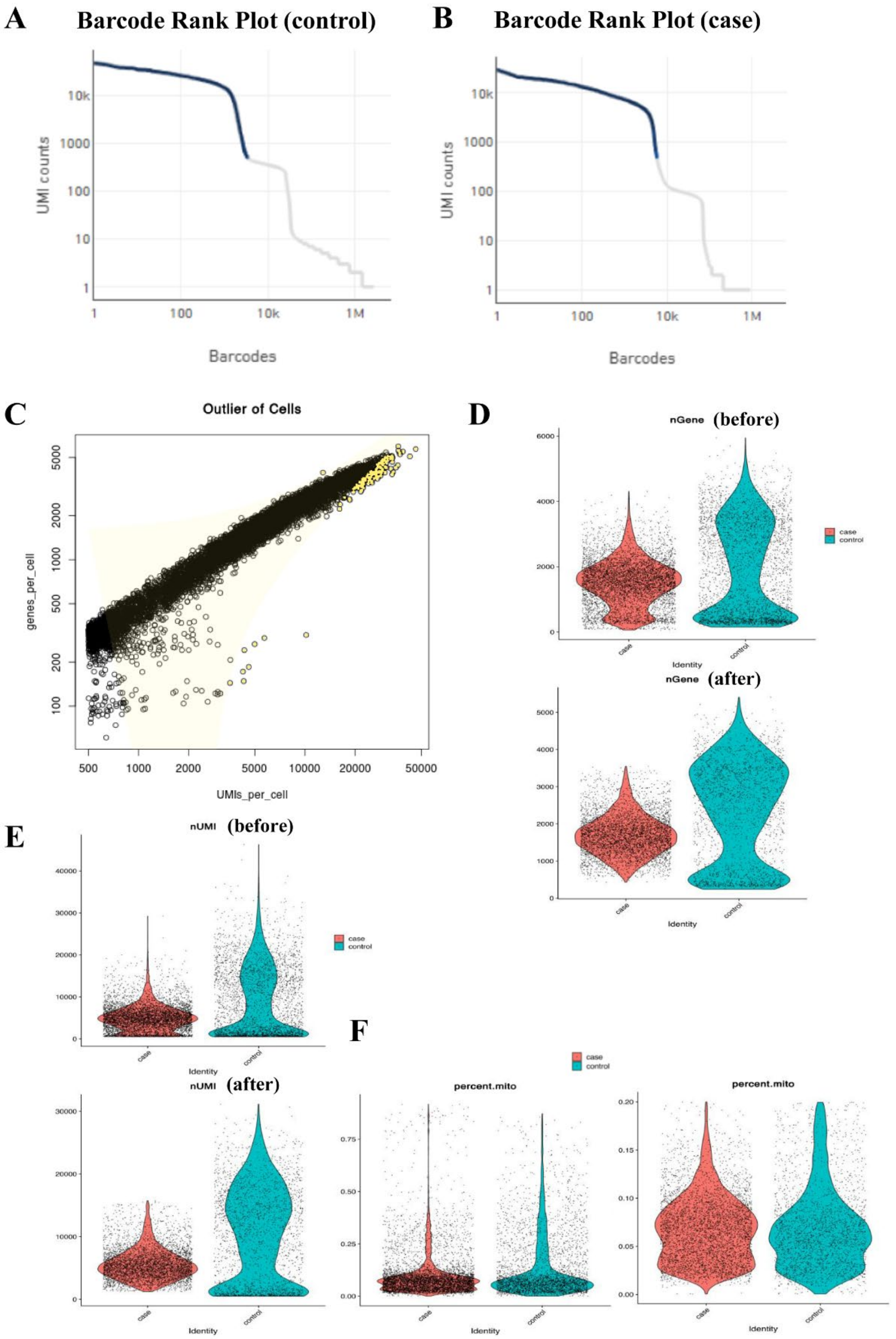

Supplemental figure 2: Quality control of single-cell database. The quality control shows a great building of single-cell database.

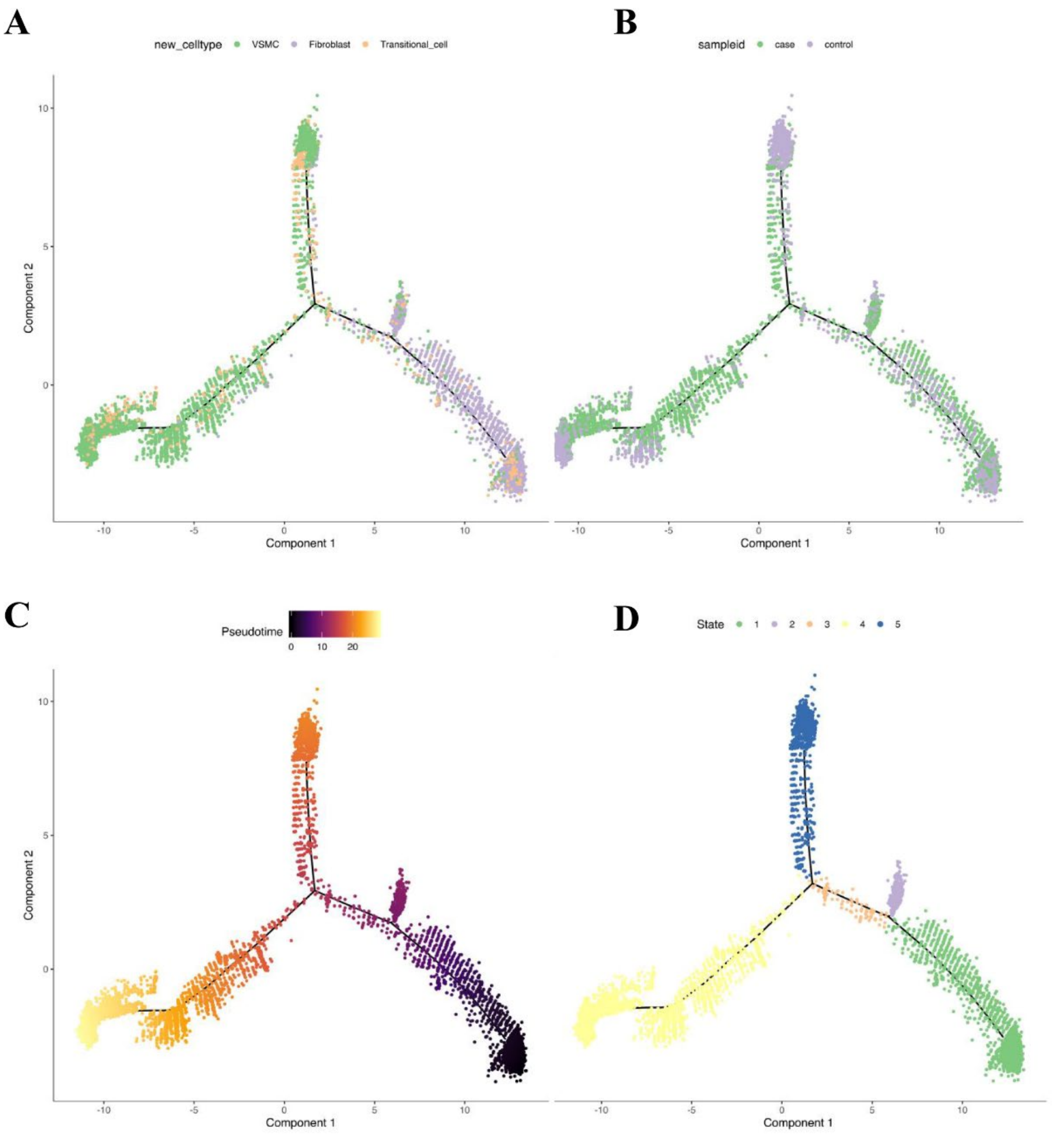

Supplemental figure 3. Pseudotime analysis of VSMCs, fibroblasts and transitional-cell in total group. The transitional-cells in both case and control groups also distribute in the routine between VSMC and fibroblast.

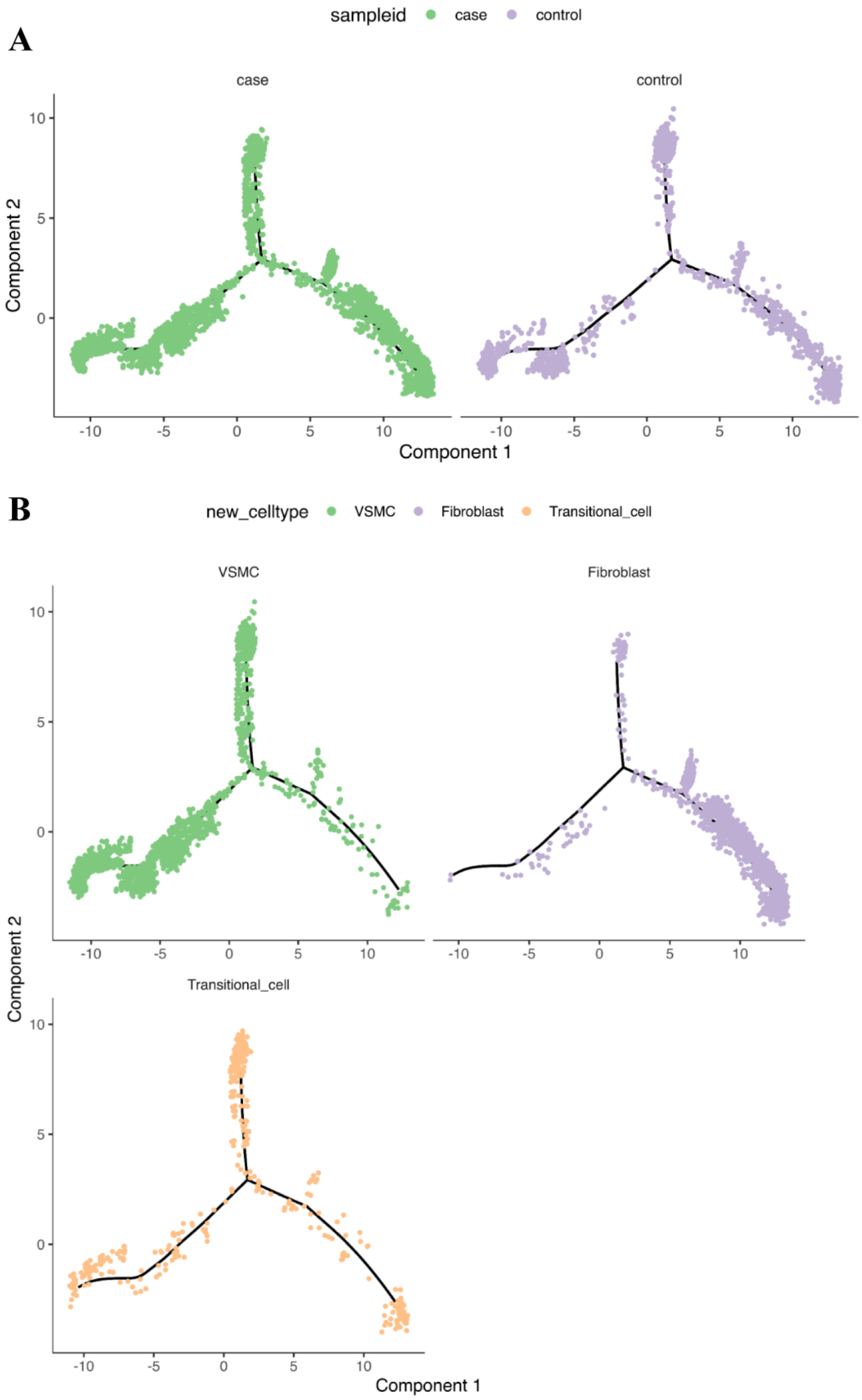

Supplemental figure 4. Pseudotime analysis of VSMCs, fibroblasts and transitional-cell in total group. We split transitional-cells and found their transitional function between VSMCs and fibroblasts.

Fibroblast (control)

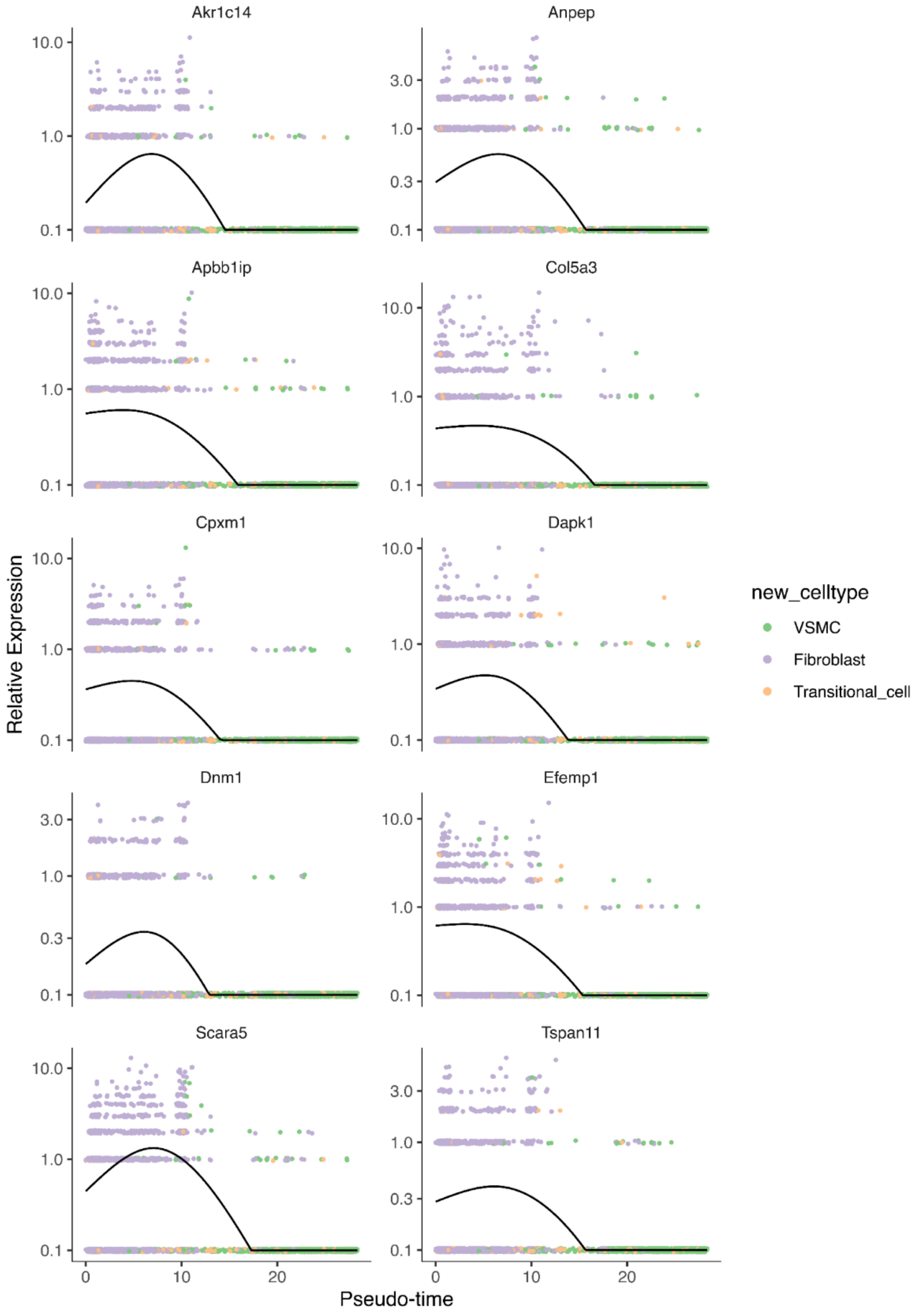

Supplemental figure 5: Pseudotime analysis of top10 differentially expressed genes of fibroblasts in control group.

Fibroblast (case)

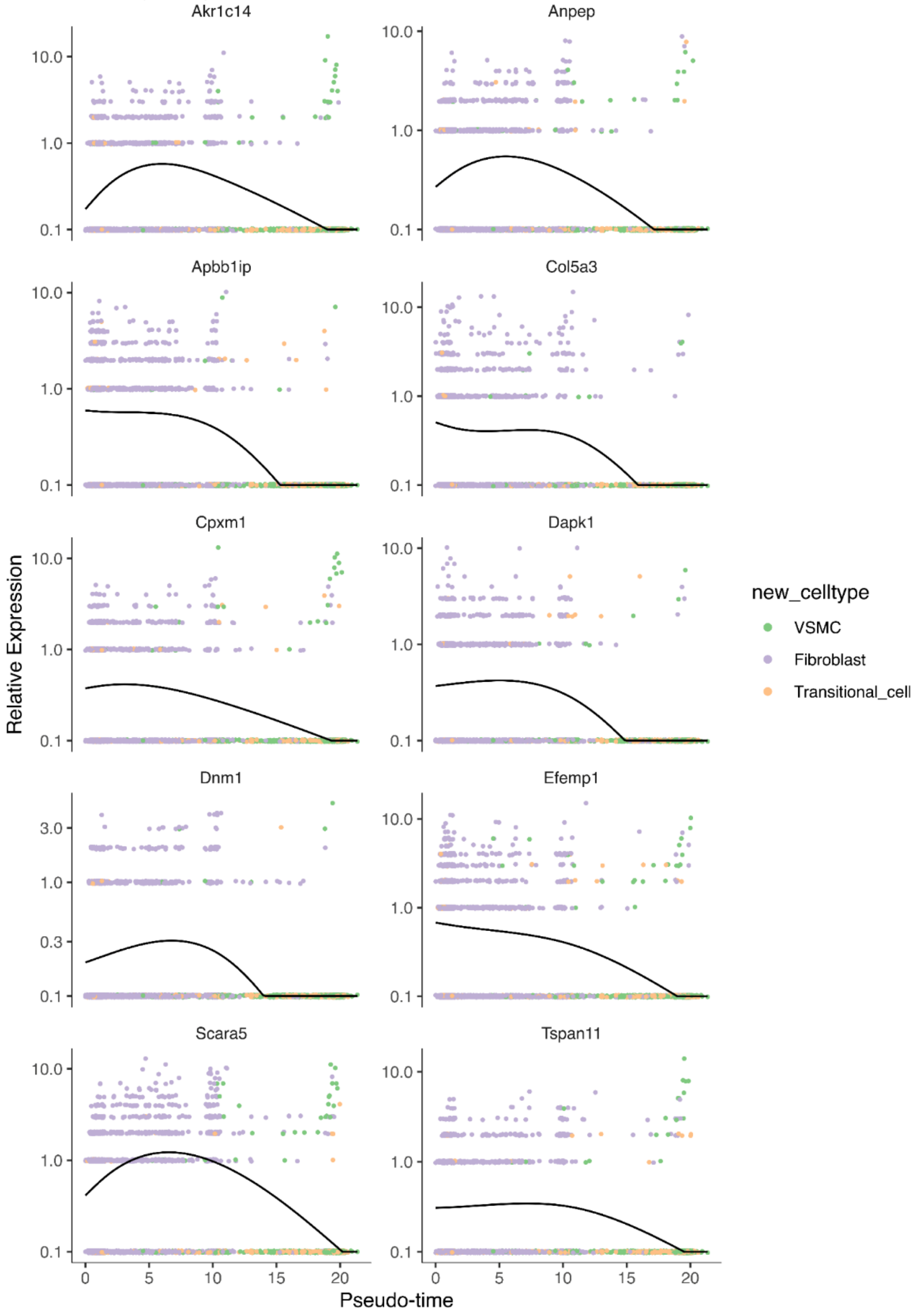

Transitional-cell (control)

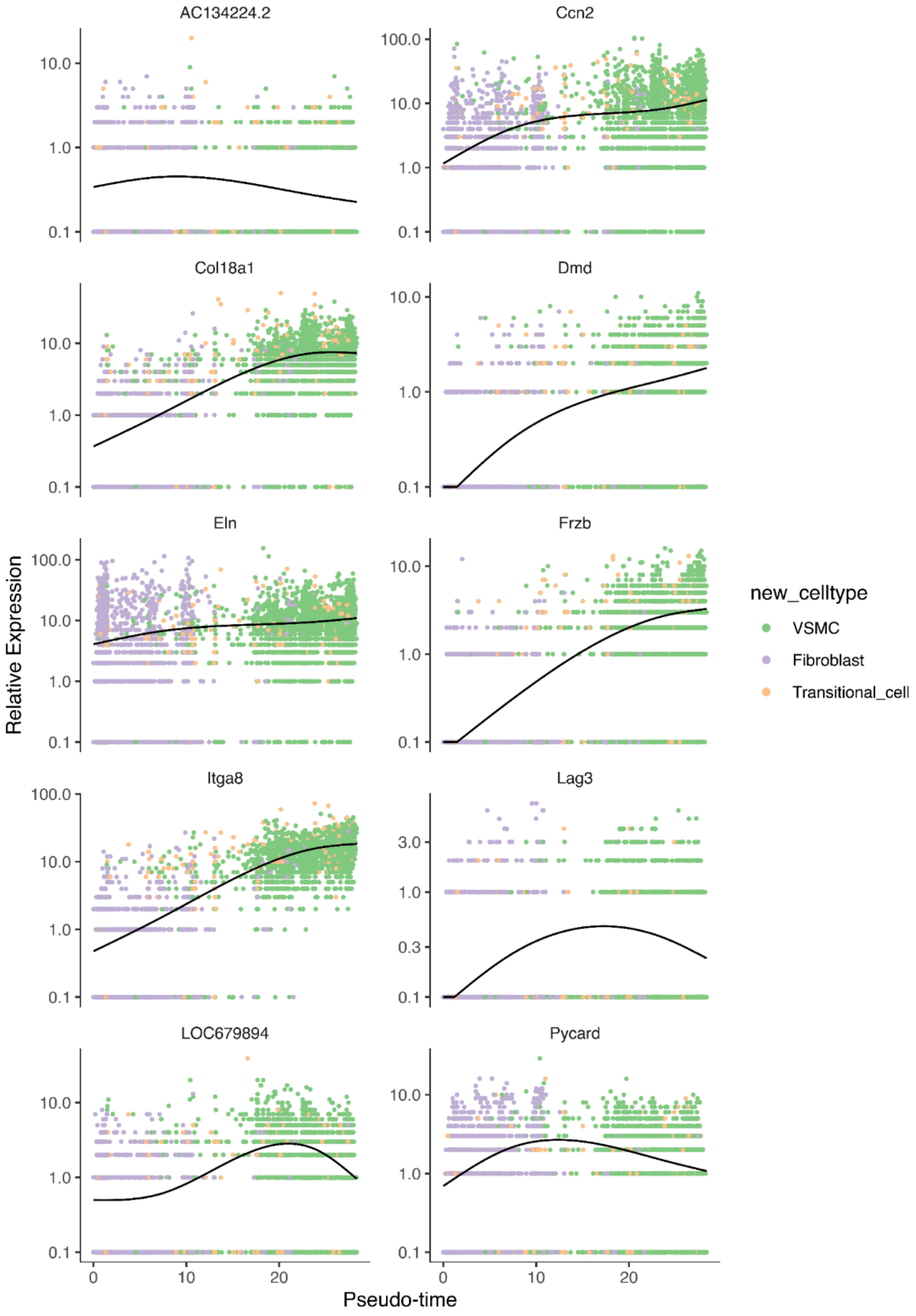

Supplemental figure 7: Pseudotime analysis of top10 differentially expressed genes of transitional-cells in control group.

Transitional-cell (case)

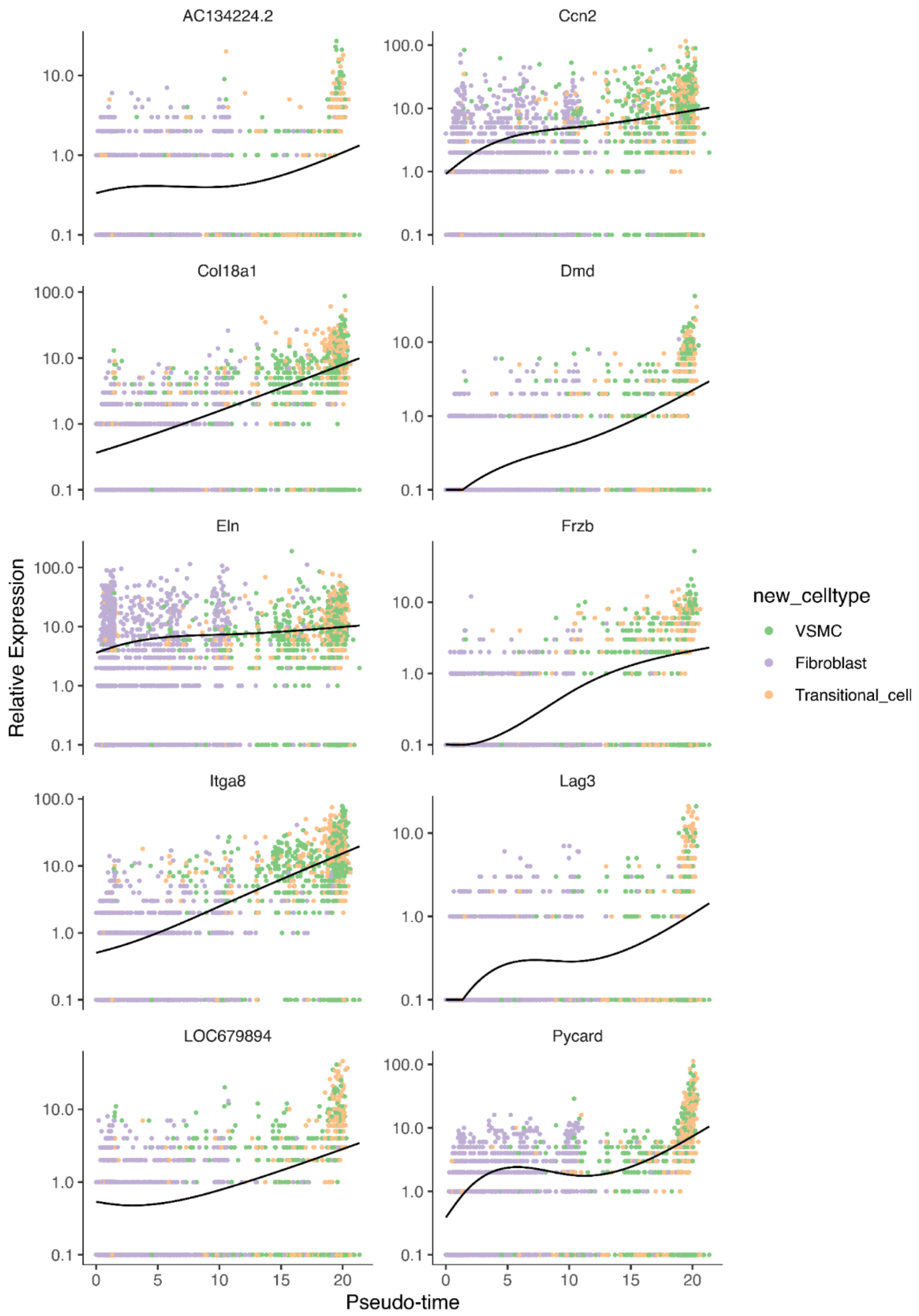

Supplemental figure 8: Pseudotime analysis of top10 differentially expressed genes of transitional-cells in case group.

VSMC (control)

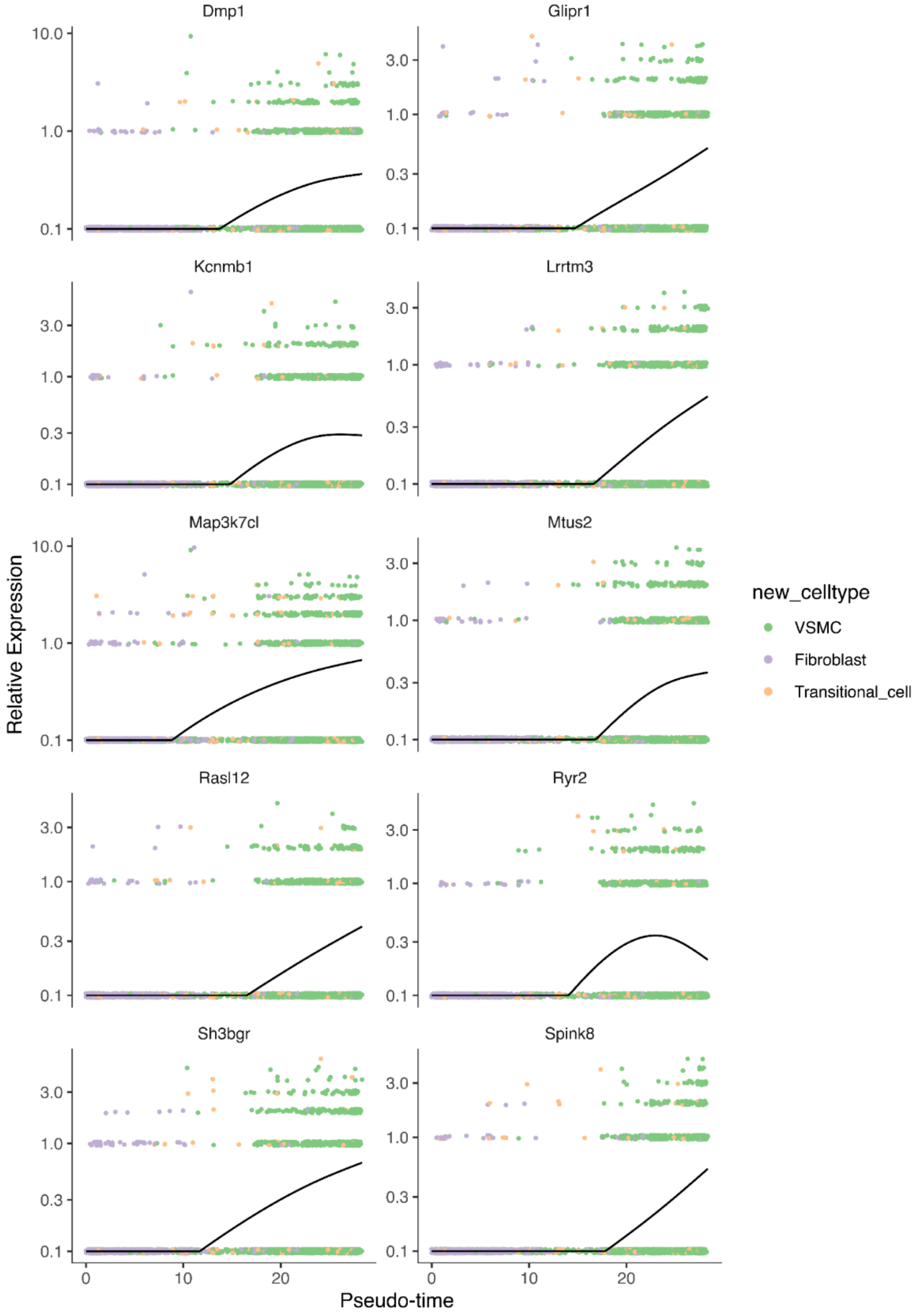

Supplemental figure 9: Pseudotime analysis of top10 differentially expressed genes of VSMCs in control group.

VSMC (case)

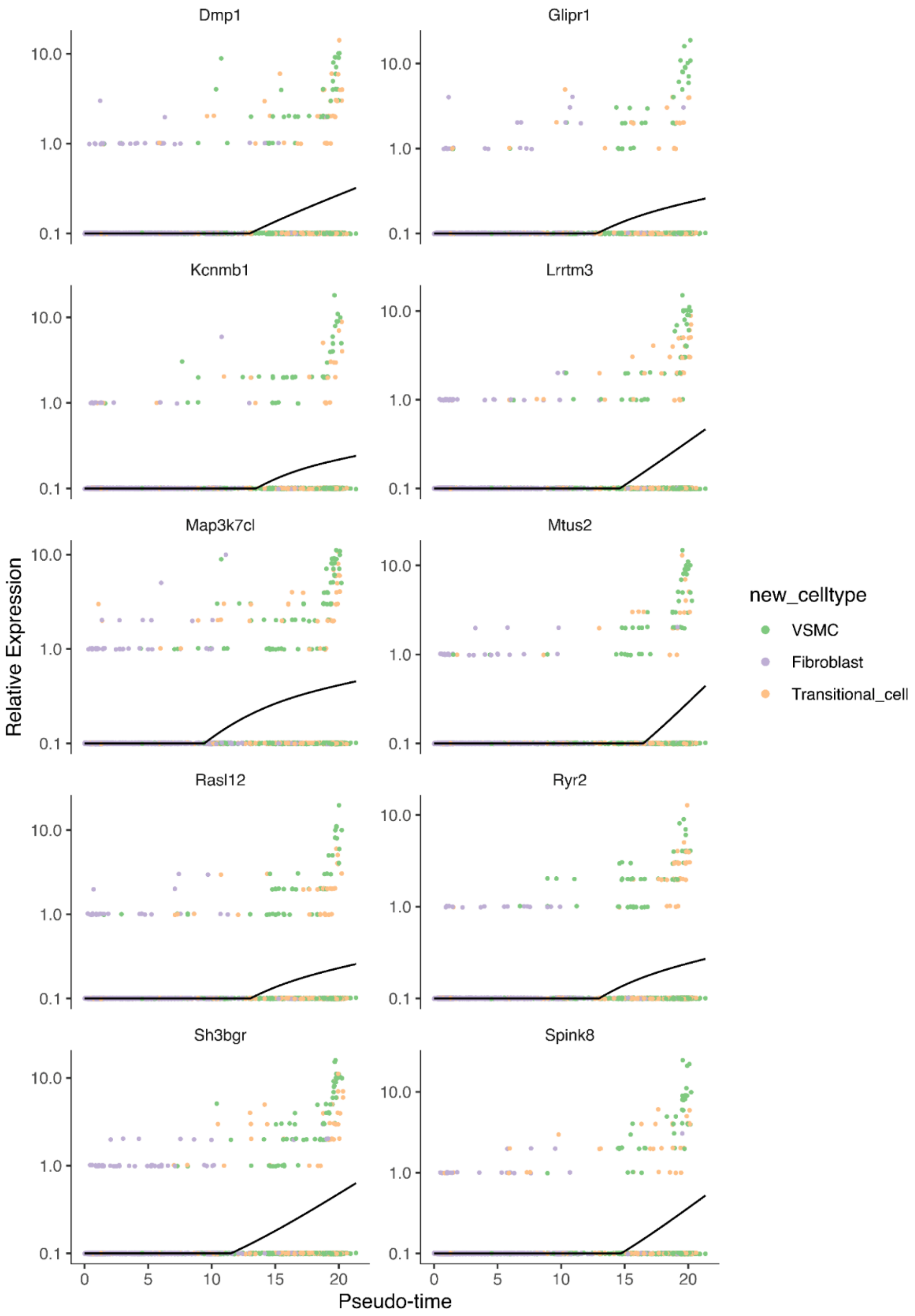

Dmp1
Glipr1
Kcnmb1
Lrrtm3
Map3k7cl
Mtus2
Rasl12
Ryr2
Sh3bgr
Spink8
10.0
1.0
0.1
0
5
10
15
20
Relative Expression
Pseudo-time
new_celltype
VSMC
Fibroblast
Transitional_cell

A

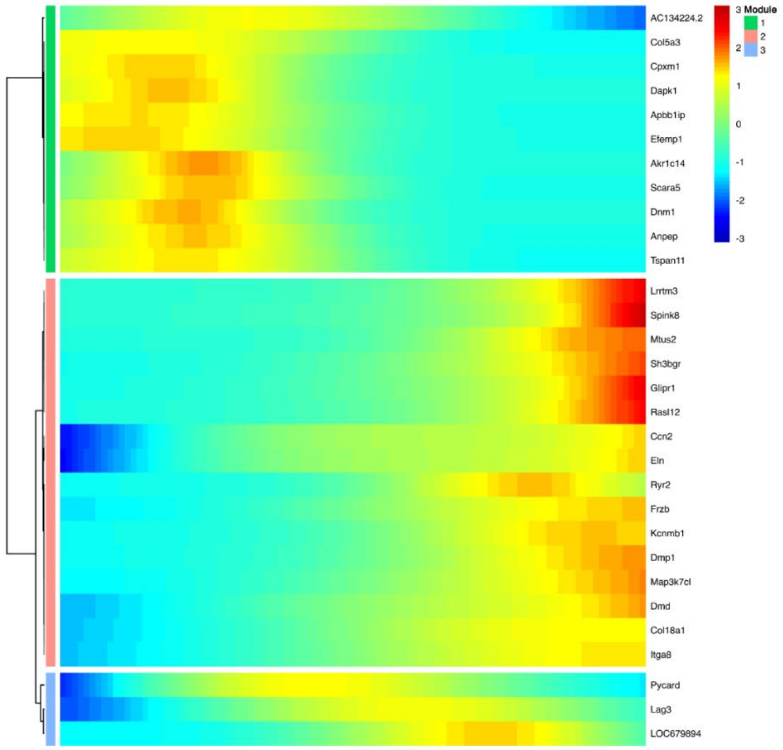

B

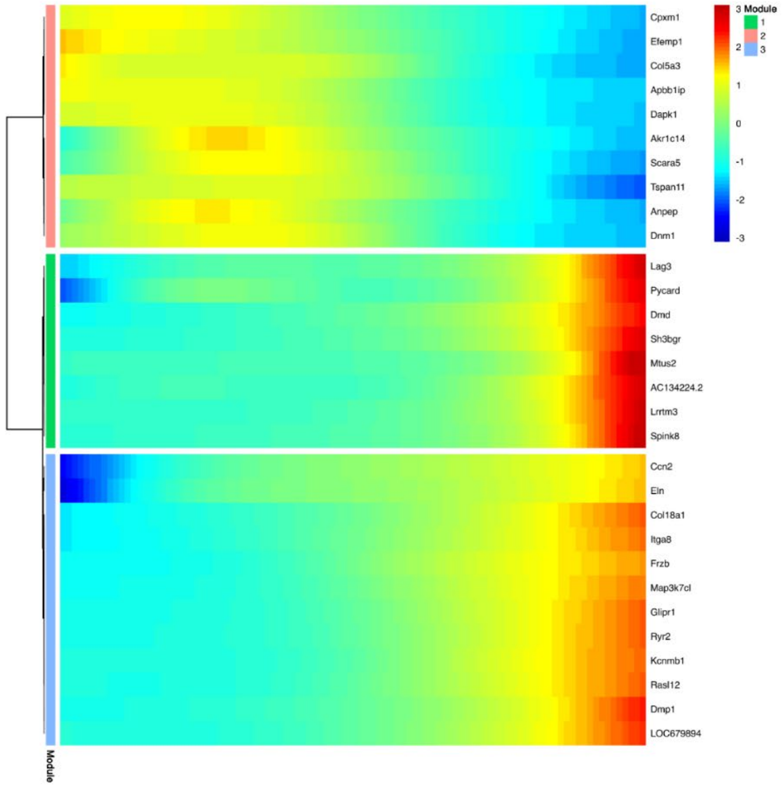

Supplemental figure 11: Hotmap of top10 differentially expressed genes of fibroblasts, transitional-cells and VSMCs. Module1: fibroblasts; Module2: transitional-cells, Module3: VSMCs. Figure A: control group; Figure B: case group.

A

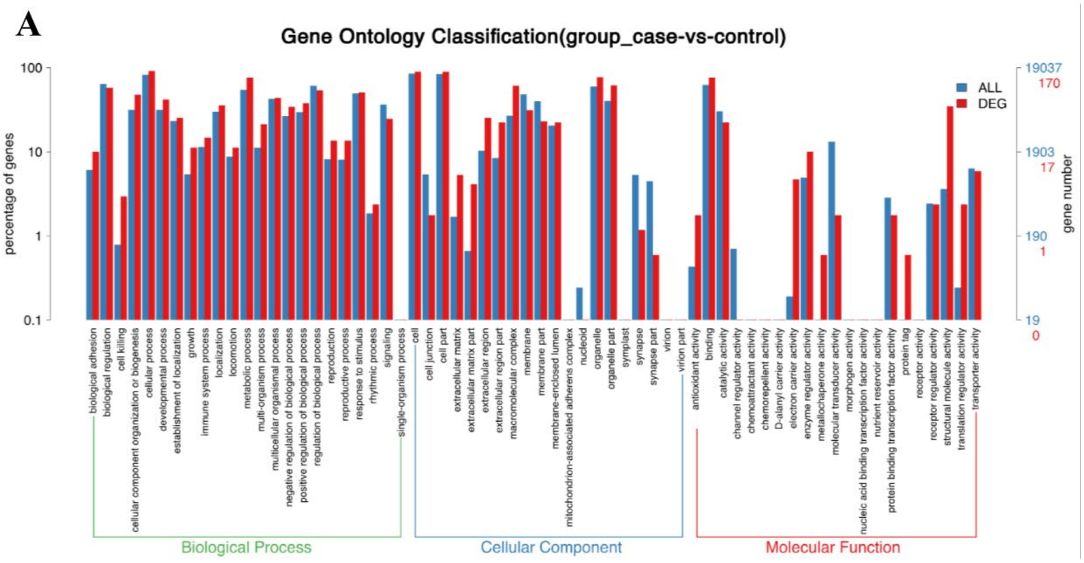

B

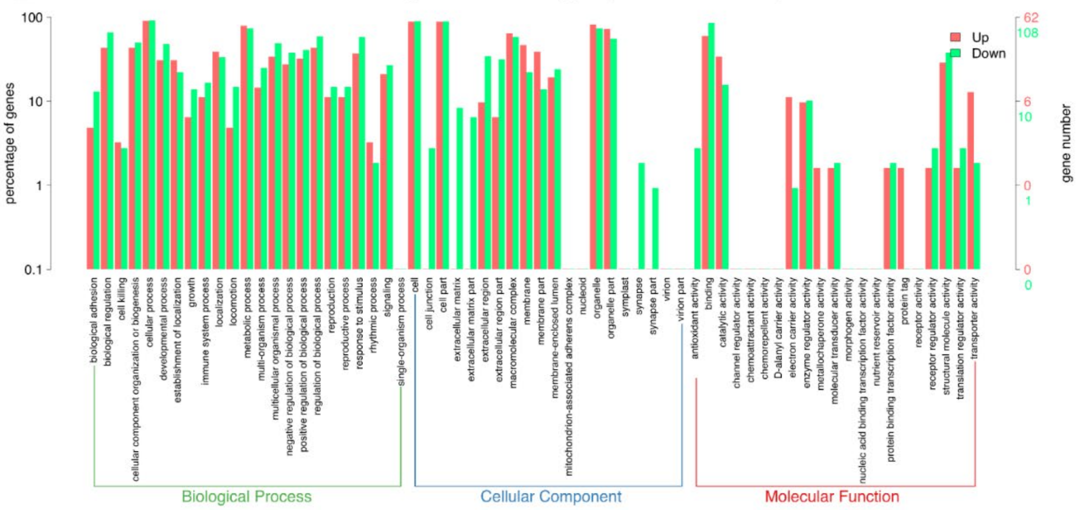

Supplemental figure 12. GO analysis of total-VSMCs. Figure A: GO classification of differentially expressed genes between case and control groups. Figure B: GO classification of highly expressed or lowly expressed genes between case and control groups.

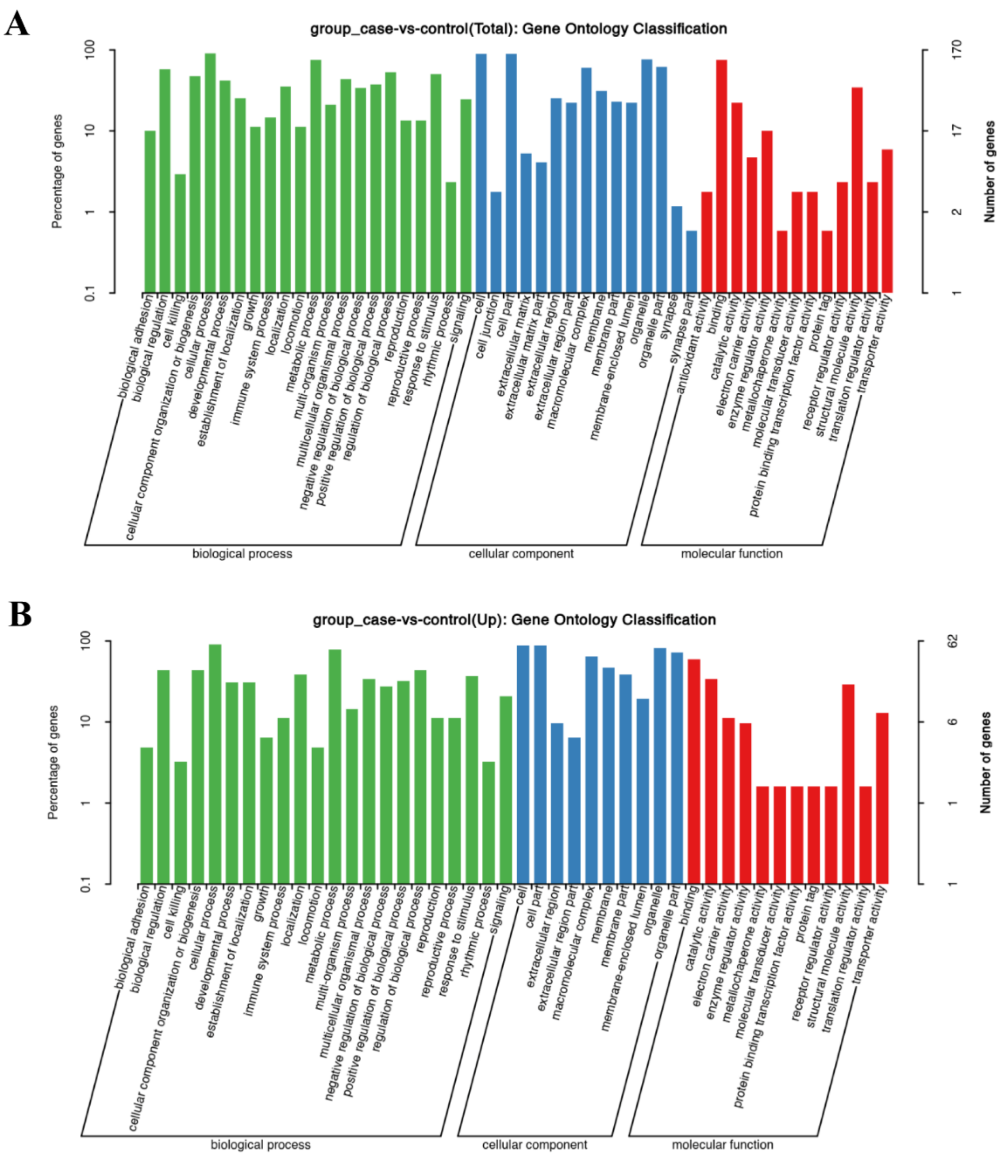

Supplemental figure 13. GO analysis of total-VSMCs. Figure A: Gene ontology classification of differentially expressed genes between case and control groups. Figure B: Gene ontology classification of highly expressed genes between case and control groups.

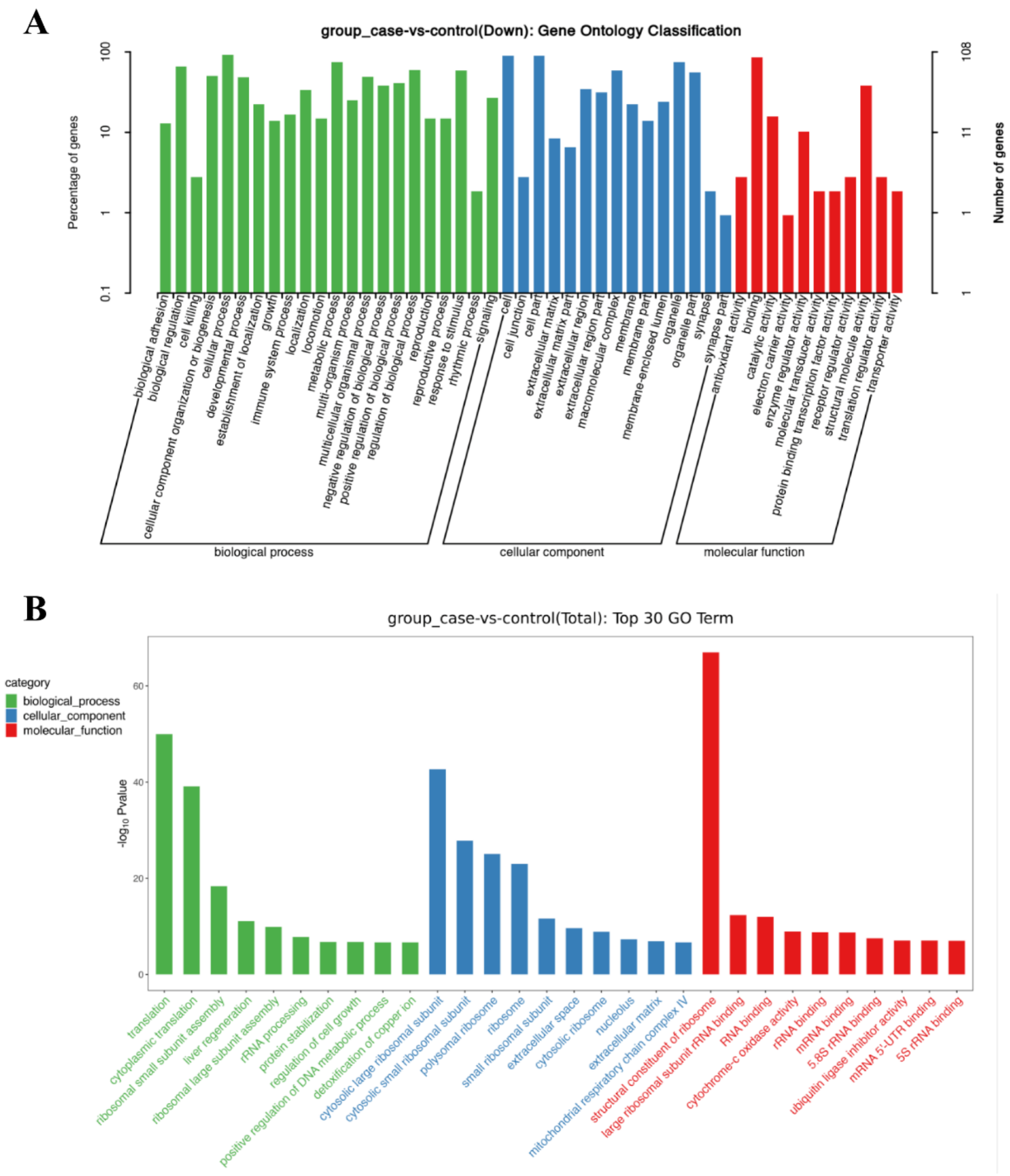

Supplemental figure 14. GO analysis of total-VSMCs. Figure A: Gene ontology classification of lowly expressed genes between case and control groups. Figure B: Top30 GO term of total genes of both case and control group.

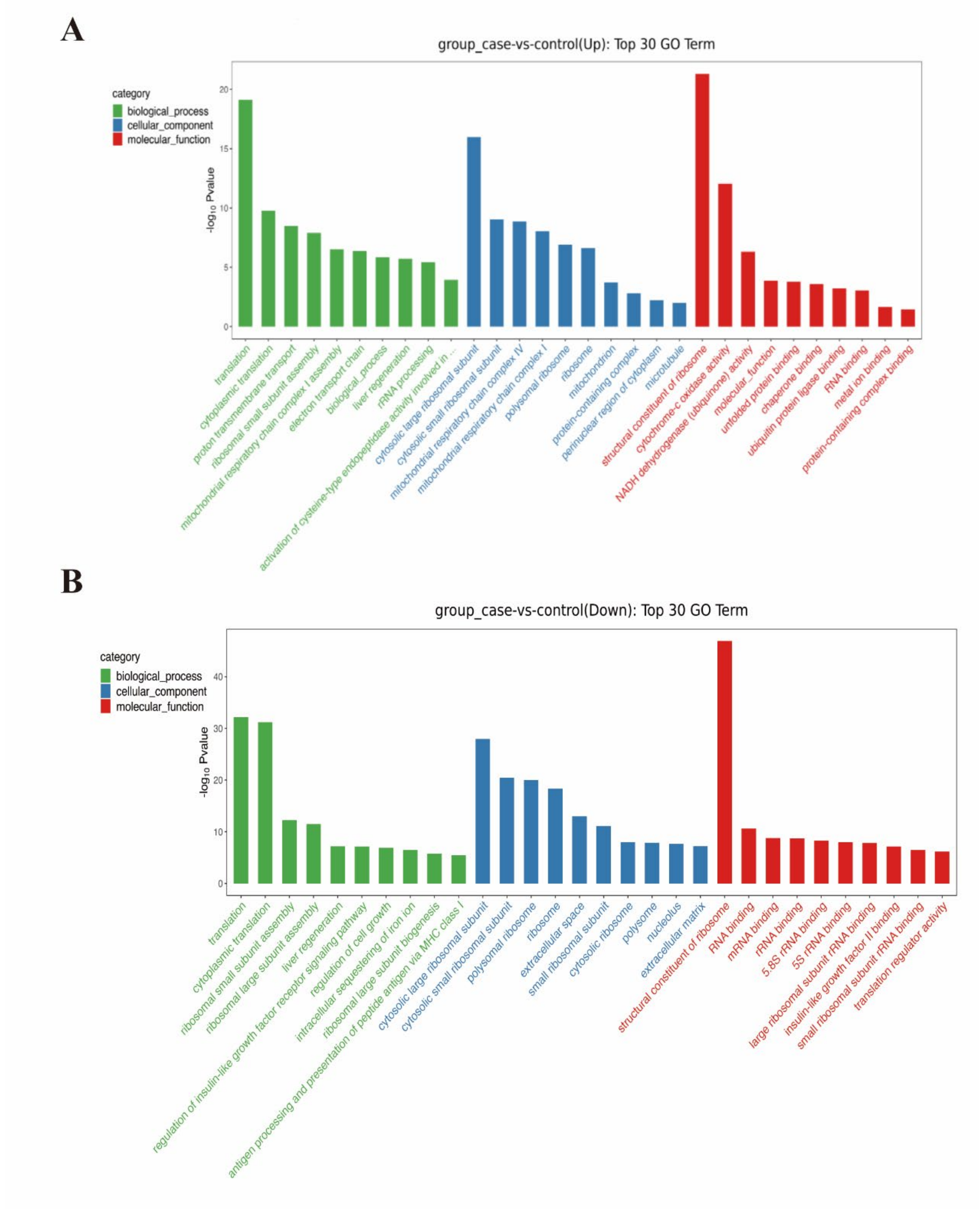

Supplemental figure 15. GO analysis of total-VSMCs. Figure A: Top30 GO term of highly expressed genes between case and control group. Figure B: Top30 GO term of lowly expressed genes between case and control group.

A

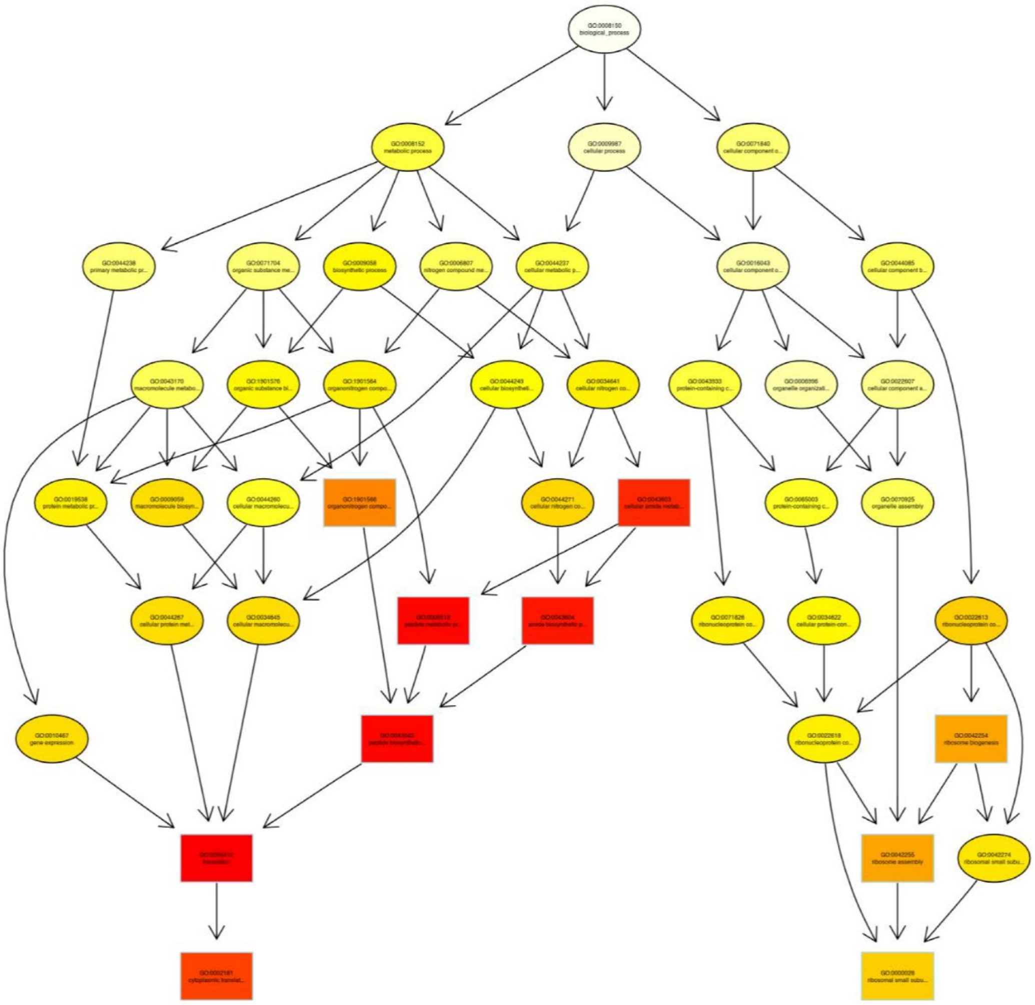

Supplemental figure 16. GO analysis of total-VSMCs. The communication network of GO terms.

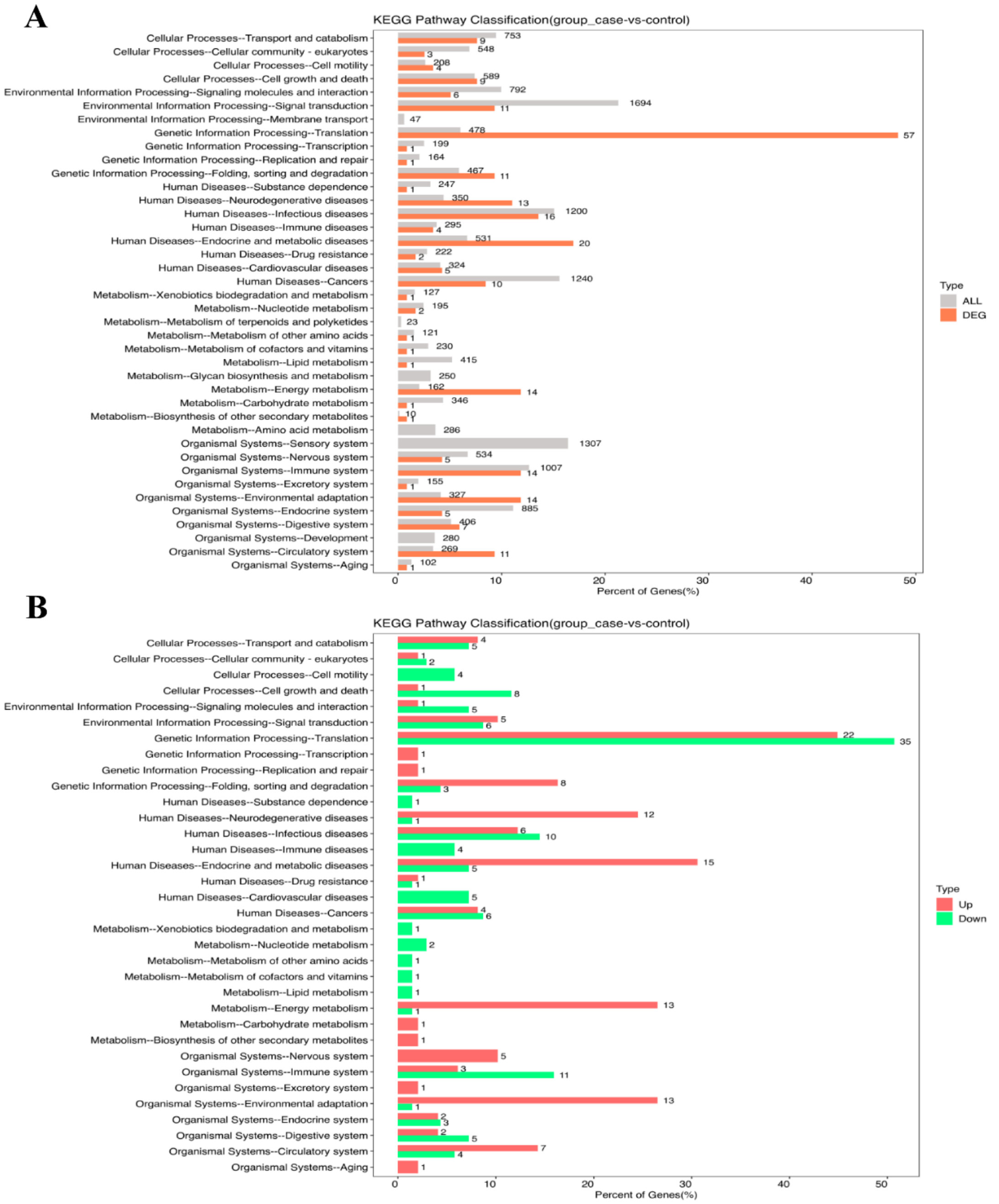

Supplemental figure 17. KEGG analysis of total-VSMCs. Figure A: KEGG pathway classification of differentially expressed genes between case and control groups. Figure B: KEGG pathway classification of highly expressed or lowly expressed genes between case and control groups.

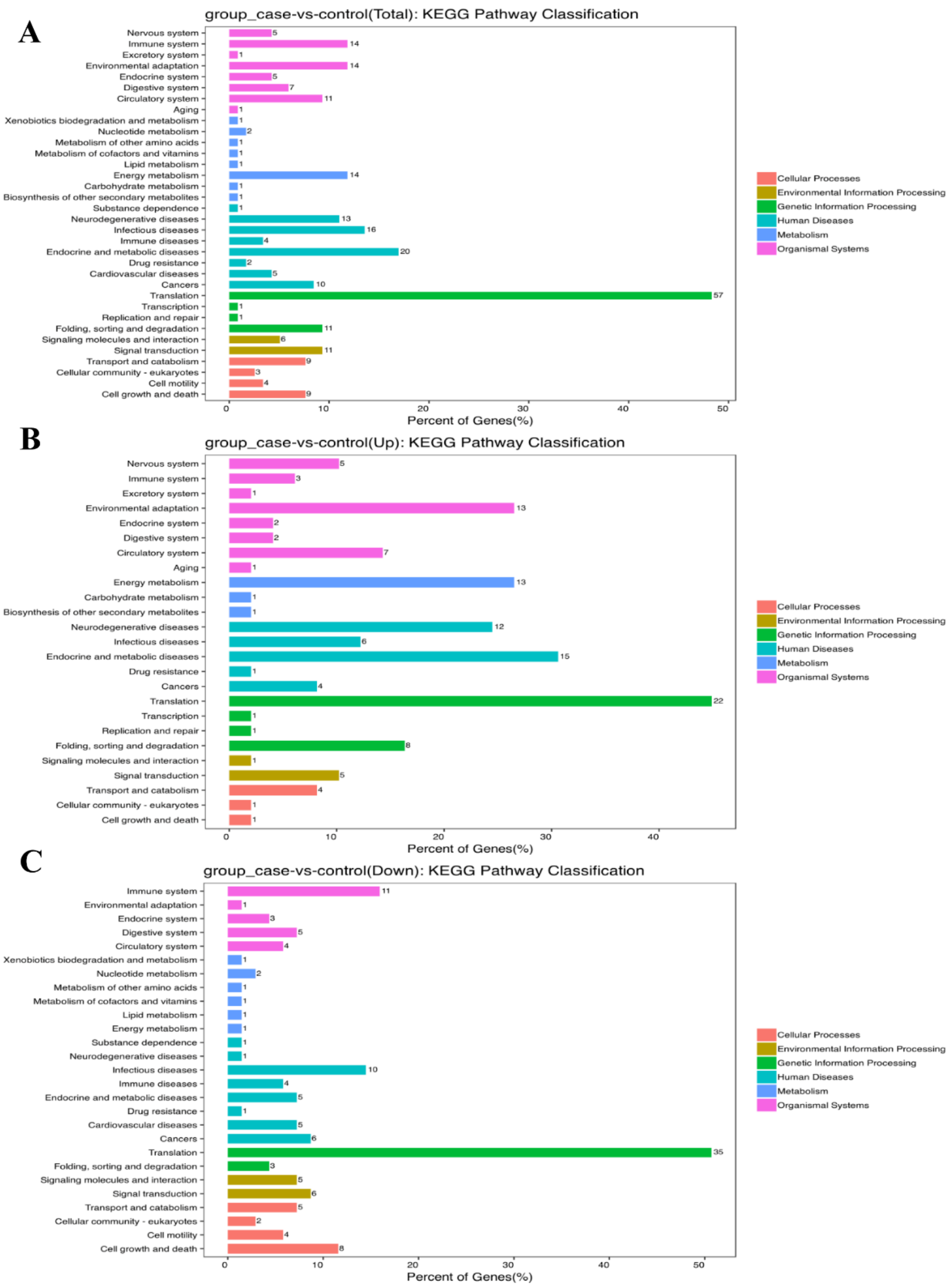

Supplemental figure 18. KEGG analysis of total-VSMCs. Figure A: KEGG pathway classification of total genes of both case and control group. Figure B: KEGG pathway classification of highly expressed genes between case and control groups. Figure C: KEGG pathway classification of lowly expressed genes between case and control groups.

A

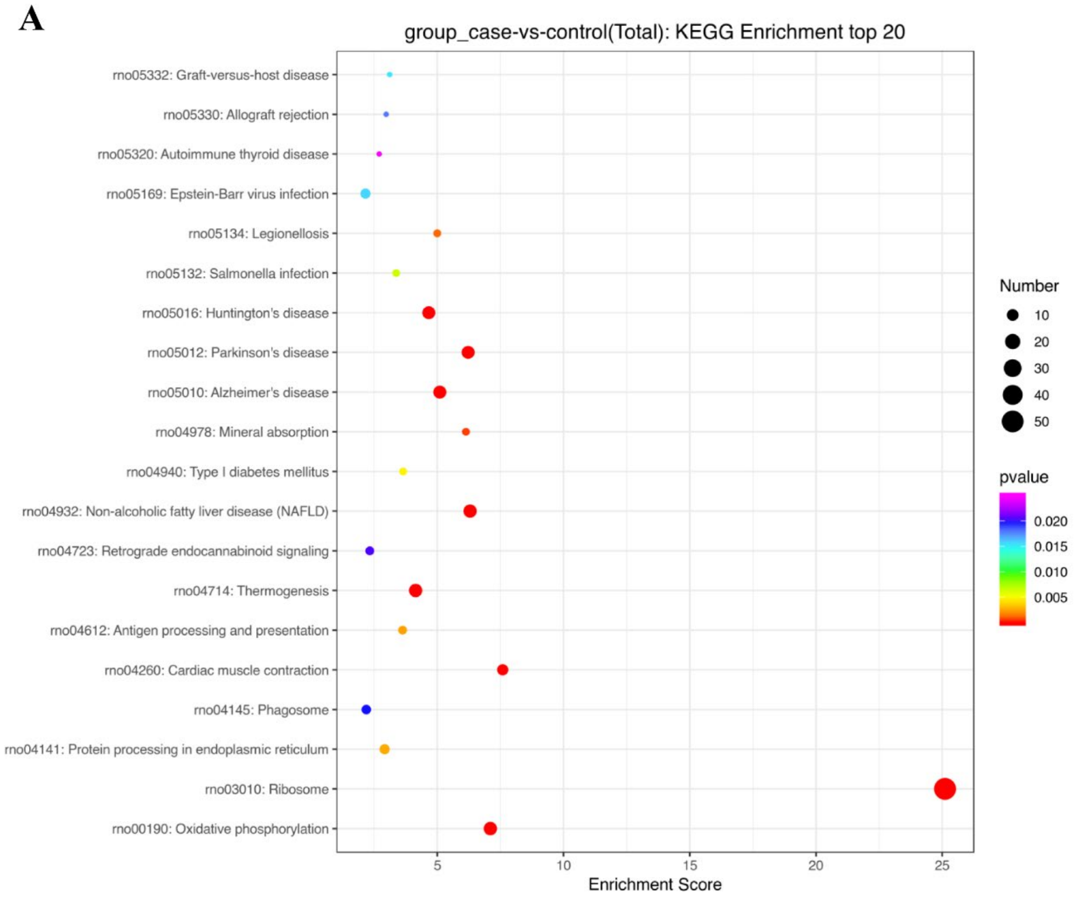

Supplemental figure 19. KEGG analysis of total-VSMCs. KEGG enrichment top20 of total genes in both case and control group.

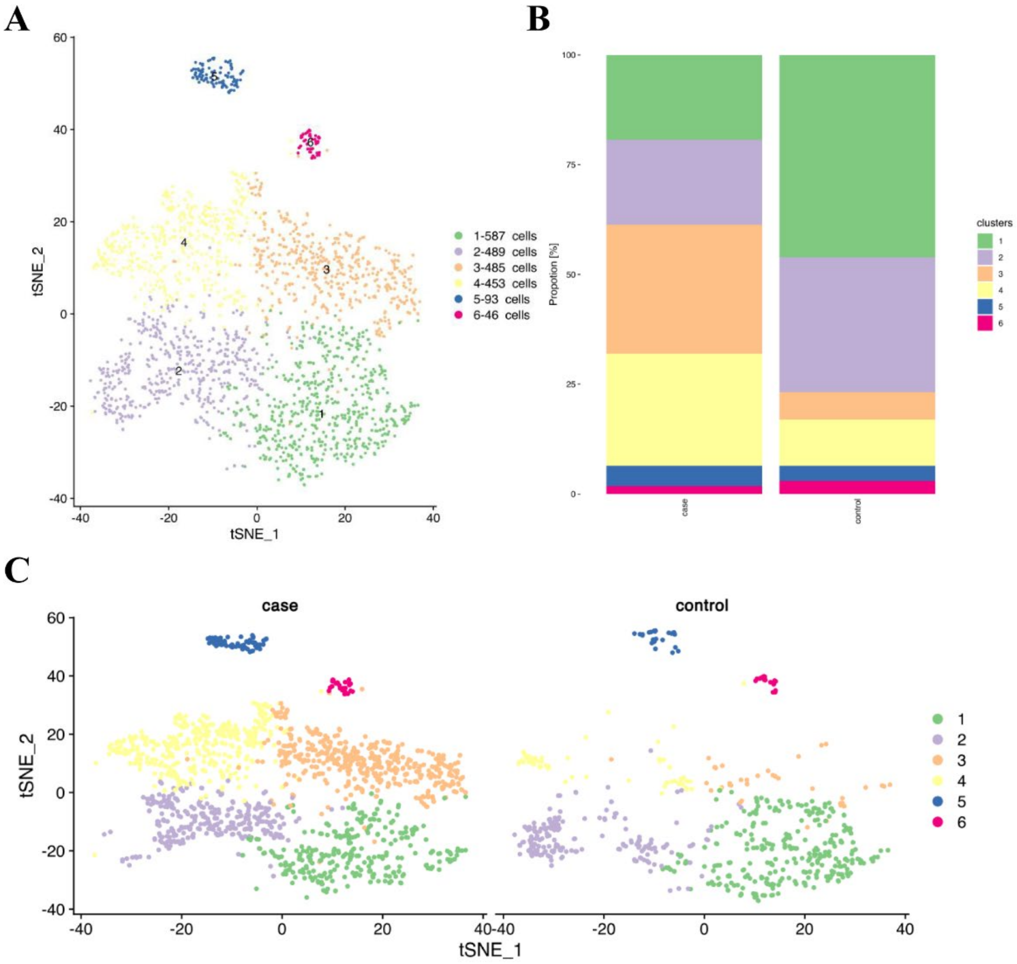

Supplemental figure 20. TSNE of total-fibroblasts. Figure A: 6 clusters of total-fibroblasts. Figure B: Proportion of 6 different clusters. Figure C: TSNE of 6 clusters in case and control groups.

A

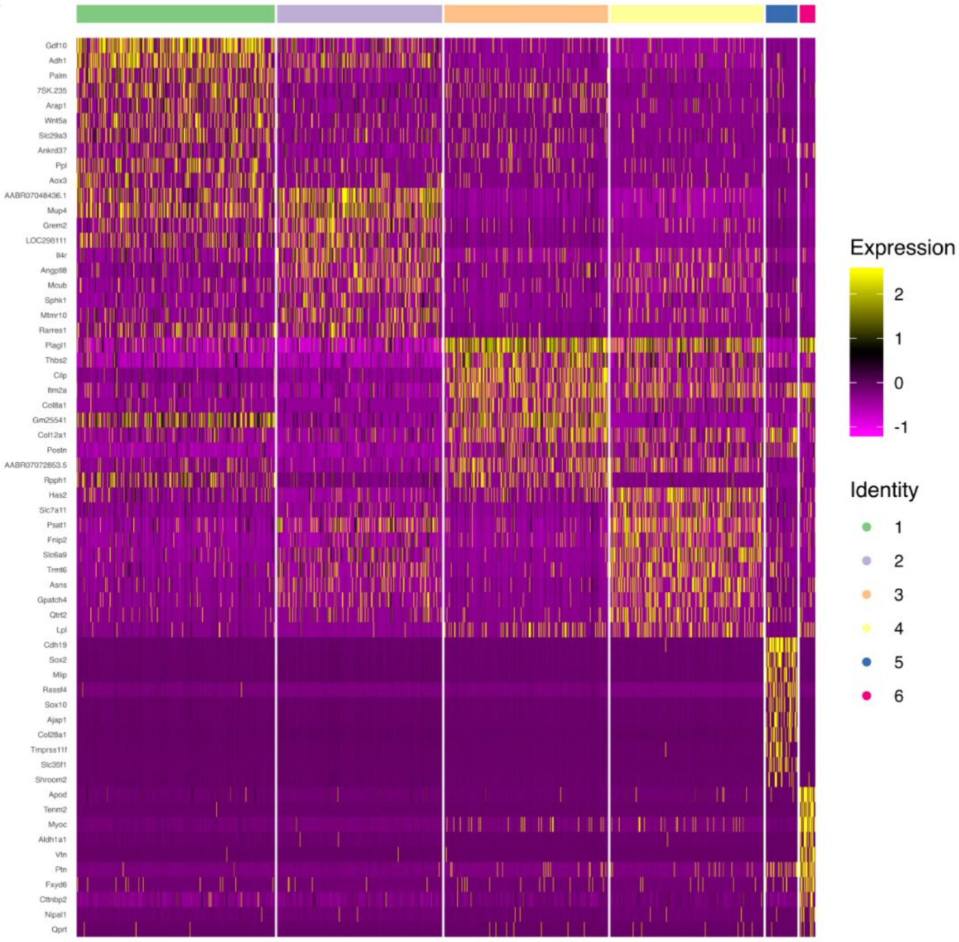

Supplemental figure 21. Top10 markers of different clusters in fibroblasts.

A

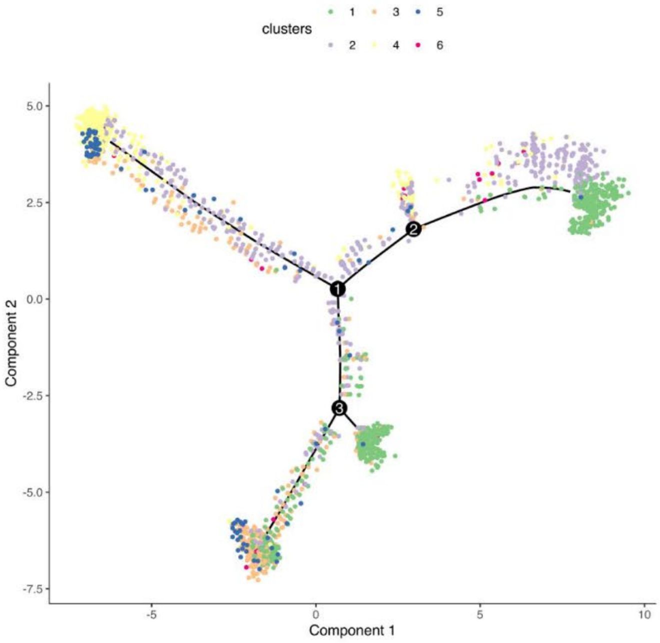

B

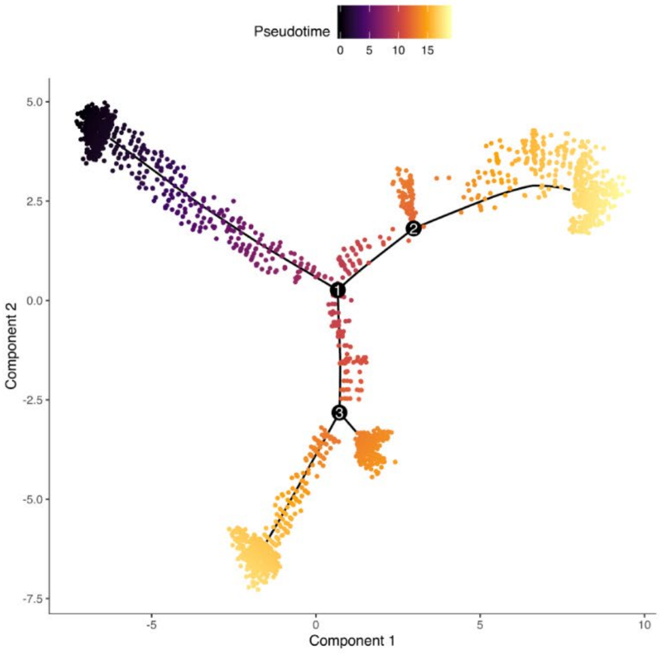

Supplemental figure 22. Pseudotime analysis of total-fibroblasts.

A

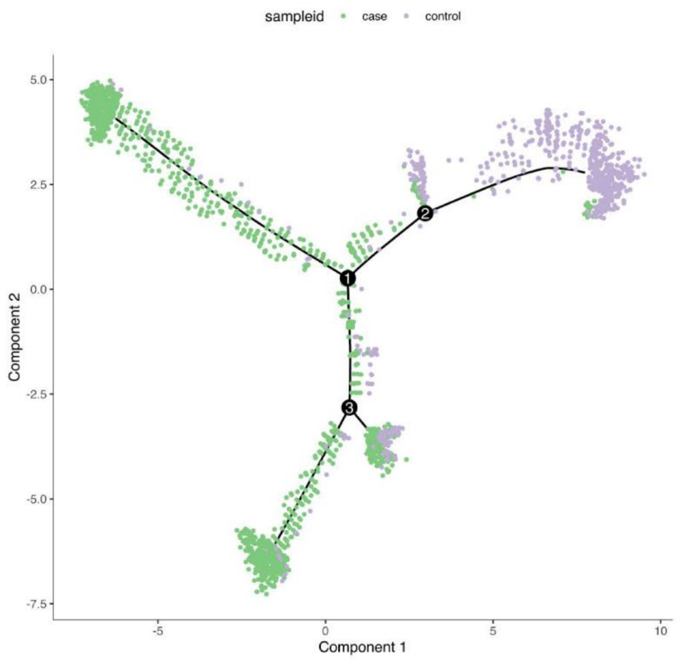

B

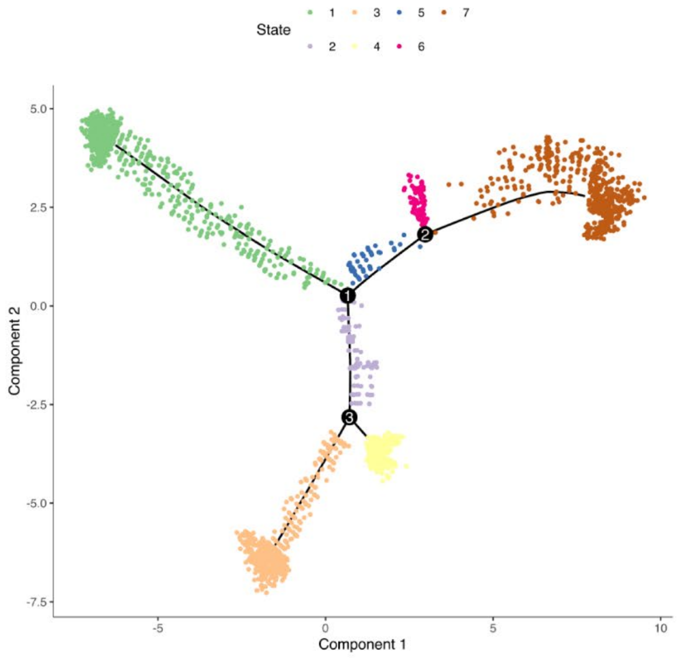

Supplemental figure 23. Pseudotime analysis of total-fibroblasts.

A

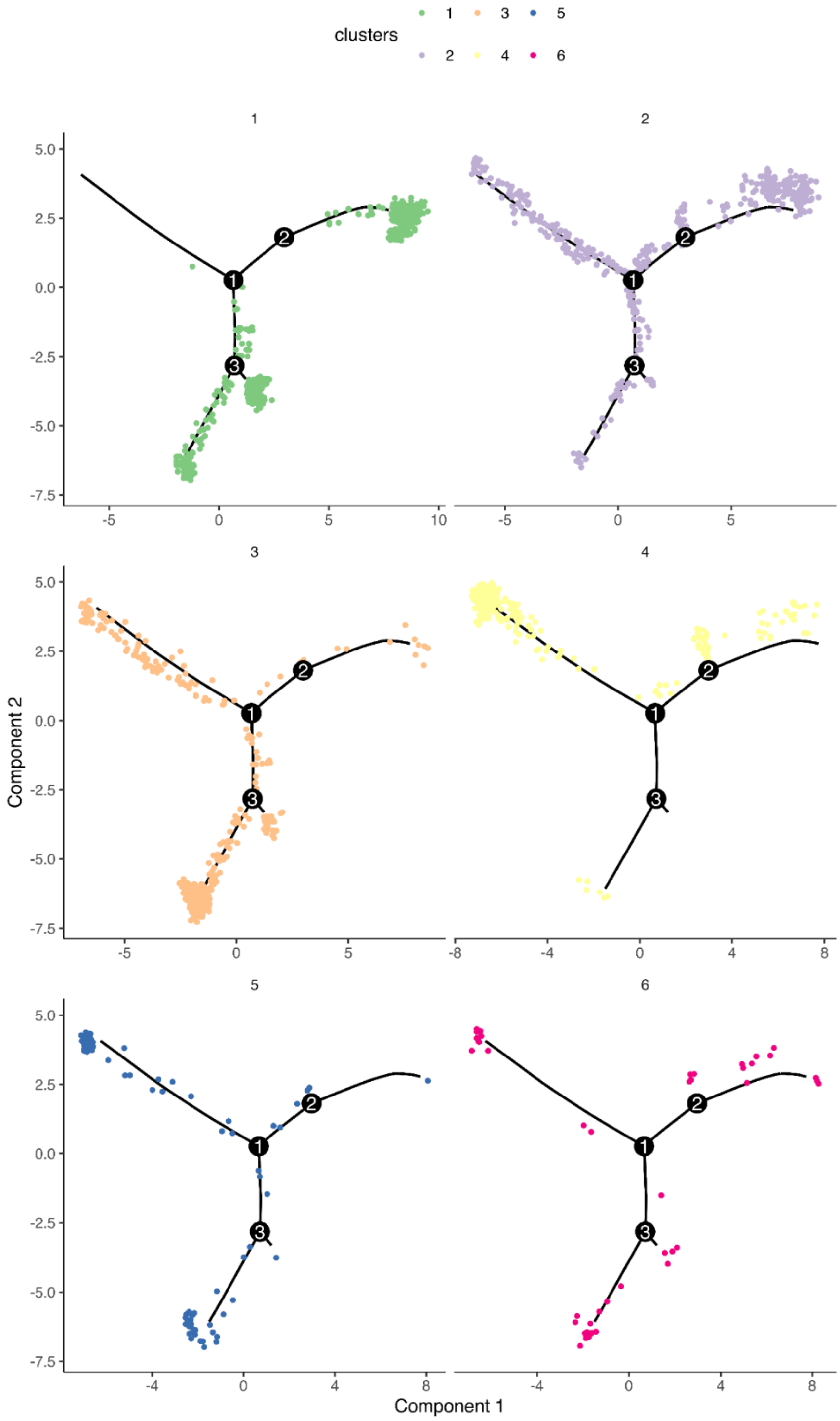

Supplemental figure 24. Pseudotime analysis of total-fibroblasts. Split of 6 clusters in pseudotime analysis.

A

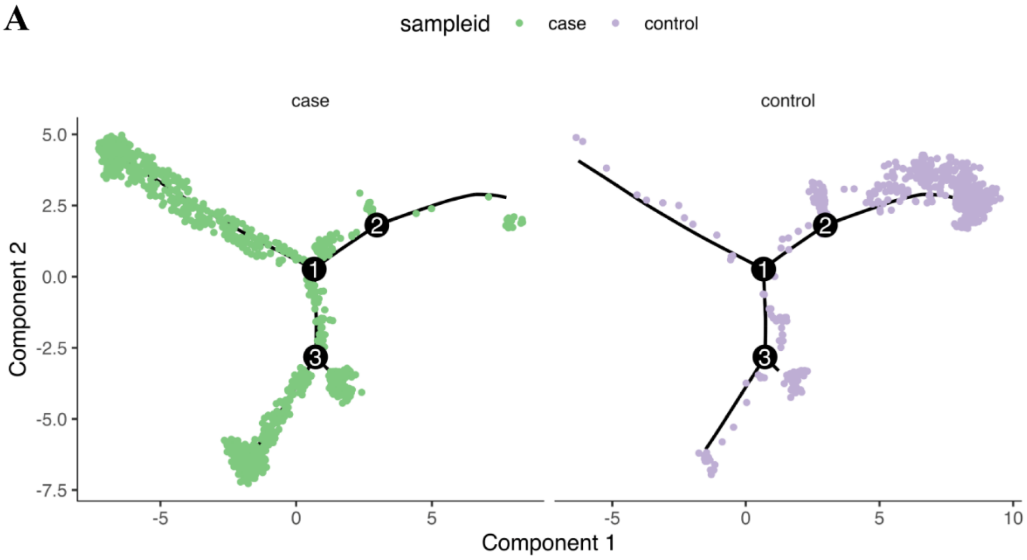

Supplemental figure 25. Pseudotime analysis of total-fibroblasts.

A

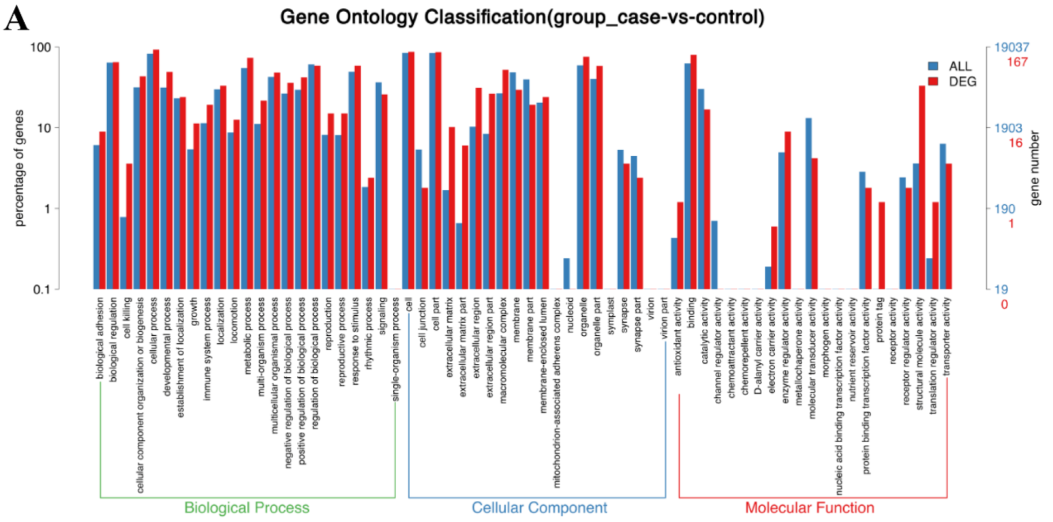

B

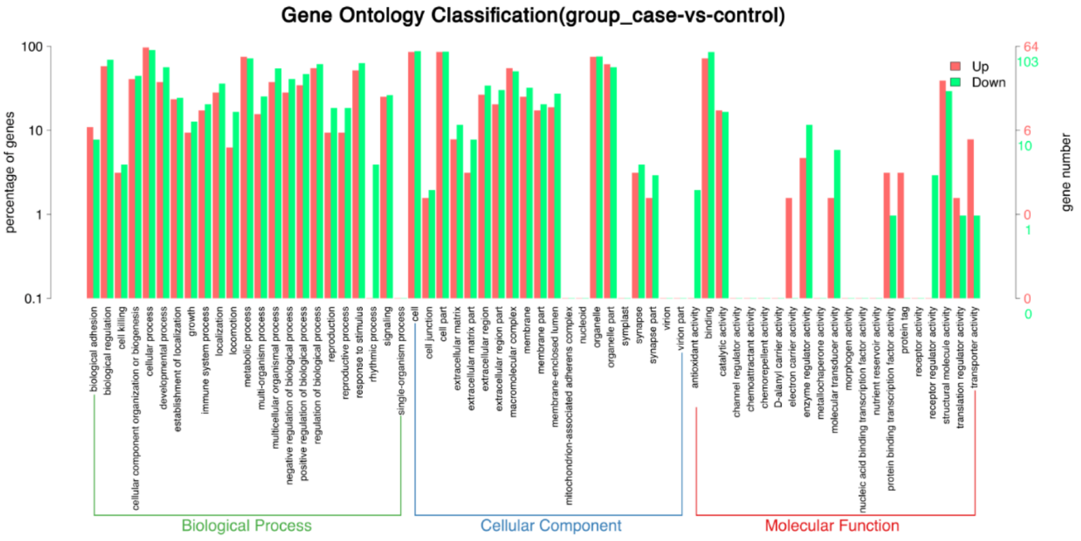

Supplemental figure 26. GO analysis of total-fibroblasts. Figure A: GO classification of differentially expressed genes between case and control groups. Figure B: GO classification of highly expressed or lowly expressed genes between case and control groups.

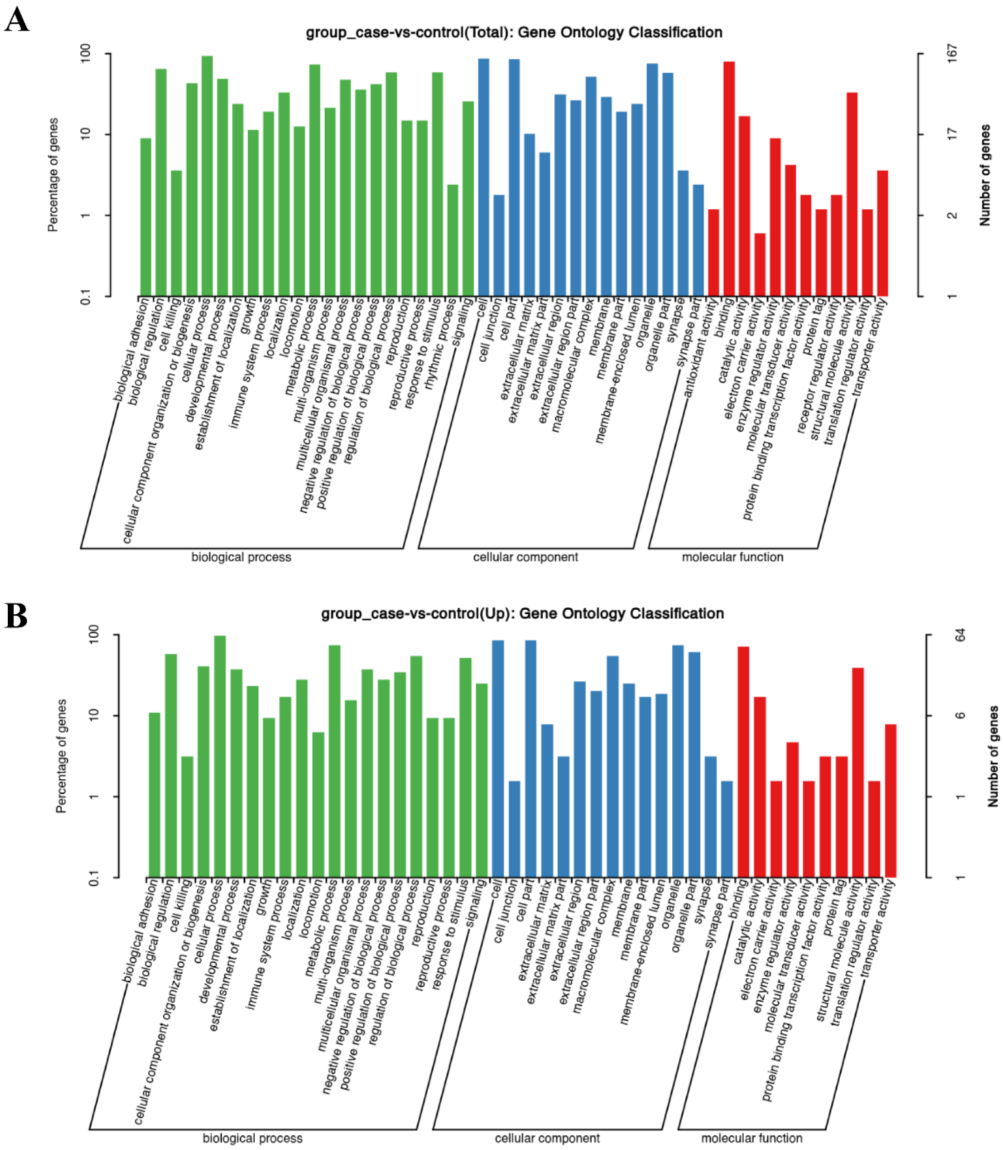

Supplemental figure 27. GO analysis of total-fibroblasts. Figure A: Gene ontology classification of differentially expressed genes between case and control groups. Figure B: Gene ontology classification of highly expressed genes between case and control groups.

A

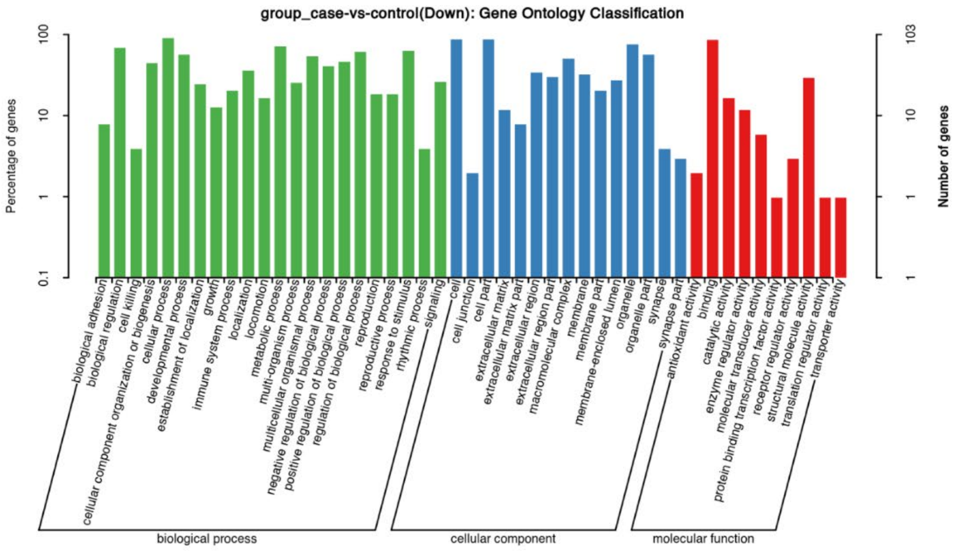

B

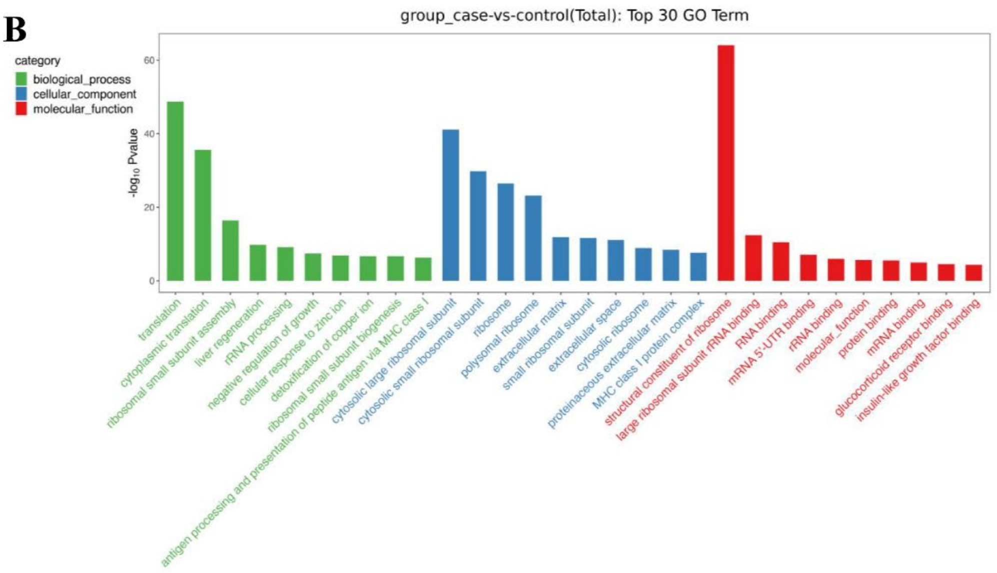

Supplemental figure 28. GO analysis of total-fibroblasts. Figure A: Gene ontology classification of lowly expressed genes between case and control groups. Figure B: Top30 GO term of total genes of both case and control group.

A

B

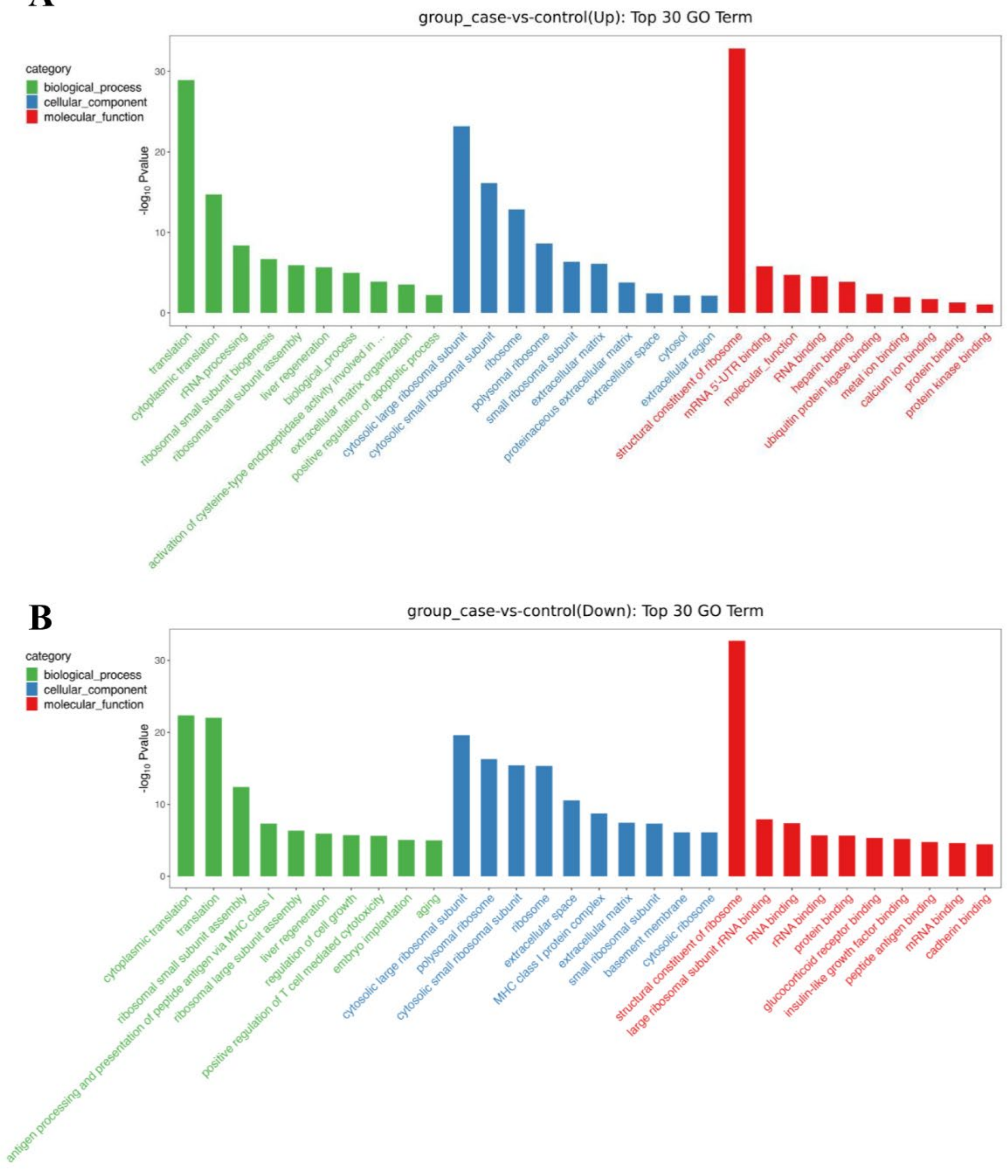

Supplemental figure 29. GO analysis of total-fibroblasts. Figure A: Top30 GO term of highly expressed genes between case and control group. Figure B: Top30 GO term of lowly expressed genes between case and control group.

A

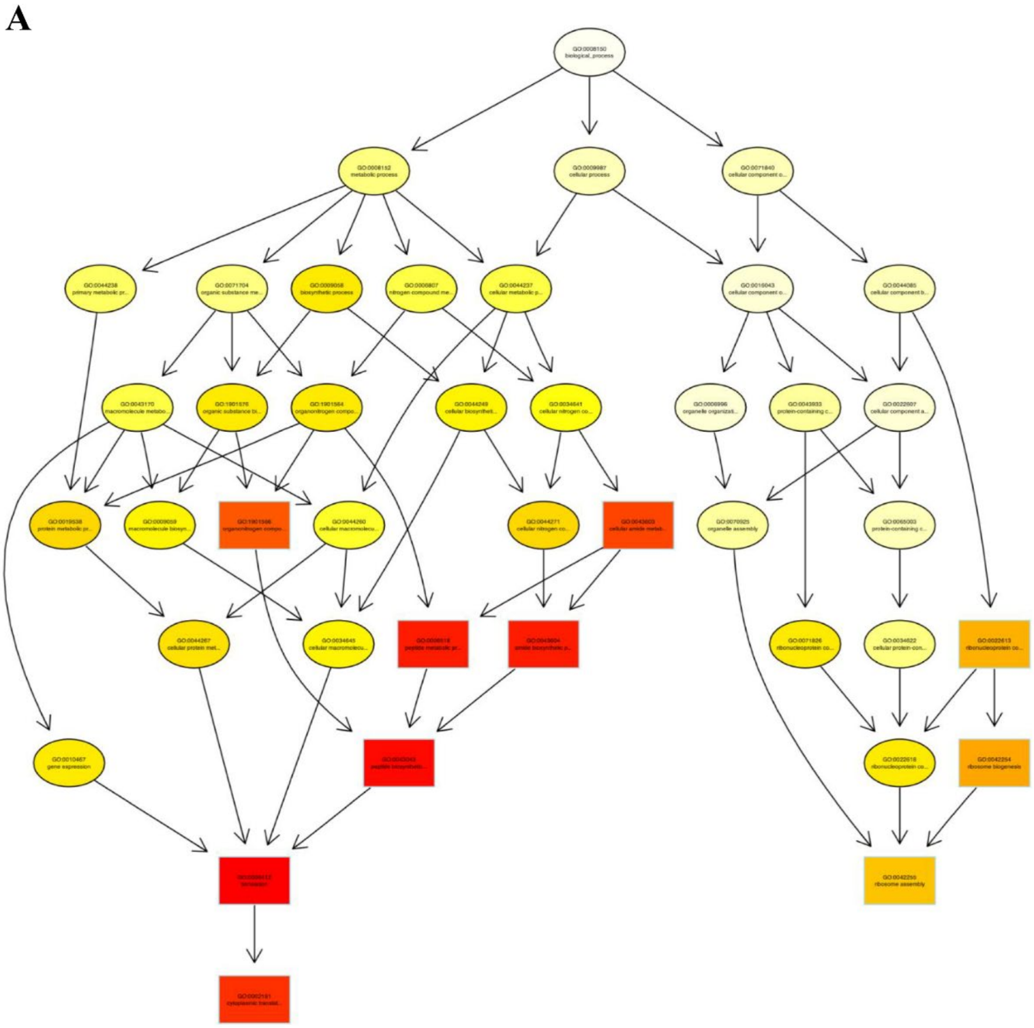

Supplemental figure 30. GO analysis of total-fibroblasts. The communication network of GO terms.

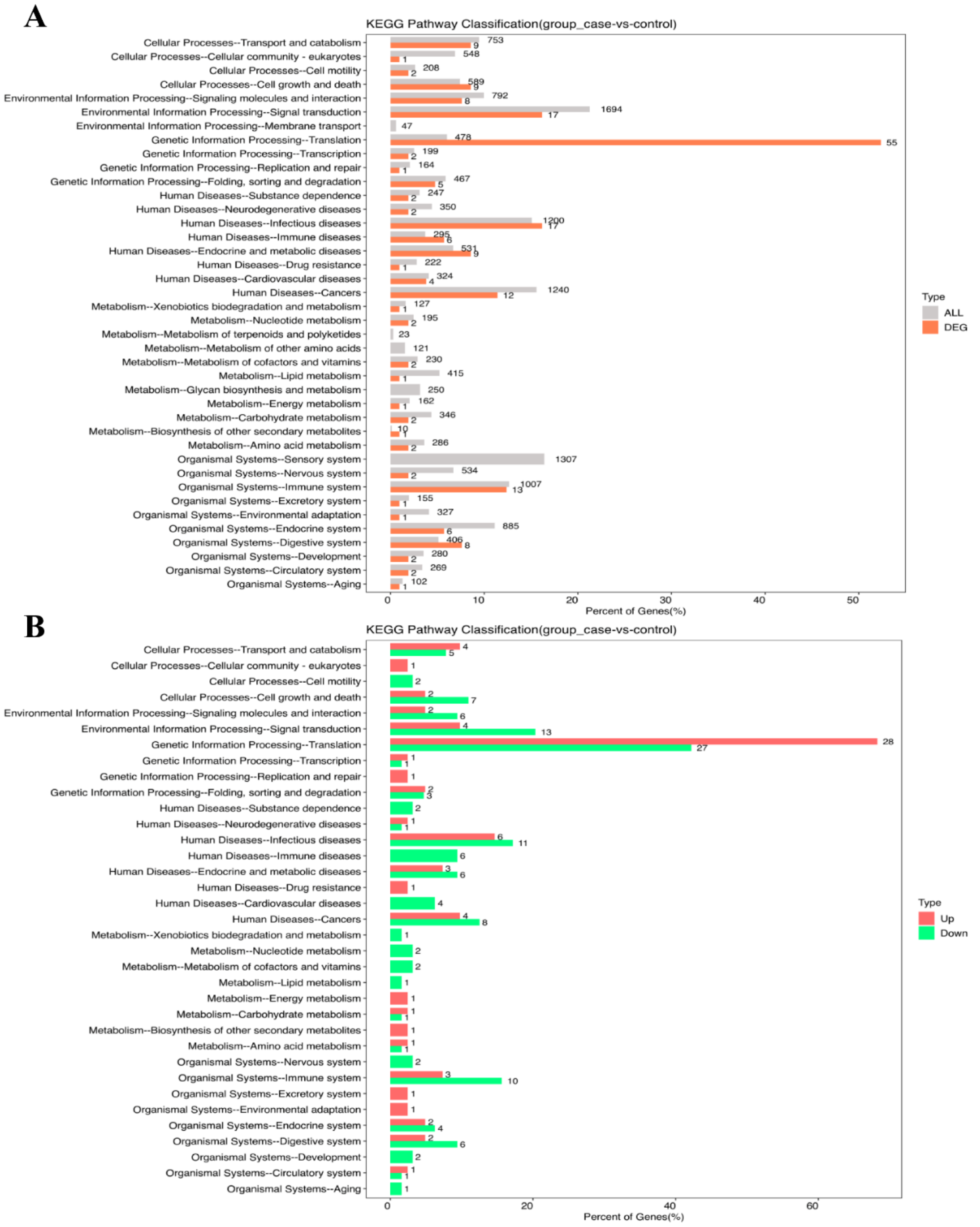

Supplemental figure 31. KEGG analysis of total-fibroblasts. Figure A: KEGG pathway classification of differentially expressed genes between case and control groups. Figure B: KEGG pathway classification of highly expressed or lowly expressed genes between case and control groups.

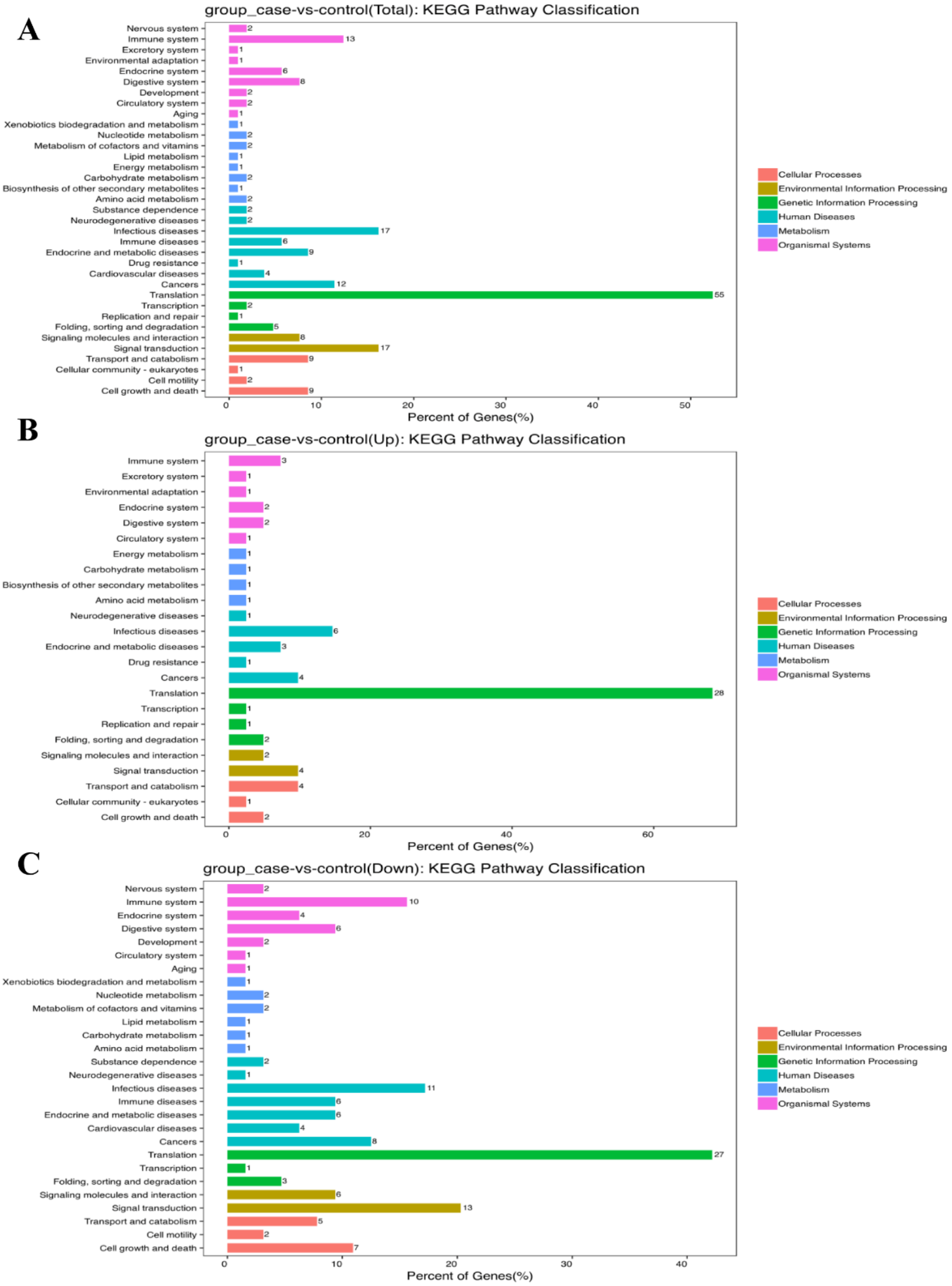

Supplemental figure 32. KEGG analysis of total-fibroblasts. Figure A: KEGG pathway classification of total genes of both case and control group. Figure B: KEGG pathway classification of highly expressed genes between case and control groups. Figure C: KEGG pathway classification of lowly expressed genes between case and control groups.

A

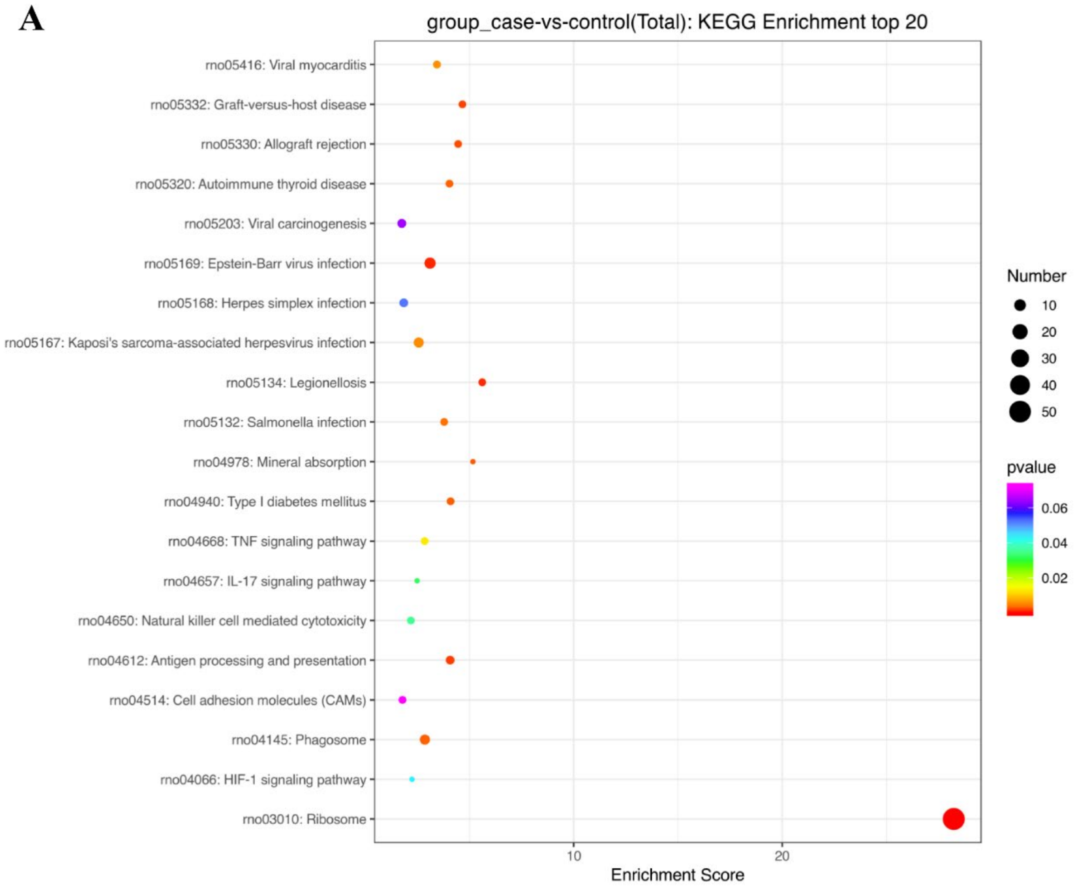

Supplemental figure 33. KEGG analysis of total-fibroblasts. KEGG enrichment top20 of total genes in both case and control group.

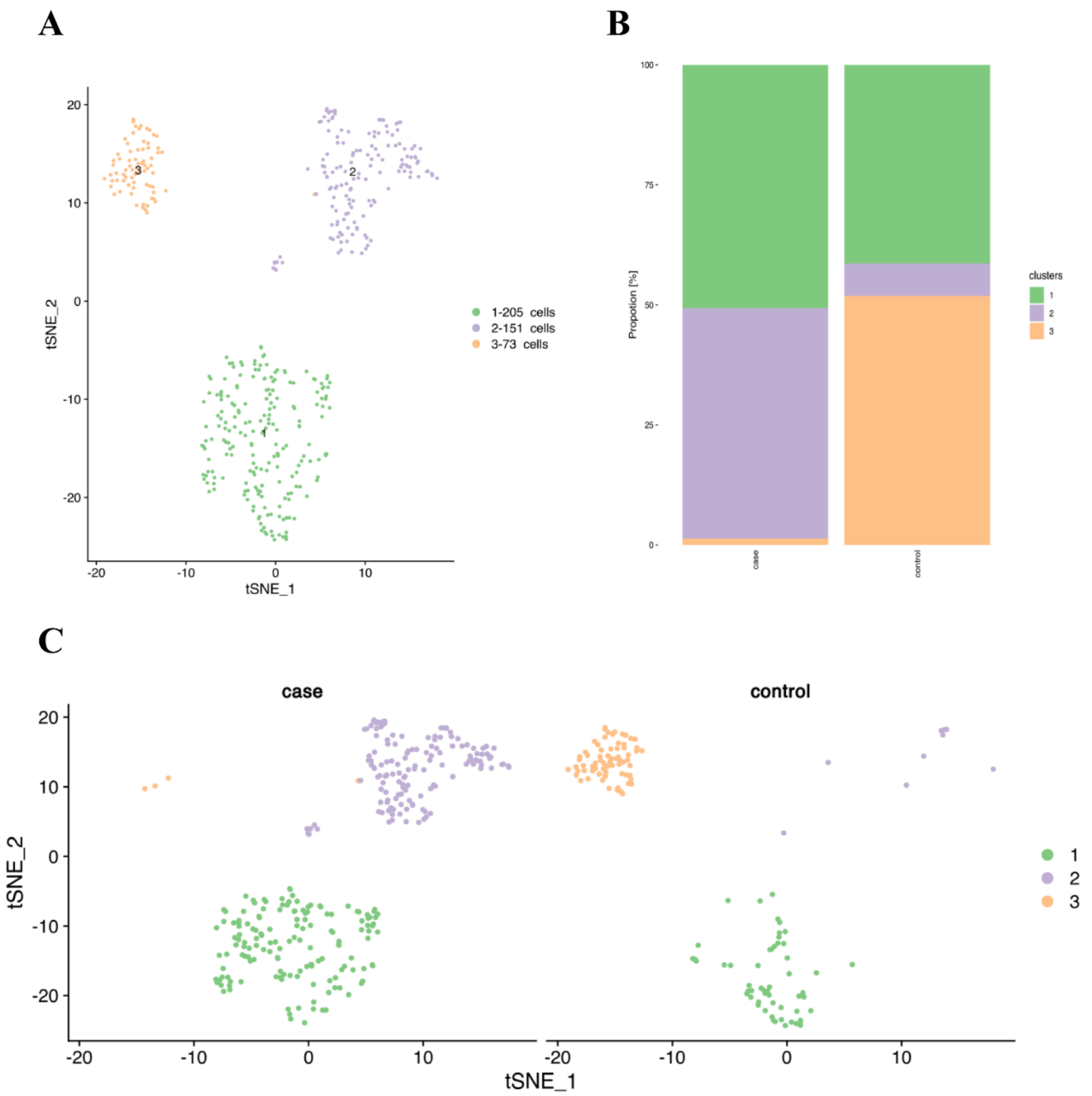

Supplemental figure 34. TSNE of total-ECs. Figure A: 3 clusters of total-fibroblasts. Figure B: Proportion of 3 different clusters. Figure C: TSNE of 3 clusters in case and control groups.

A

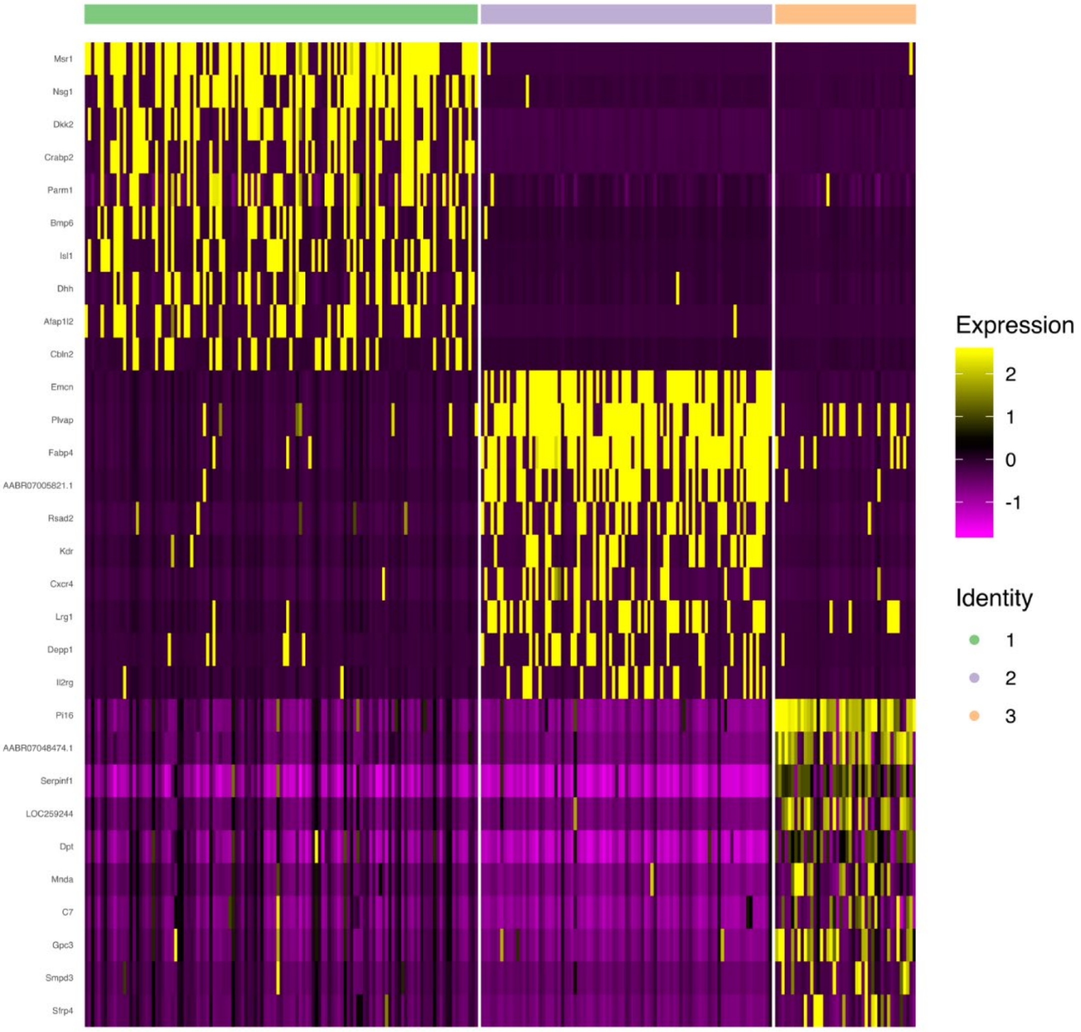

Supplemental figure 35. Top10 markers of different clusters in ECs.

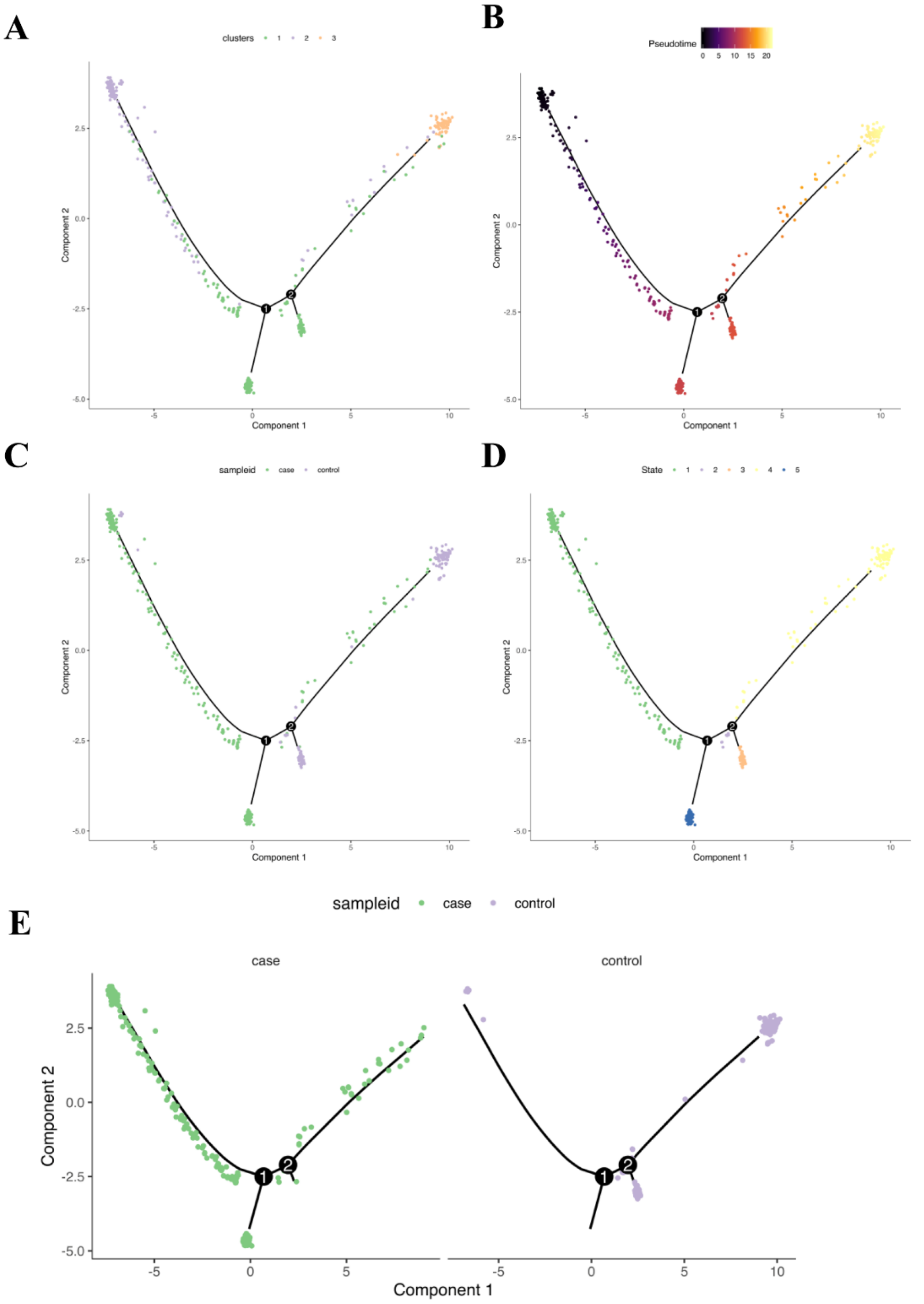

Supplemental figure 36. Pseudotime analysis of total-ECs.

A

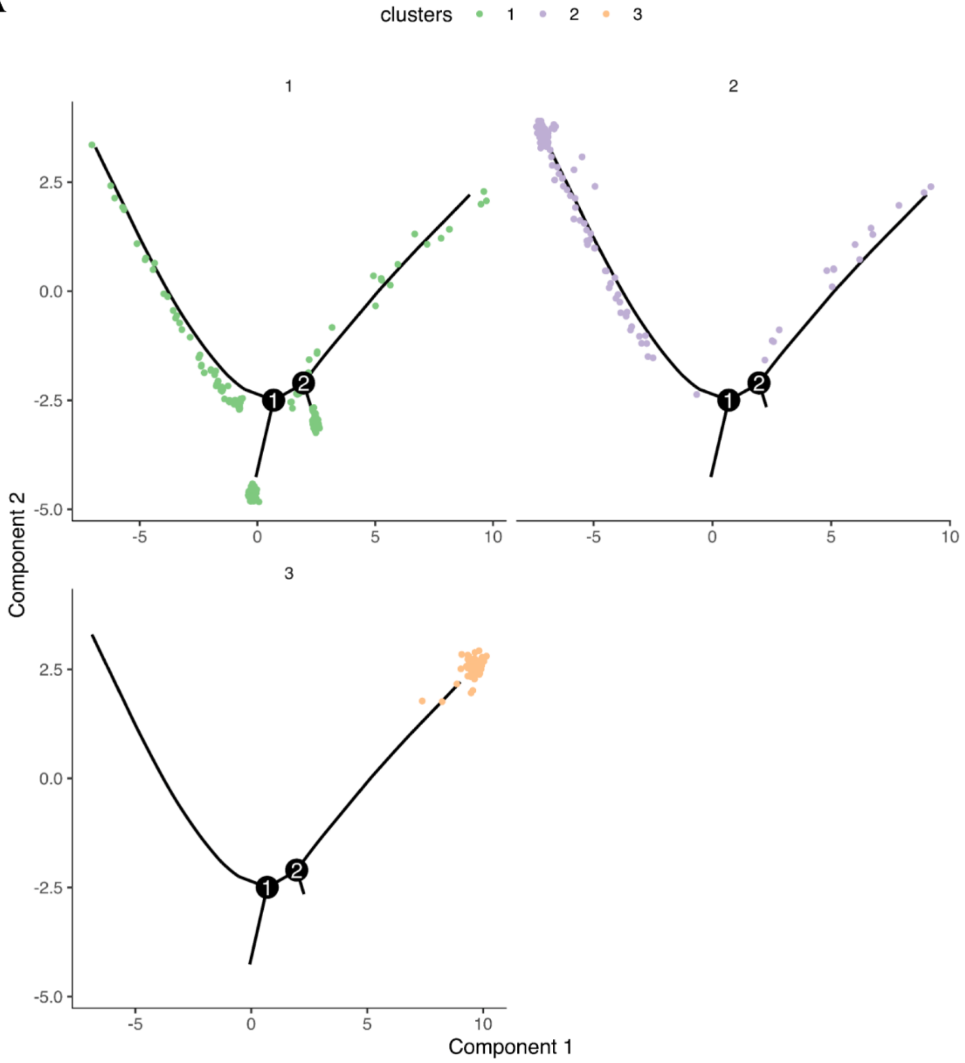

Supplemental figure 37. Pseudotime analysis of total-ECs. Split of 3 clusters in pseudotime analysis.

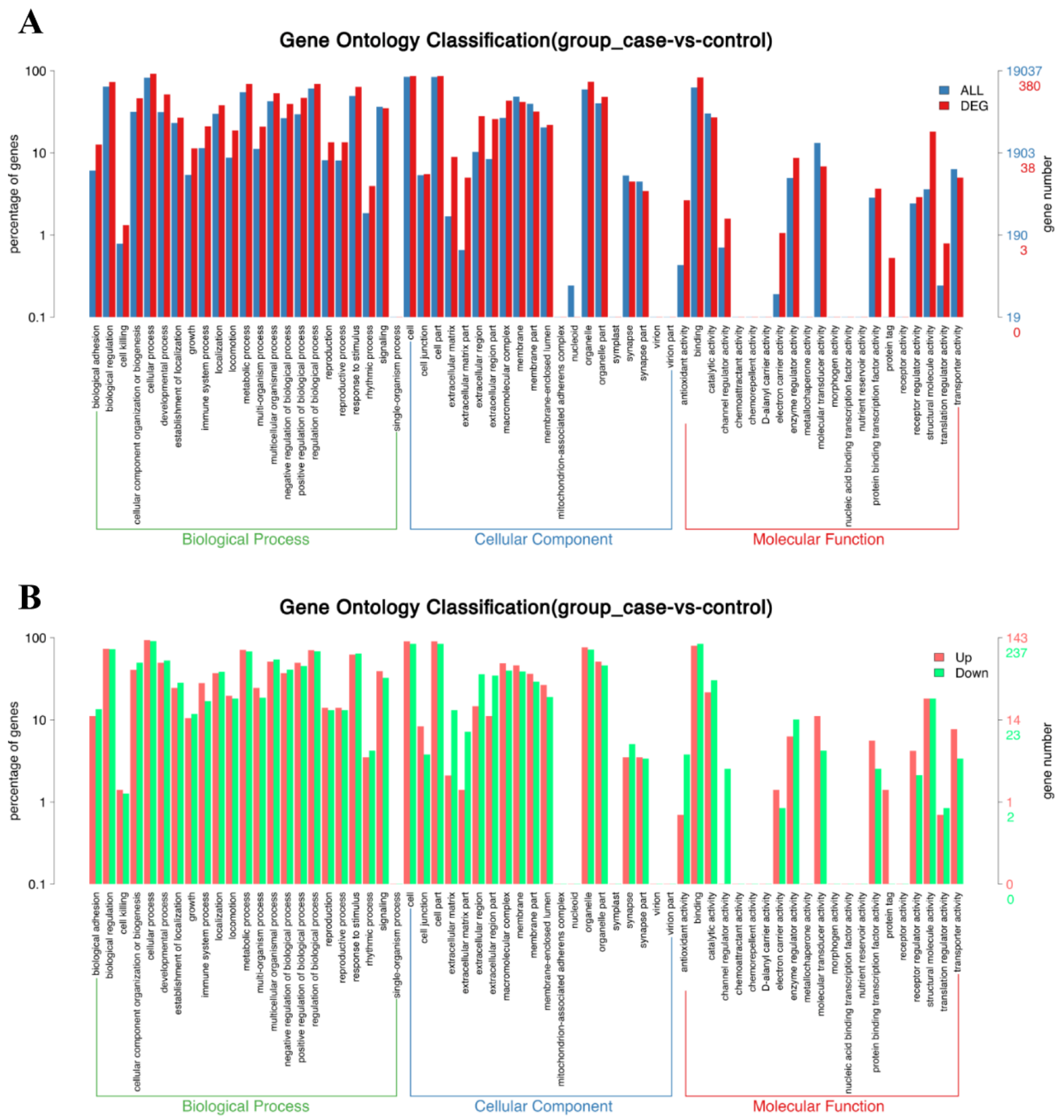

Supplemental figure 38. GO analysis of total-ECs. Figure A: GO classification of differentially expressed genes between case and control groups. Figure B: GO classification of highly expressed or lowly expressed genes between case and control groups.

A

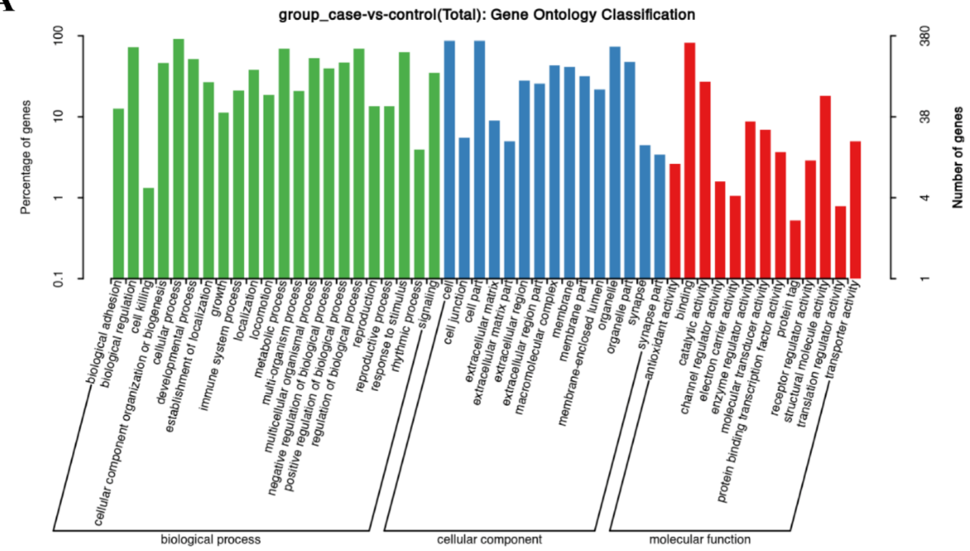

B

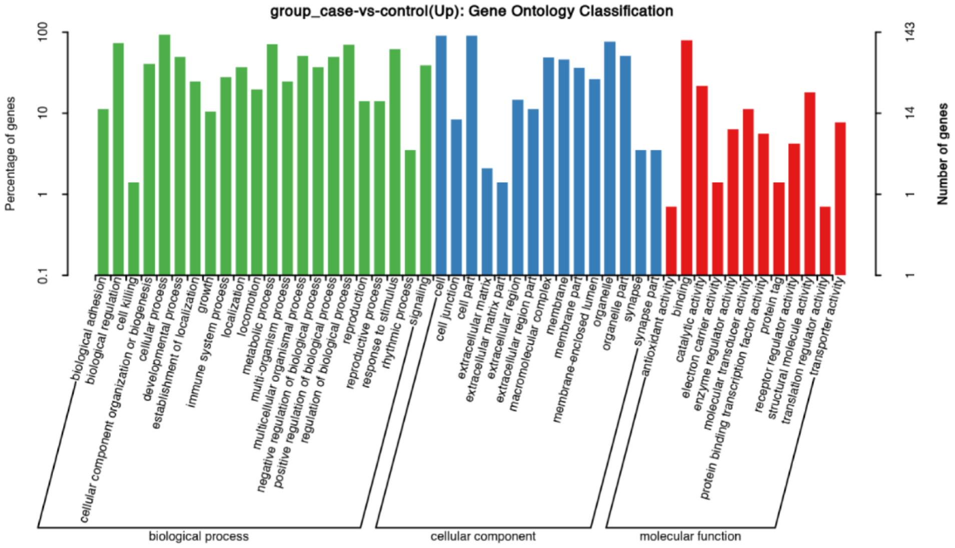

Supplemental figure 39. GO analysis of total-ECs. Figure A: Gene ontology classification of differentially expressed genes between case and control groups. Figure B: Gene ontology classification of highly expressed genes between case and control groups.

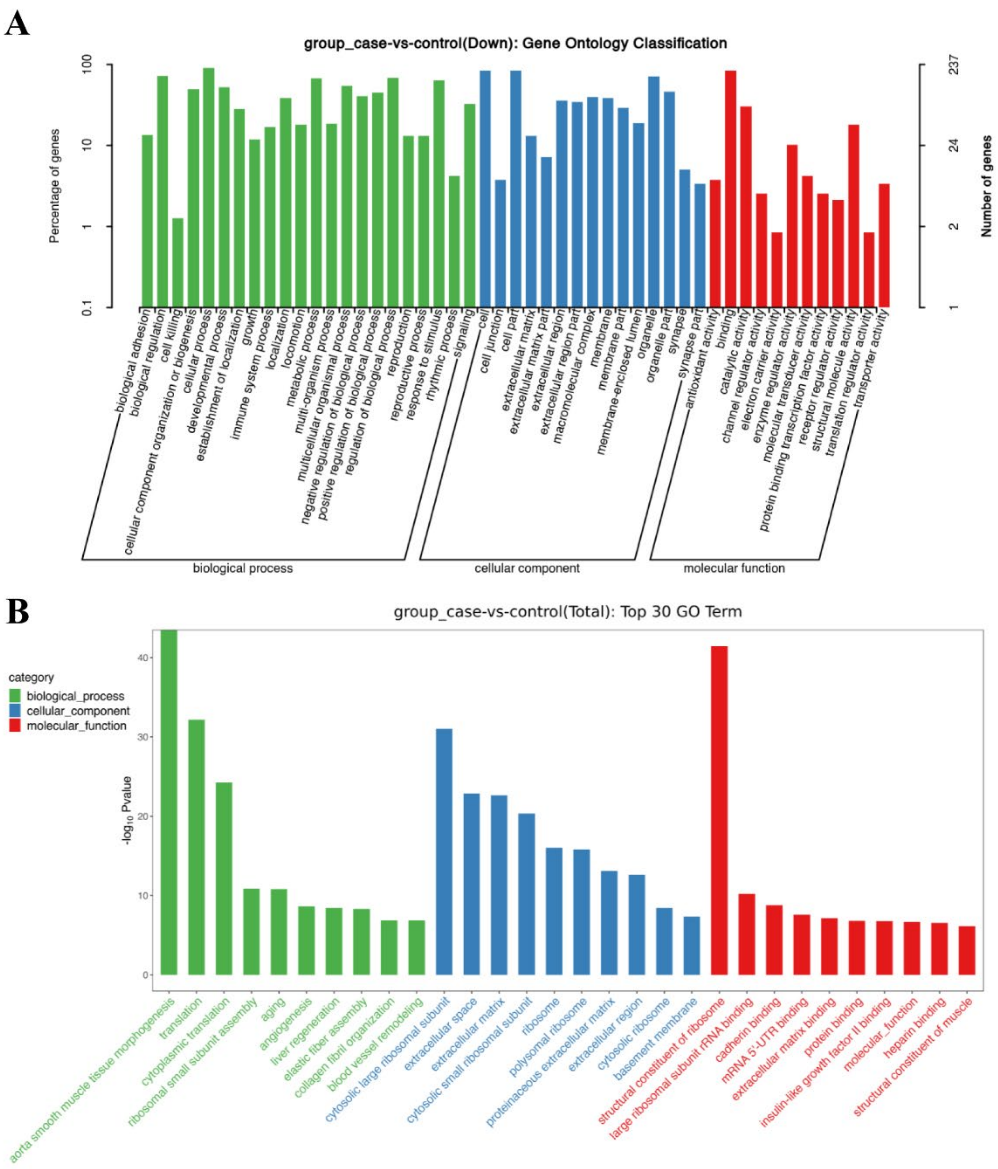

Supplemental figure 40. GO analysis of total-ECs. Figure A: Gene ontology classification of differentially expressed genes between case and control groups. Figure B: Gene ontology classification of highly expressed genes between case and control groups.

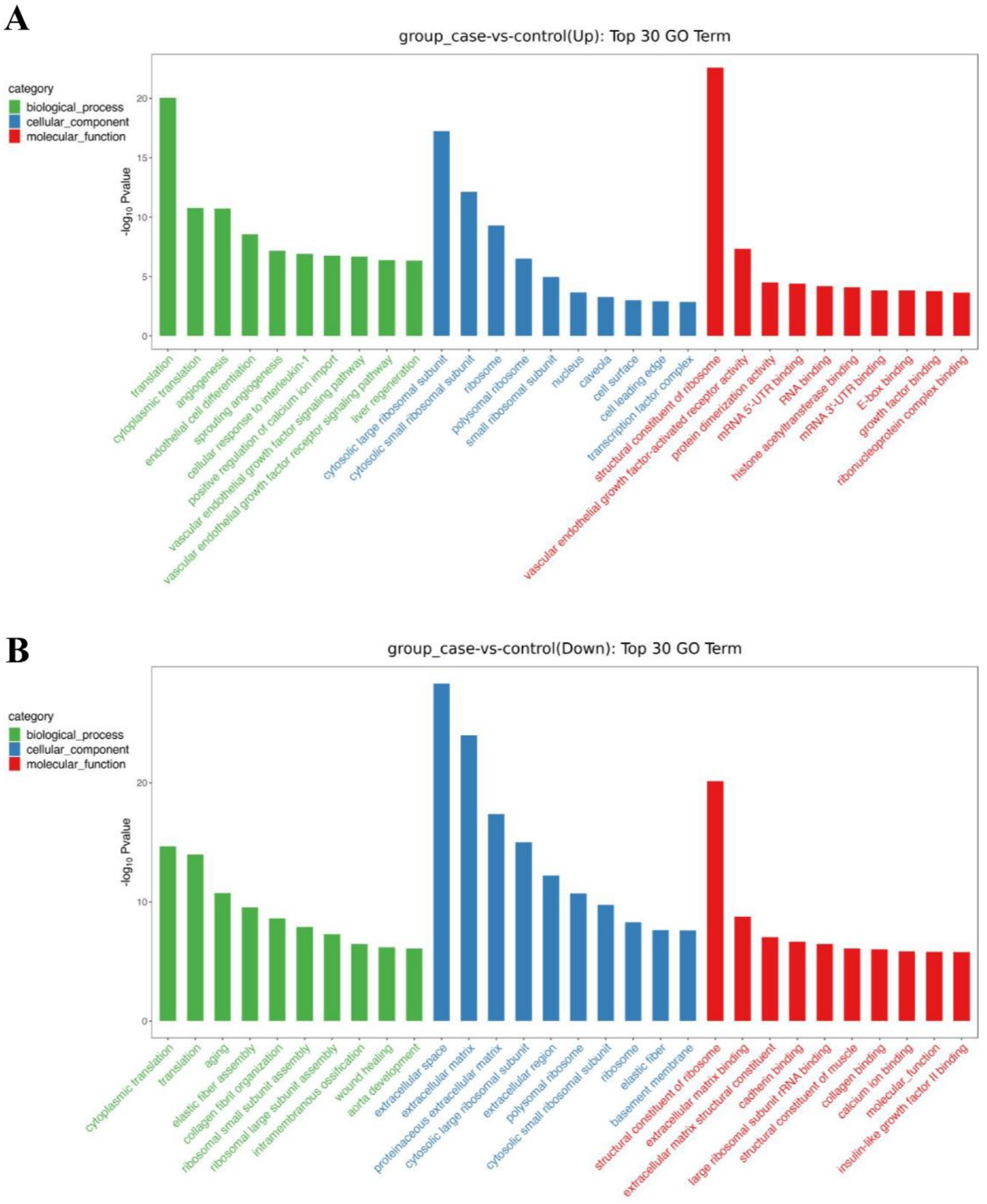

Supplemental figure 41. GO analysis of total-ECs. Figure A: Top30 GO term of highly expressed genes between case and control group. Figure B: Top30 GO term of lowly expressed genes between case and control group.

A

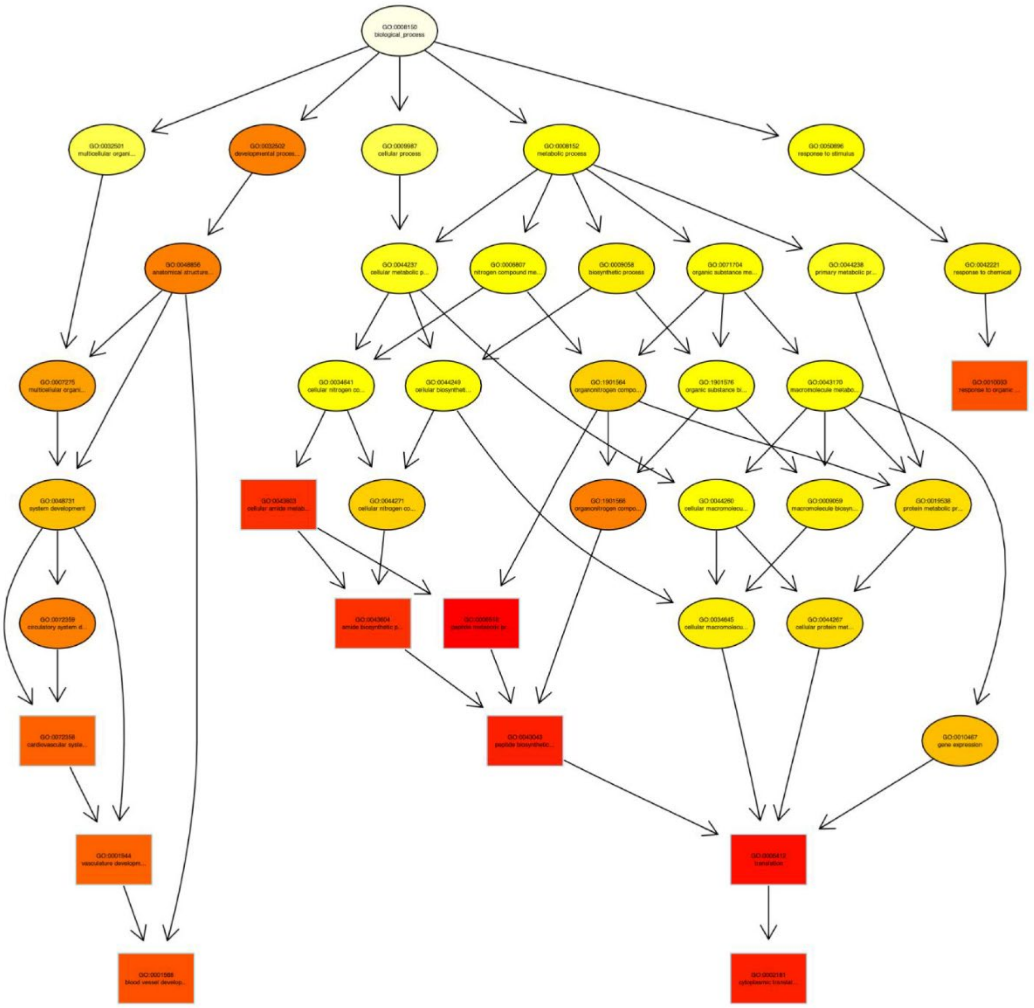

Supplemental figure 42. GO analysis of total-ECs. The communication network of GO terms.

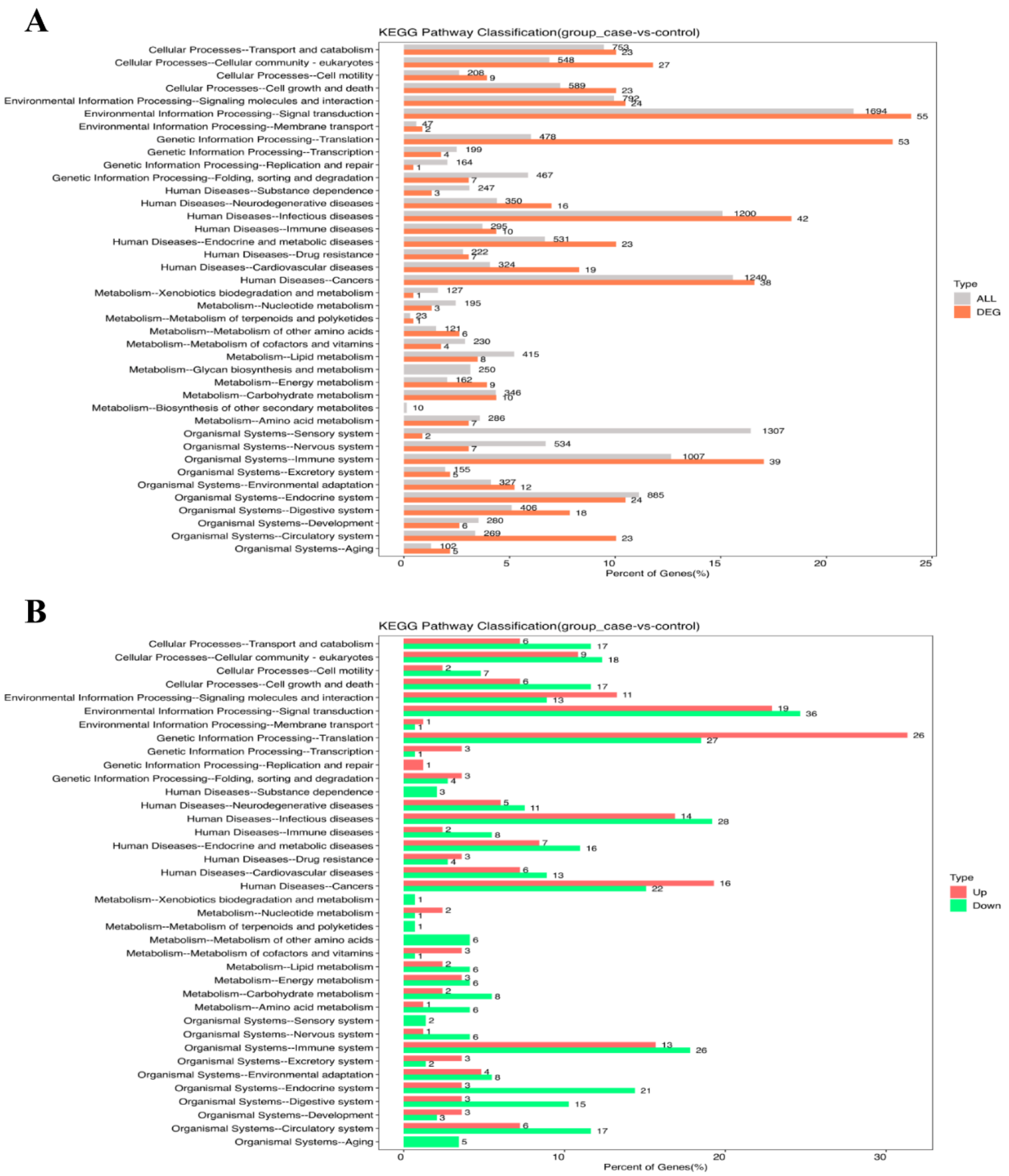

Supplemental figure 43. KEGG analysis of total-ECs. Figure A: KEGG pathway classification of differentially expressed genes between case and control groups. Figure B: KEGG pathway classification of highly expressed or lowly expressed genes between case and control groups.

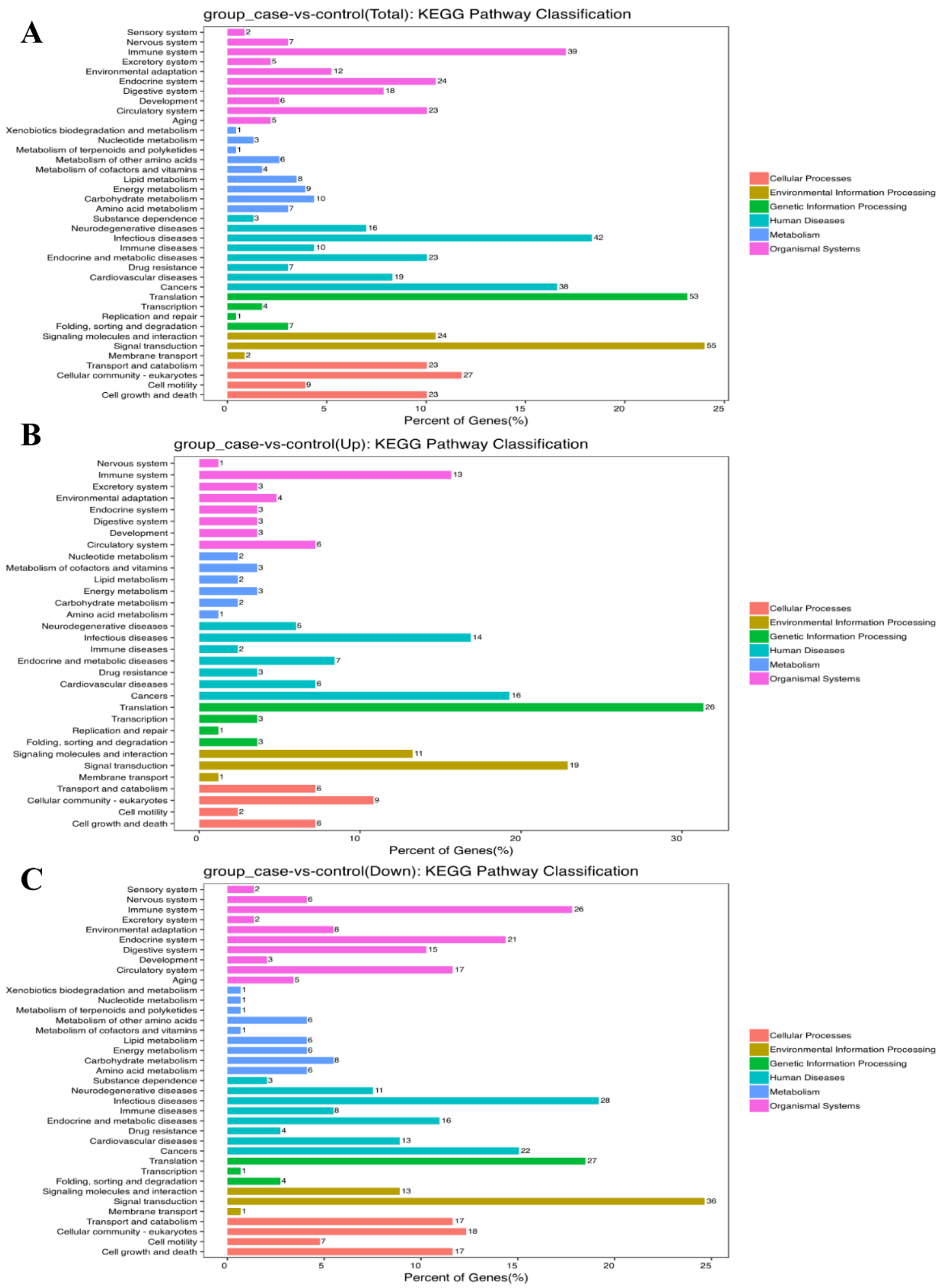

Supplemental figure 44. KEGG analysis of total-ECs. Figure A: KEGG pathway classification of total genes of both case and control group. Figure B: KEGG pathway classification of highly expressed genes between case and control groups. Figure C: KEGG pathway classification of lowly expressed genes between case and control groups.

A

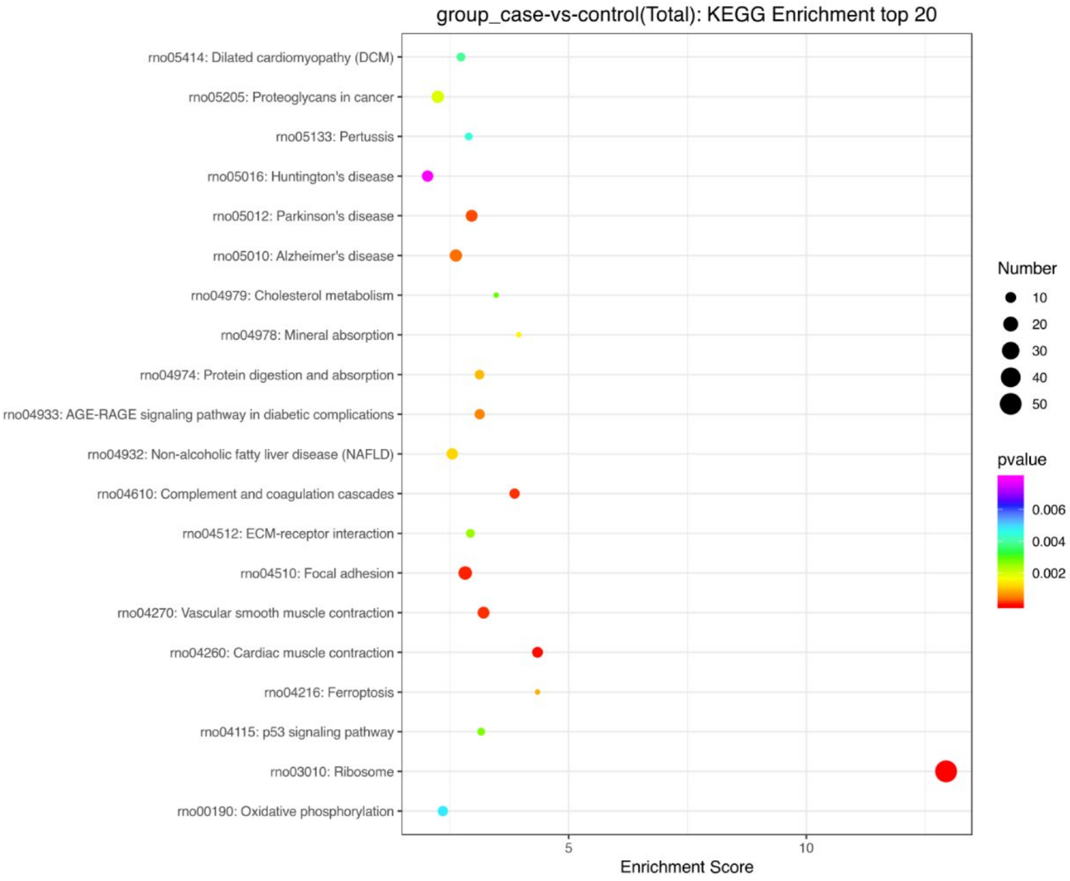

Supplemental figure 45. KEGG analysis of total-ECs. KEGG enrichment top20 of total genes in both case and control group.

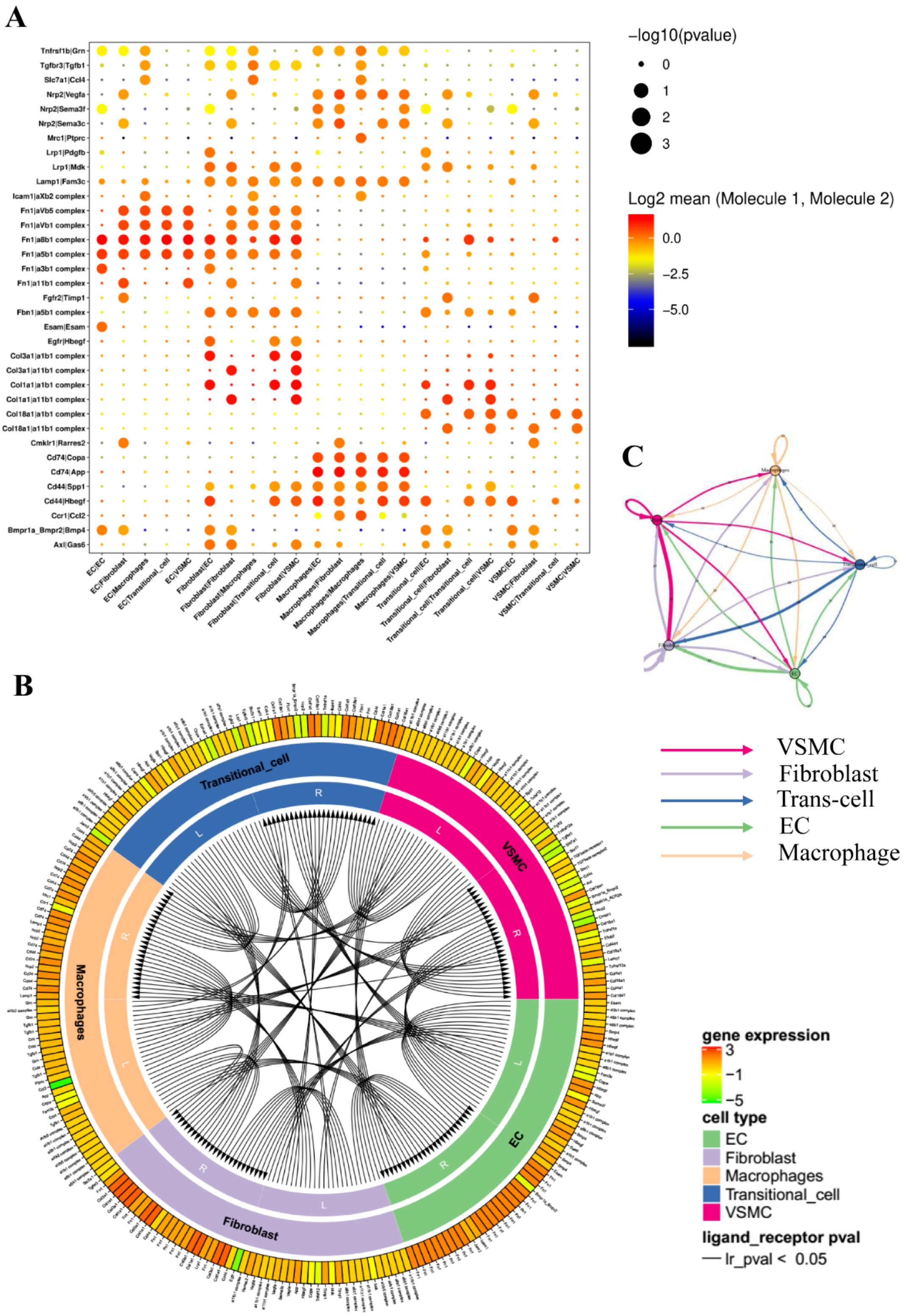

Supplemental figure 46 (control)： Figure A. Dot plot of receptors and ligands analysis. Figure B. Intercellular communication of different celltypes. Figure C. Quantitative figure of intercellular communication.
