# Supplementary material for: Single-Cell RNA Sequencing of the Rat Carotid Arteries Uncovers Potential Cellular Targets of Neointimal Hyperplasia
Source: Front Cardiovasc Med. 2021 Dec 9;8:751525. doi: 10.3389/fcvm.2021.751525 (PMC8697976; doi:10.3389/fcvm.2021.751525)
Supplement: Supplementary file 2 [file Table_1.docx]

Supplementary Material

**Supplementary Table 1.** Average values of GHG emissions (in thousands of tons per 100,000 inhabitants) and DALYs (in years per 1,000 inhabitants) in individual EU countries

| ID | K-M | PAM | GHG | CO_2_ | CH_4_ | N_2_O | HFC | SF_6_ | DALYs |
| --- | --- | --- | --- | --- | --- | --- | --- | --- | --- |
| AUT | 1 | 1 | 879.95(16) | 733.49(16) | 80.91(7) | 42.59(8) | 18.28(16) | 3.97(26) | 2.33(13) |
| BEL | 1 | 1 | 1081.86(21) | 913.64(22) | 74.38(4) | 57.62(18) | 33.55(25) | 0.91(21) | 1.88(9) |
| BGR | 1 | 1 | 697.68(12) | 506.97(12) | 103.58(17) | 70.68(21) | 16.21(14) | 0.24(7) | 4.16(17) |
| CYP | 2 | 2 | 995.16(19) | 829.22(20) | 98.93(15) | 34.93(3) | 32.06(24) | 0.02(1) | 23.87(25) |
| CZE | 1 | 1 | 1215.98(24) | 997.24(24) | 132.54(24) | 56.71(15) | 28.58(23) | 0.78(17) | 2.11(12) |
| DEU | 1 | 1 | 1085.95(22) | 948.58(23) | 71.12(3) | 48.14(12) | 13.29(9) | 3.99(27) | 0.25(1) |
| DNK | 1 | 1 | 1012.44(20) | 767.24(19) | 135.34(25) | 96.46(23) | 11.59(6) | 1.69(25) | 3.65(15) |
| ESP | 1 | 1 | 657.64(9) | 505.44(11) | 85.6(9) | 38.98(6) | 25.81(21) | 0.48(14) | 0.39(4) |
| EST | 2 | 2 | 1306.13(25) | 1108.97(26) | 94.13(11) | 87.3(22) | 15.57(12) | 0.16(3) | 18.48(24) |
| FIN | 1 | 1 | 759.04(15) | 501.21(10) | 107.06(19) | 125.93(26) | 24.32(19) | 0.47(13) | 3.74(16) |
| FRA | 1 | 1 | 671.22(10) | 483.21(8) | 91.26(10) | 67.39(20) | 27.41(22) | 0.92(22) | 0.29(2) |
| GRC | 1 | 1 | 932.6(17) | 742.75(17) | 95.86(14) | 42.99(9) | 49.76(26) | 0.05(2) | 1.87(8) |
| HRV | 1 | 1 | 469.55(3) | 314.15(4) | 95.11(13) | 49.66(13) | 10.45(3) | 0.17(5) | 5.46(20) |
| HUN | 1 | 1 | 575.89(6) | 440.17(7) | 75.88(6) | 43.99(10) | 14.83(11) | 1(23) | 2.61(14) |
| IRL | 1 | 1 | 1371.22(26) | 912.96(21) | 287(27) | 145.7(27) | 24(18) | 0.86(20) | 4.32(18) |
| ITA | 1 | 1 | 708.36(13) | 574.13(14) | 75.57(5) | 31.45(2) | 23.85(17) | 0.71(16) | 0.31(3) |
| LTU | 1 | 1 | 448.02(2) | 205.57(2) | 116.63(22) | 109.95(24) | 15.67(13) | 0.19(6) | 9.62(21) |
| LUX | 2 | 2 | 1956.89(27) | 1781.01(27) | 105.69(18) | 57.37(16) | 11.24(4) | 1.57(24) | 35.43(26) |
| LVA | 1 | 2 | 562.76(5) | 316.46(5) | 114.77(21) | 121.09(25) | 10(2) | 0.44(12) | 13.88(23) |
| MLT | 2 | 2 | 611.3(8) | 499.97(9) | 40.4(1) | 10.73(1) | 59.86(27) | 0.32(11) | 45.17(27) |
| NDL | 1 | 1 | 1197.65(23) | 1022.97(25) | 109.48(20) | 51.33(14) | 11.96(7) | 0.82(18) | 1.14(6) |
| POL | 1 | 1 | 963.73(18) | 757.93(18) | 131.1(23) | 57.43(17) | 17.07(15) | 0.16(4) | 0.63(5) |
| POR | 1 | 1 | 577.97(7) | 421.71(6) | 94.93(12) | 35.25(4) | 25.7(20) | 0.27(8) | 1.96(11) |
| ROU | 1 | 1 | 494.89(4) | 288.05(3) | 150.99(26) | 48.04(11) | 7.5(1) | 0.27(9) | 1.36(7) |
| SVK | 1 | 1 | 681.13(11) | 542.79(13) | 85.28(8) | 40.52(7) | 12(8) | 0.29(10) | 4.55(19) |
| SVN | 1 | 1 | 729.9(14) | 575.62(15) | 99.96(16) | 37.98(5) | 14.78(10) | 0.83(19) | 9.75(22) |
| SWE | 1 | 1 | 135.96(1) | 8.73(1) | 54.34(2) | 60.06(19) | 11.42(5) | 0.54(15) | 1.91(10) |

Note: Greenhouse gasses (CO_2_, CH_4_, N_2_O, HFC, SF_6_) are expressed in thousands of tons per 100,000 inhabitants and DALYs in years per 1,000 inhabitants. The number in parentheses represents the order – the lowest value is marked (1) and the highest (27).

ID – country identifier; K-M – K-Means; PAM – Partitioning Around Medoids; GHG – Greenhouse Gases (total); CO_2_ – carbon dioxide; CH_4_ – methane; N_2_O – nitrous oxide; HFC – hydrofluorocarbons; SF_6_ – sulfur hexafluoride; DALYs – disability-adjusted life years; AUT – Austria; BEL – Belgium; BGR – Bulgaria; CYP – Cyprus; CZE – Czech Republic; DEU – Germany; DNK – Denmark; ESP – Spain; EST – Estonia; FIN – Finland; FRA – France; GRC – Greece; HRV – Croatia; HUN – Hungary; IRL – Ireland; ITA – Italy; LTU – Lithuania; LUX – Luxembourg; LVA – Latvia; MLT – Malta; NDL – Netherlands; POL – Poland; POR – Portugal; ROU – Romania; SVK – Slovakia; SVN – Slovenia; SWE – Sweden.
